# Synthesis and Evaluation of a Novel Adenosine-Ribose Probe for Global-Scale Profiling of Nucleoside and Nucleotide-Binding Proteins

**DOI:** 10.1371/journal.pone.0115644

**Published:** 2015-02-11

**Authors:** Shikha Mahajan, Roman Manetsch, David J. Merkler, Stanley M. Stevens Jr.

**Affiliations:** 1 Department of Chemistry, University of South Florida, 4202 E. Fowler Ave., Tampa, FL, 33620, United States of America; 2 Department of Chemistry and Chemical Biology and Department of Pharmaceutical Sciences, Northeastern University, 360 Huntington Avenue, Boston, MA, 02115, United States of America; 3 Department of Cell Biology, Microbiology and Molecular Biology, University of South Florida, 4202 E. Fowler Ave., Tampa, FL, 33620, United States of America

## Abstract

Proteomics is a powerful approach used for investigating the complex molecular mechanisms of disease pathogenesis and progression. An important challenge in modern protein profiling approaches involves targeting of specific protein activities in order to identify altered molecular processes associated with disease pathophysiology. Adenosine-binding proteins represent an important subset of the proteome where aberrant expression or activity changes of these proteins have been implicated in numerous human diseases. Herein, we describe an affinity-based approach for the enrichment of adenosine-binding proteins from a complex cell proteome. A novel *N* ^6^-biotinylated-8-azido-adenosine probe (AdoR probe) was synthesized, which contains a reactive group that forms a covalent bond with the target proteins, as well as a biotin tag for affinity enrichment using avidin chromatography. Probe specificity was confirmed with protein standards prior to further evaluation in a complex protein mixture consisting of a lysate derived from mouse neuroblastoma N_18_TG_2_ cells. Protein identification and relative quantitation using mass spectrometry allowed for the identification of small variations in abundance of nucleoside- and nucleotide-binding proteins in these samples where a significant enrichment of AdoR-binding proteins in the labeled proteome from the neuroblastoma cells was observed. The results from this study demonstrate the utility of this method to enrich for nucleoside- and nucleotide-binding proteins in a complex protein mixture, pointing towards a unique set of proteins that can be examined in the context of further understanding mechanisms of disease, or fundamental biological processes in general.

## Introduction

Adenosine nucleosides and nucleotides, collectively referred to as adenosine ribose (AdoR) derivatives, are essential to life with key functions in cell signaling pathways, DNA and RNA biosynthesis, and cellular energy. The most significant member of the AdoR family is adenosine-5′-triphosphate (ATP), which acts as the “energy currency” of the cell. Other well-known members include adenosine, adenosine-5′-monophosphate (AMP), adenosine-5′-diphosphate (ADP), 3′, 5′-cyclic adenosine monophosphate (cAMP), *S*-adenosylmethionine (SAM), 5’-methylthioadenosine (MTA), and coenzyme A. Because of their metabolic centrality, the number of proteins and enzymes that bind an AdoR is large, with defective expression of AdoR-binding proteins correlating to many human diseases. Some examples of the AdoR-binding proteins linked to physiological disorders are adenosine A_2B_ receptor in invasive breast cancer [1], adenosine A_3_ receptor and AMP deaminase in colon cancer [2,3], nucleoside diphosphate kinase (Nm23) in highly metastatic cancers [4], ATP-binding cassette (ABC) transporters in multi-drug resistant cancer [5], ATP-dependent protein kinases in myocardial ischemia/heart failure [6], and DNA methyltransferase-1 (SAM-dependent) in schizophrenia and bipolar disorder [7]. Global approaches to identify and quantify differences in activity levels of AdoR-binding proteins between normal and diseased states are necessary to better define the role of this family of proteins. In this regard, the design and implementation of activity-based protein profiling (ABPP) probes would be one strategy to evaluate differential abundance and/or activity of AdoR-binding proteins.

**Fig 1:**
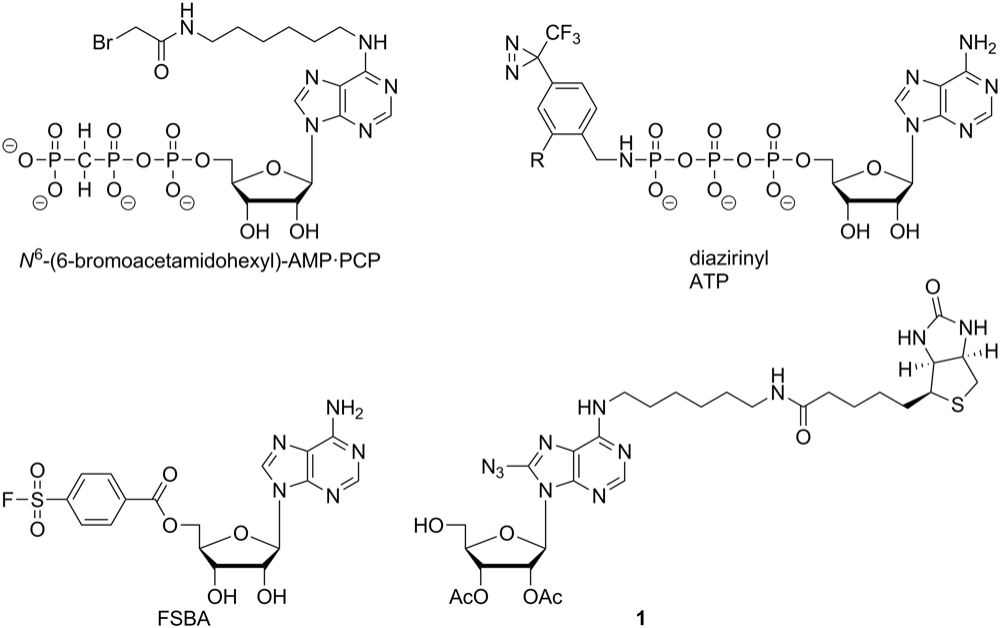
ABPP Photoaffinity Probes. Structure of the current probe **1** differs from earlier reported probes based on the labeling functionality in the 8-position versus the 5’-position.

ABPP probes are generally designed to label a reactive amino acid within the active site of a mechanistically related family of enzymes, much like the design of suicide substrates [8,9]. Consequently, previous AdoR-directed probes were adenosine analogues substituted with an electrophilic functionality in the 5′-position (Fig. 1), which served to covalently attach the probe to the AdoR-binding proteins via reactions with amino acids including tyrosine, lysine, serine, histidine, and cysteine [10–13]. For example, fluorosulfonylbenzoyl analogs of nucleotides such as 5′-p-fluorosulfonylbenzoyladenosine (5’-FSBA) have been utilized as an affinity label to modify the AdoR-binding pocket in a number of purified proteins [14]. However, proteome-wide analysis of nucleotide-binding proteins is limited by the enrichment technique used for this protein class [15]. More recently, 5’-FSBA has served as an ABPP probe to define the nucleotide-binding proteins of the human Jurkat cell proteome [16]. Another example is the set of nucleotide acyl phosphates used for the proteomic profiling of cellular kinases [14].

Although FSBA and the nucleotide acyl phosphates have facilitated the detection and profiling of AdoR-binding proteins, the utility of these probes is limited by (*a*) the unnatural 5’-reactive group which could preclude high affinity binding to some AdoR-binding proteins, and (*b*) the necessity of a reactive amino acid within the AdoR-binding pocket (to react with the probe) which could prevent the labeling of AdoR receptors or AdoR-binding allosteric sites. One alternative design circumventing the limitations of the FSBA and the nucleotide acyl phosphate probes is an AdoR-based probe with a reactive group in a non-5’-position. The reactive probe *N* ^6^-(6-bromoacetamidohexyl)-substituted adenylyl-(β, γ-methylene)-diphosphonate (AMP·PCP) was used to affinity label glyceraldehyde-3-phosphate dehydrogenase and myokinase [17]. Another probe design incorporates a photoactivatable moiety into the AdoR-targeted probe, which provides the ability to attach the probe into a chemically inert AdoR-binding pocket. Photoactivatable AdoR analogs described in the literature include the 8-azido adenosine nucleotides (8-N_3_-AMP, 8-N_3_-ADP, and 8-N_3_-ATP) and 5’-diazirine containing ATP analogs [14,18,19]. Probe **1** employed in our approach contains an 8-azido adenosine moiety as the reactive group that generates a reactive intermediate which can indiscriminately react with amino acids at the binding site of the probe and thus is less restricted than the affinity labels with electrophilic functional groups [20–22].

In this work, we describe the development and implementation of a new AdoR photoaffinity probe. The modular strategy we designed for the synthesis of the probe will facilitate the future production of any other 5’-substituted AdoR. The probe we designed consists of a *N* ^6^-biotinylated adenosine containing an azido group in 8-position (Fig. 1). The photoactivatable 8-azido group serves as the reactive group to form a covalent bond to proteins of interest, while biotin provides a means for enrichment of the probe-labeled proteins via avidin-based chromatography. The 5′-hydroxyl group in probe **1** serves as a putative chemical handle for future derivatizations into 5′-substituted AdoR probes. Proof-of-concept experiments using the new AdoR probe with an AdoR-binding enzyme, adenosine deaminase, established optimal labeling and enrichment conditions. We subsequently applied these conditions to the analysis of a complex cell lysate (N_18_TG_2_ cells) with our probe, resulting in considerably improved sensitivity for detection of AdoR-binding proteins compared to the non-labeled proteome.

## Materials and Methods

### Materials

Anti-biotin antibody HRP conjugate, 6-thioguanine, and a protease inhibitor cocktail specifically developed for mammalian cell culture lysates were from Sigma Aldrich. SuperSignal West Pico Chemiluminescent Substrate and CL-XPosure file were from Thermo Scientific. Dulbecco’s Modified Eagle Medium (DMEM) and penicillin/streptomycin were purchased from Mediatech Cellgro. Calf intestinal adenosine deaminase (cADA) was from EMD Calbiochem and sequencing grade trypsin was from Promega. Mouse neuroblastoma N_18_TG_2_ cells were purchased from DSMZ (Deutsche Sammlung von Mikroorganismen and Zellkulturen GmbH; catalog number ACC 103) [23]. All other reagents and supplies were of the highest quality available from commercial suppliers.

### Probe Design and Synthesis

**Fig 2:**
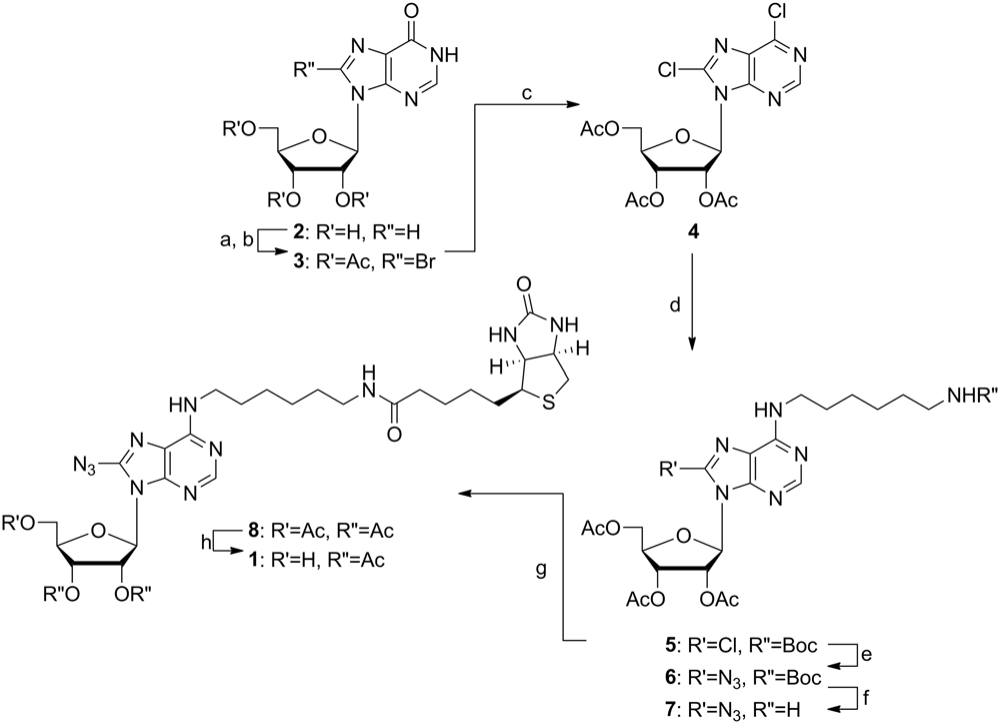
Synthesis of AdoR Photoaffinity Probe 1. Reaction conditions: a) Ac_2_O, DMAP, dry DCM, 0°C, RT, 8 h, 95%; b) aq. Br_2_, 10% Na_2_HPO_4_, dioxane, RT, 6–7 d, 92%; c) dry DCM, POCl_3_, *N*, *N*-diethylaniline, reflux, 10 min, 78%; d) mono-*N*-Boc-1,6-diaminohexane, dry EtOH, -10°C, 12 h, 66%; e) CsN_3,_ TMSN_3_, dioxane/H_2_O, 3 d, 79%; f) TFA, DCM, RT, 4 h, 81%; g) biotin, EDCI, DMAP, dry DCM, RT, 8 h, 72%; h) NH_3_, MeOH, -40°C, 2 h, preparative HPLC, 61%.

For the success of the underlined study, the synthesis of adenine analogues has to be high yielding and straightforward. Although various synthetic protocols for modification of the adenosine base at C8 and *N* ^6^ have been reported, the attempt to combine the reactions to introduce the *N* ^6^ linker and C8 azido group in the nucleobase resulted in either no or poor yields. Synthetic challenges towards the preparation of probe **1** were encountered initially while attempting to introduce the mono-*N*-Boc-1, 6-diaminohexane or its biotinylated version in 6-position of 8-azido-inosine as the diaminohexane linker would react and substitute with the 8-azido group. Subsequently, all attempts to install the intact biotin-diaminohexane moiety prior to the generation of the 8-azido group failed due to poor yields and significant purification difficulties. Nevertheless, AdoR photoaffinity probe **1** was finally synthesized starting from commercially available inosine (Fig. 2). Inosine **2** was protected using acetic anhydride and DMAP in anhydrous dichloromethane furnishing 2’, 3’, 5’-triacetyl-inosine, which was subsequently converted to 2’, 3’, 5’-triacetyl-8-bromopurine **3** using aqueous bromine solution [24]. Next, the 2’, 3’, 5’-8-bromo derivative **3** was chlorinated to the 6, 8-dichloro intermediate **4** [25, 26], and then predominantly substituted at 6-position with mono-*N*-Boc-1, 6-diaminohexane at -10°C to give the product **5**. Purification of the desired product **5** by preparative reversed phase HPLC removed minor amounts of the undesired isomer substituted in 8-position with the mono-*N*-Boc-1, 6-diaminohexane. Generation of the 8-azido group was accomplished by reacting **5** in combination with cesium azide and trimethylsilyl azide in dioxane under reflux providing compound **6**. The progress of this reaction was carefully monitored by LC/MS until starting material **5** was completely consumed. The deprotection of the *N*-Boc protective group in compound **6** was accomplished with trifluoroacectic acid yielding primary amine **7**, which without any purification was directly coupled to biotin using EDCI/DMAP generating the *N* ^6^-biotinylated-8-azido-adenosine **8**. The 5’-acetyl protective group of **8** was selectively hydrolyzed using 2 M methanolic ammonia at -40°C to yield the desired photoaffinity probe **1** after preparative reversed-phase HPLC purification. Further details of the synthesis and product characterization are provided in supporting information, S1 Document.

### Probe Evaluation using Adenosine Deaminase

The labeling of calf intestinal adenosine deaminase (cADA, a commercially available AdoR binding protein of high quality) with probe **1** was used for proof-of-concept experiments. A 2 μM cADA solution prepared in cold PBS buffer was treated with 10 μM probe **1** in PBS buffer for a total volume of 500 μL in a quartz cuvette. The solution was exposed to UV light for variable amount of time (0–300 s) and then diluted with cold PBS solution (500 μL) to a final volume of 1.0 mL. UV irradiation was accomplished by placing the sample in front of the UV light beam, with a >295 nm UV filter on the UV source to eliminate any light with a wavelength <295 nm. The diluted solution was concentrated using a 10 kDa ultrafilter to remove unreacted probe **1** from the probe-labeled cADA and analyzed by Western analysis using a commercially available anti-biotin antibody. Protection against the labeling of cADA with probe **1** was carried out similarly by the addition of either nebularine **9** (in molar ratios of nebularine/cADA of 0 to 800) or 8-azidoadenosine **10** (in molar ratios of 8-azidoadenosine/cADA of 0 to 100). Nebularine **9** and 8-azidoadenosine **10** are AdoR analogs.

### Probe 1 Labeling of AdoR-binding Proteins on a Proteome-wide Scale

N_18_TG_2_ cells were grown at 37°C at 5% CO_2_ in DMEM supplemented with 100 μM 6-thioguanine, 100 I.U./mL penicillin, 1.0 mg/mL streptomycin, and 10% (v/v) FBS in 225 cm^2^ culture dishes. Cultures were grown to 85–90% confluence, washed with PBS, and released from the culture dishes by trypsinization. Cells were collected by centrifugation, rinsed with PBS, and re-pelleted to remove the supernatant. The soluble proteome from the N_18_TG_2_ cells was prepared from 5–6 × 10^7^ confluent cells by lysis in cold 20 mM Tris-HCl (pH 7.4), 2 mM MgCl_2_, 1 mM EDTA, 1 mM EGTA, 150 mM NaCl, 1% (v/v) Triton X-100, and 1% (v/v) of a commercial protease inhibitor cocktail. Cell disruption by sonication was carried out using a microprobe sonicator (set at 80% of full output power) for 30 s interval followed by 30 s cooling on ice for a total of 5 min. Cellular debris was removed by centrifugation (5 min at 21,000 × g) and the resulting supernatant collected as the soluble N18TG2 cell proteome. The protein concentration of the soluble proteome was determined via the Bradford dye binding assay using BSA as the standard [27].

**Fig 3:**
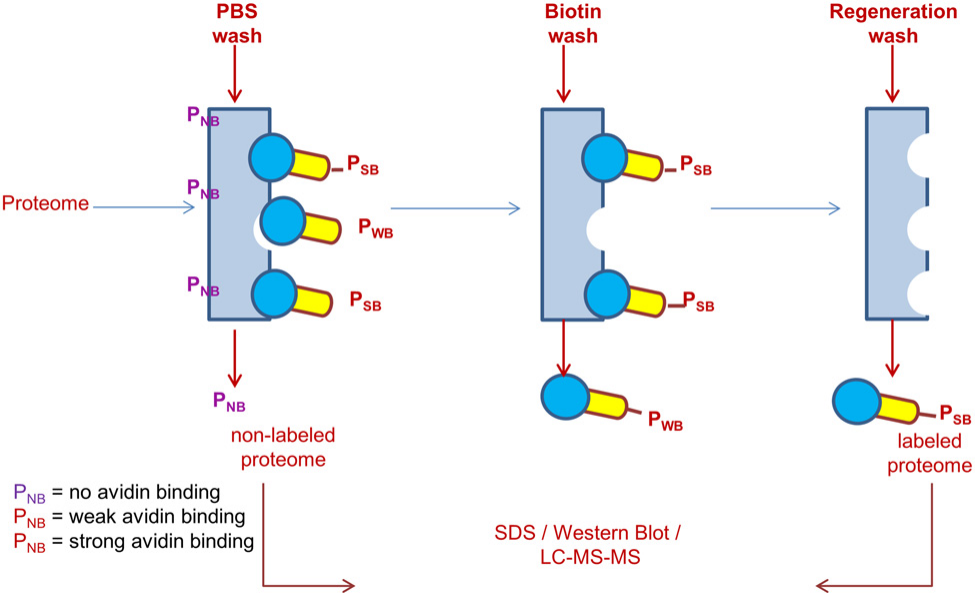
Protocol for Isolation of Labeled Proteome from Neuroblastoma Cell Lysate using Probe 1. Cell lysate sample treated with probe 1 under UV was loaded on monomeric avidin column and washed with PBS to elute non-bound proteome, 2mM biotin to elute weakly bound proteome and 2.5M glycine (pH = 2.8) to elute strongly bound proteome.

To eliminate false negatives, endogenous avidin-binding proteins were removed from the soluble N18TG2 cell proteome by incubation with monomeric avidin beads following the manufacturer’s instructions (Pierce Monomeric Avidin Kit #20227). The soluble proteome with endogenous avidin-binding proteins removed was diluted with PBS to a 1 mL total volume in a quartz cuvette with final protein concentration of 20 μM based on the average molecular weight of 50 kD. Sufficient probe **1** was added in a small volume such that the molar ratio of [probe **1**]/[total protein] = 10 and the mixture irradiated for 2 min with >295 nm UV light with manual mixing of the solution at 30 s and 90 s. Post-irradiation, the sample was immediately diluted with 1 mL of cold PBS and the diluted solution was concentrated using a 10 kDa cutoff ultrafilter to remove any non-reacted probe from the labeled proteome. The flow-through from the ultrafilter was discarded and the concentrated protein retentate was loaded onto an avidin affinity column. The protocol for isolation of labeled proteome from the N18TG2 cell lysate using probe **1** is illustrated in Fig. 3. The avidin affinity column separated biotinylated proteins conjugated to probe **1** from the non-biotinylated proteins of the proteome. The non-biotinylated proteins should have little to no affinity for avidin. Hence, these can be easily eluted from the avidin column with PBS and are collected as the “**PBS Wash**”. A concentrated biotin solution (2 mM) poses competition to the biotinylated proteins labeled with probe **1** at the binding sites on avidin column and should elute the biotinylated proteins that are relatively weakly bound to the column. These are collected as the “**Biotin Wash**”. The biotinylated proteins conjugated to probe **1** that are strongly bound to the avidin column *cannot* be eluted by the use of 2 mM biotin solution. These were eluted by washing the column with a 0.1 M glycine solution at pH 2.8 that disrupts the biotin-avidin interactions. This ensemble of proteins is collected as the “**Regeneration Wash**”. All the separately collected washes, i.e. the PBS Wash, the Biotin Wash, and the Regeneration Wash, were concentrated in 3 different 10 kDa ultrafilters at 4°C to reduce the final volume to approximately 100 μL. The protein concentration of all three samples was determined using the Bradford dye binding assay.

### Western Analysis of Labeled Proteome

One-dimensional SDS polyacrylamide gel electrophoretic (SDS-PAGE) analysis was performed on the PBS Wash, Biotin Wash, and Regeneration Wash samples. Aliquots of approximately 50 μL of each sample with an equal concentration of protein (~1 mg/mL) were prepared. To all samples, 10 μL of 5 × Laemmli Buffer containing 60 mM Tris-Cl (pH 6.8), 2% (w/v) SDS, 10% (v/v) glycerol, 5% (v/v) β-mercaptoethanol, and 0.01% (v/v) bromophenol blue was added, and then boiled for 5 min to denature the proteins.

Approximately, 2 μg of purified protein and 10 μg of total protein from labeled and non-labeled fractions were loaded on a 10% SDS-PAGE gel and the gel was run for 30 minutes. The resulting gel was electroblotted to a PVDF membrane at 70 V for 60–90 minutes at 4°C. The membrane was then blocked using 5% (v/v) non-fat dry milk in Tris-buffered saline Tween 20 (TBST) buffer for 1 hour at room temperature followed by incubation with the anti-biotin antibody HRP conjugate in 1:5000 dilution in 20 mL 1% (v/v) non-fat dry milk in TBST buffer at 4°C overnight. The membrane was washed with TBST (3 × 20 mL) for 10 min each. The membrane was then developed using SuperSignal West Pico Chemiluminescent Substrate and exposed to CL-XPosure Film for 1 min.

### Sample Preparation for LC-MS/MS Analysis of Proteins

A small aliquot of the different protein wash samples from the avidin column was used for protein identification. Approximately 20 μg of protein from three wash fractions (PBS Wash, Biotin Wash, and Regeneration Wash, respectively) were subjected to electrophoretic separation on a 10% SDS-PAGE gel. The SDS-PAGE gel was stained using Coomassie blue to visualize the ensemble of protein bands. Gel slices were excised, washed and destained completely prior to reduction using 45 mM dithiothreitol at 55°C for 30 min. The reducing agent was then replaced by freshly prepared 100 mM iodoacetamide (IAA) for carbamidomethylation in the dark at room temperature for 30 min. The gel pieces were washed 3 times with 50% acetonitrile/50 mM ammonium bicarbonate for 15 min and in-gel proteolytic digestion was performed using sequencing-grade trypsin in 50 mM ammonium bicarbonate overnight at 37°C with a total protein:trypsin ratio of 20:1 (w:w). Extracted peptide digests were desalted using reversed-phase C_18_ spin columns and the volume of the trypsin digest was then reduced by evaporation. The final volume was adjusted to 40 μL with 0.1% formic acid.

### Mass Spectrometry and Data Analysis

Tandem mass spectrometric analysis was performed on the total cell lysate (without probe-based enrichment), probe **1**-labeled proteins (Biotin Wash and Regeneration Wash), and the avidin column flow-through (PBS Wash) using an LTQ Orbitrap XL (Thermo) mass spectrometer. A 5 μL aliquot of the trypsin-digested sample was injected on a 10 cm × 75 μm i.d. reversed phase column packed with 5 μm, 300 Å C_18_ material (New Objective, Woburn, MA) and separated by nanoflow liquid chromatography employing a 90 min linear gradient from 2 to 40% ACN at 250 nL/min after inline desalting for 5 min on a capillary trap with the same C_18_ material. Orbitrap survey scans were acquired at a mass resolving power of 60,000 at m/z 400. The top 10 most abundant ions were selected for MS/MS via collision-induced dissociation in the LTQ ion trap. Raw files were processed and searched against the Mouse Uniprot database containing both forward and randomized sequences using the MASCOT version 2.2.06 (Matrix Science, Boston, MA) search algorithm. Constant modification of cysteine by carbamidomethylation and the variable modification of methionine oxidation were specified as modifications in the database search. Mascot searches were conducted with a fragment ion mass tolerance of 0.80 Da and a parent ion tolerance of 10.0 ppm including the possibility of one missed cleavage. In general, a peptide false discovery rate of <1% was accepted as the standard filtering criterion for identification. Spectral count analysis using Scaffold version 4.3.4 was used to evaluate abundance of proteins in Regeneration Wash, PBS wash and total cell lysate. The predominant molecular functions and biological processes were mapped for differentially expressed proteins to GO terms using BiNGO v2.44 coupled to the visualization potential of Cytoscape v3.1.1. GO annotation p-values were obtained by a hypergeometric statistical test (cluster versus the whole annotation as reference set) where the Benjamini-Hochberg False Discovery Rate (FDR) included in the BiNGO software was used as the correction tool. The GO database was current as of August 28th, 2014.

## Results and Discussion

### Synthesis of the AdoR-directed Probe

Although preparations of 8-azido or *N* ^6^-biotinylated AdoR probes have been reported in the past, to the best of our knowledge probe **1** is the first example of an AdoR probe containing an 8-azido group and a *N* ^6^-biotinylated purine moiety. The developed synthesis taking advantage of the previously reported 6,8-dichloro intermediate **4** to selectively modify the purine ring first in 6-position with a linker, and subsequently, in 8-position with the azido group has provided a synthetically tractable strategy. In addition, our synthetic strategy is operationally simple for half-gram scale preparations. Furthermore, the amide coupling chemistry to introduce biotin followed by the selective deprotection of the 5′-hydroxy-group reduced the number of challenging purification steps. Importantly, probe **1** has been designed with the idea to utilize its free 5′-hyrdoxy group as a chemical handle for the convenient preparation of any putative 5′-substituted AdoR probes to study targeted AdoR-dependent processes in entire proteomes.

### Probe 1 Labeling of cADA

**Fig 4:**
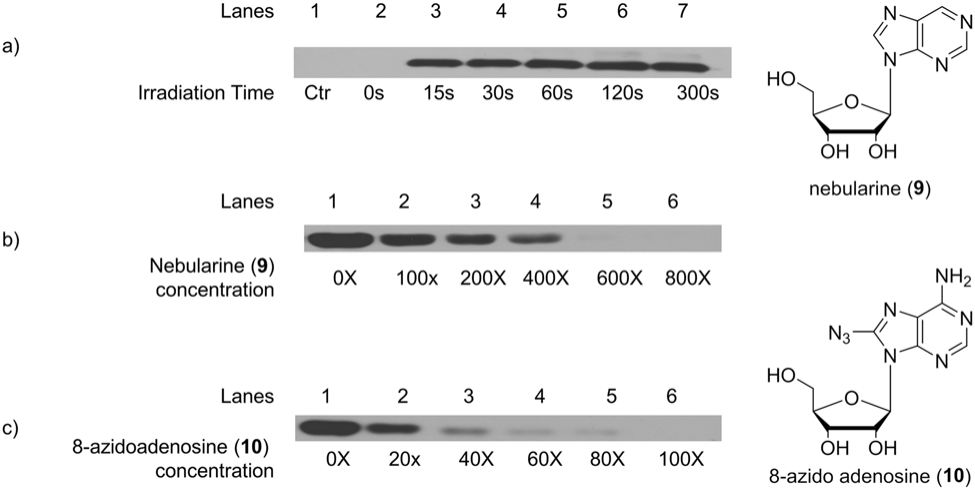
Effect of Irradiation Time and Competitive Inhibitors: Adenosine deaminase (cADA) was subjected to UV exposure in the presence and absence of known competitive inhibitors of cADA. a) Effect of irradiation time on the labeling cADA with the probe **1**: lane 1 represents control cADA without any probe (ctr), and lanes 2–7 represent cADA irradiated with UV light for 0 to 300 seconds. b) Inhibition of cADA labeling by nebularine **9**: lanes 1–6 show the decrease in cADA labeling by the probe **1** with an increase in the concentration of nebularine **9**, from molar ratios of [nebularine]/[cADA] of 0 to 800. c) Inhibition of cADA labeling by 8-azido adenosine **10**: lanes 1–6 shows the decrease in cADA labeling by the probe **1** with an increase in the concentration of 8-azidoadenosine **10**, from molar ratios of [8-azidoadenosine]/[cADA] of 0 to 100.

First, probe **1** was used to label cADA, a commercially available AdoR-binding protein of high quality. The goal of these experiments was not to fully investigate the labeling of cADA with probe **1**, but to establish the optimum parameters for (*a*) the use of probe **1** to label AdoR-binding proteins within a complex proteome and (*b*) the subsequent identification of the probe **1**-labeled proteins with Western analysis using an anti-biotin antibody. cADA was treated with the AdoR probe under UV light at a wavelength greater than 295 nm [28]. The probe **1**-labeled cADA was evaluated by SDS-PAGE followed by Western analysis using an anti-biotin HRP conjugate antibody. Experimental variables such as UV irradiation time, ratio of [probe **1**]/[cADA], amount of antibody used per SDS-gel, and antibody incubation times were optimized for the best use of probe **1**. Western analysis showed no labeling of cADA with the AdoR probe in the absence of UV irradiation (Fig. 4a). At a constant concentration of cADA, the amount of the labeled cADA seen by Western analysis increased up to a UV light exposure time of 2 minutes. Irradiation beyond 2 minutes did not increase the amounts of cADA labeled with the AdoR probe (Fig. 4a). Therefore, we standardized the UV irradiation time to 2 minutes. To further investigate the binding of probe **1** to cADA, we determined if two adenosine analogs, nebularine **9** (purine ribonucleoside) and 8-azidoadenosine **10**, will protect cADA against probe **1** labeling. As shown in Fig. 4b, nebularine **9** decreased the labeling of cADA by probe **1** in a concentration-dependent manner. Similar results were obtained with 8-azidoadenosine **10** (Fig. 4c). The inhibition of probe **1** labeling by both nebularine **9** and 8-azidoadenosine **10** suggests that probe **1** is binding at a nucleoside binding site in cADA, most likely the active site. The outcome of these first set of experiments were encouraging as we optimized *(a)* the conditions for the labeling of cADA with probe **1**; *(b)* the visualization of probe **1**-labeled cADA via Western analysis; and *(c)* the protection experiments using two different AdoR analogs, nebularine **9** and 8-azidoadenosine **10**, suggesting that probe **1** is binding to cADA at a nucleoside-binding site.

### Proteomic Labeling with Probe 1

**Fig 5:**
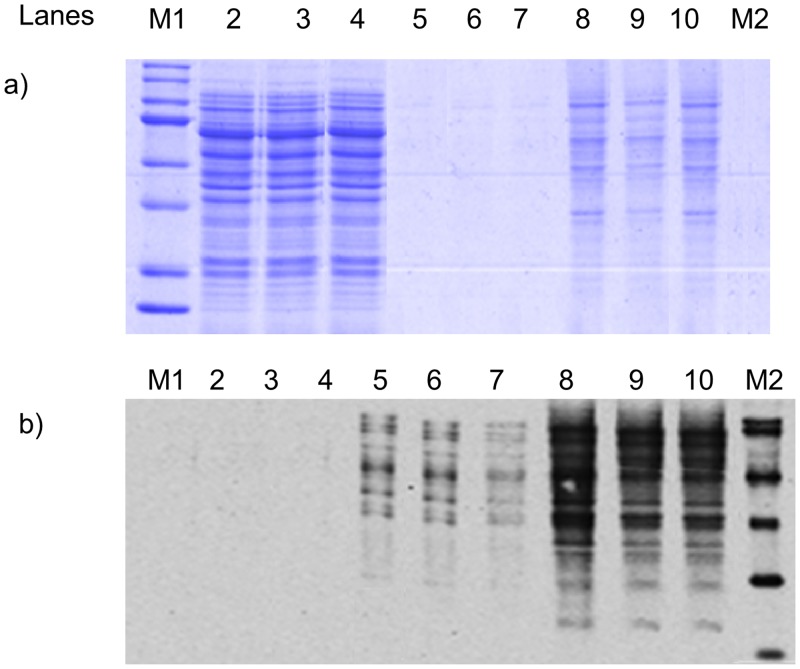
Western Analysis representing reproducible enrichment of probe 1-labeled proteome by avidin chromatography. Duplicate SDS-gels containing different washes from the avidin column loaded with N_18_TG_2_ were run where Lane 1 contains MW standards (M1), lanes 2–4 are the PBS Wash, lanes 5–7 are the Biotin Wash, lane 8–10 are the Regeneration Wash, and lane 11 contains biotinylated MW standards (M2). a) gel stained using Coomassie total protein stain and b) gel visualized via Western analysis through an anti-biotin antibody HRP conjugate.

Given our initial successes with probe **1**, we initiated proteomic profiling experiments using mouse neuroblastoma N_18_TG_2_ cells. These cells are routinely cultured in our laboratory for other projects [29]. First, endogenous avidin-binding proteins were removed from the soluble N_18_TG_2_ cell proteome (prepared from 5–6 × 10^7^ confluent N_18_TG_2_ cells) to eliminate false negatives. Following removal of the endogenous avidin-binding proteins, the soluble N_18_TG_2_ cell proteome was mixed with probe **1** in the dark and the soluble proteome-probe mixture was exposed to UV light (2 min with wavelength higher than 295 nm [28]) and immediately diluted with cold PBS. Our optimization experiments with the soluble N_18_TG_2_ cell proteome demonstrated that a [probe **1**]/[total protein] ratio of 10 yielded an acceptable level of labeling as evaluated by Western analysis using an anti-biotin HRP conjugate antibody. This experiment yielded a relatively large number of biotinylated proteins, indicating significant labeling with the probe **1** (Fig. 5). The majority of proteins within the soluble N_18_TG_2_ cell proteome, which do not bind probe **1**, were eluted in the flow through with PBS, and are shown as the PBS Wash samples in Fig. 5a (lanes 2–4). Evidence supporting this statement comes from a comparison of the Coomassie stain to the Western analysis of the PBS Wash: a number of proteins were prominently stained in the PBS Wash with Coomassie Blue (Fig. 5a, lanes 2–4), yet nothing was seen in the Western analysis of the PBS Wash (Fig. 5b, lanes 2–4). The probe **1**•protein conjugates that were relatively weakly bound to the avidin column were eluted with the Biotin Wash. The SDS-PAGE lanes representing the Biotin Wash were not detectable upon Coomassie staining (Fig. 5a, lanes 5–7), but the relatively small number of probe **1**•protein conjugates present in the Biotin Wash were observed in the Western analysis (Fig. 5b, lanes 5–7). The probe **1**•protein conjugates that were most tightly bound to the avidin column were present in the Regeneration Wash and visible when stained with both Coomassie Blue (Fig. 5a, lanes 8–10) and with the anti-biotin HRP conjugate (Fig. 5b, lanes 8–10). These data from the analysis of the Regeneration Wash show that the number of proteins in this fraction was greater than the total from the Biotin Wash, but less than total in the PBS Wash. Measurements of total protein from the three fractions, PBS Wash + Biotin Wash + Regeneration Wash, indicate that less than 1% of the total protein is found in the Biotin Wash and approximately 10% of the total protein is found in the Regeneration Wash—in reasonable agreement with our estimate that ~25% of all known proteins bind an AdoR. Note that the data for three separate experiments are included in Fig. 5 demonstrating that the proteomic profiles are reproducible; yielding the same banding pattern from individual soluble proteomes prepared from the N_18_TG_2_ cells.

**Fig 6:**
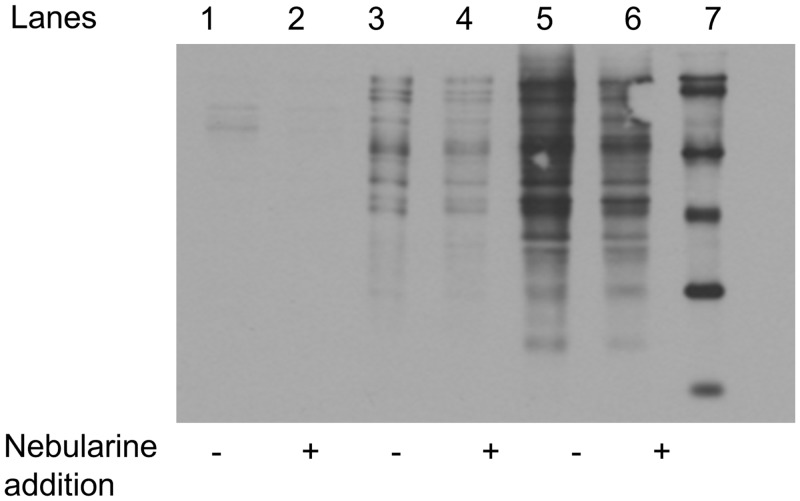
Effect of Nebularine on the Proteomic Profile from the Soluble N18TG2 Cell Proteome. Lanes 2–4 are the PBS Wash, lanes 3–4 are the Biotin Wash, lanes 5–6 are the Regeneration Wash, and lane 7 is biotinylated MW standards. The minus sign (-) indicates absence of added nebularine **9**, while the plus sign (+) indicates addition of nebularine **9** prior to addition of probe **1** ([nebularine **9**]/[probe **1**] = 10).

In order to assess probe specificity, the addition of nebularine **9** to the probe **1**-proteome mixture before UV exposure inhibits the formation of the probe **1**-labeled proteins, as seen in the Western analysis (Fig. 6). A 2–3 fold decrease in total labeling was observed for both the Biotin Wash and Regeneration Wash fractions when the [nebularine **9**]/[probe **1**] ratio was 10. The extent of nebularine inhibition differs from protein-to-protein and may provide an estimate for the dynamic range for probe **1** labeling of a particular protein (Fig. 6).

**Fig 7:**
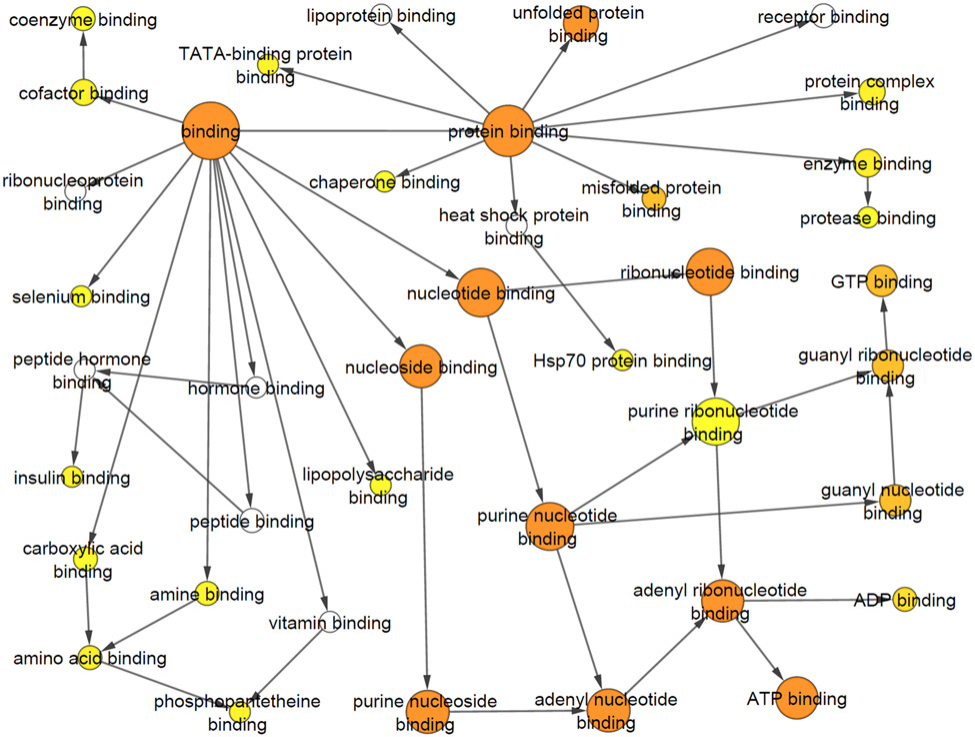
Gene Ontology (GO) molecular function categories observed using BiNGO Analysis for the Regeneration Wash. The nodes represent various molecular functions identified by this analysis where color represents significance of enrichment (orange, yellow, white represent order of significance). The arrows represent association of the broad functional categories (larger circles) and related subcategories (smaller circles).

**Fig 8:**
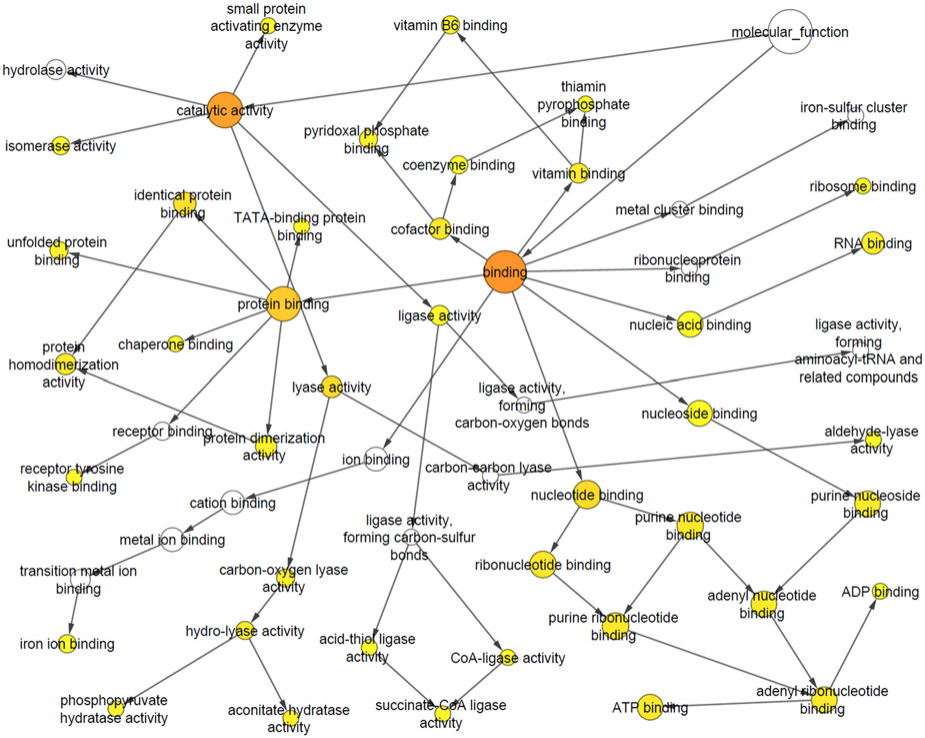
Gene Ontology (GO) molecular function categories observed using BiNGO Analysis for the PBS Wash. The nodes represent various molecular functions identified by this analysis where color represents significance of enrichment (orange, yellow, white represent order of significance). The arrows represent association of the broad functional categories (larger circles) and related subcategories (smaller circles).

These first experiments to profile the soluble N_18_TG_2_ cell proteome with probe **1** indicated an acceptable level of protein labeling, alleviating our concern that the family of probe **1**•labeled AdoR binding proteins would be too large and, thus, overwhelm our post-labeling strategies to further characterize the probe **1**•labeled proteins by mass spectrometry. In this regard, the isolated proteins from both labeled and non-labeled fractions were identified by tandem mass spectrometry analysis of trypsin-digested proteins extracted from the entire lane of the SDS-PAGE gel. Mass spectrometry-based proteomic analysis of the total cell lysate, PBS Wash (similar to lanes 2–4 in Fig. 5a) and Regeneration Wash (similar to lanes 8–10 in Fig. 5a) revealed a panel of 757 unique proteins (a total of 266 proteins detected in PBS Wash, 393 proteins detected in Regeneration Wash and 460 proteins detected in total cell lysate.). The abundance of proteins in a particular category of samples was arranged by spectral counts (mass spectrometric measurement of relative protein abundance) in decreasing order for the total cell lysate, PBS Wash and Regeneration Wash fractions. Out of the top 50 hits identified in the Regeneration Wash, 44 showed nucleic acid-binding functional classification. These include 19 ATP-binding, 10 GTP-binding, 4 NAD/NADP-binding, 9 RNA/ poly(A)RNA-binding, 1 FAD-binding and 1 nucleotide binding/ poly(U) RNA-binding (S1 Table) proteins based on Gene Ontology (GO) classification. Examples of ATP- and GTP-binding proteins over-represented in the Regeneration Wash include elongation factors, tubulins, heat shock proteins and T-complex proteins. Assessment of the statistical overrepresentation of specific GO molecular function categories was performed using BiNGO, an open source bioinformatics tool that is used to analyze complex biological datasets through identification of functional categories and integrated molecular interaction networks. Tables 1–3 represent the top 10 statistically over-represented GO molecular function terms of the top 50 protein hits in Regeneration Wash, PBS Wash and overall proteins. Seven out of the ten GO categories determined for proteins identified in the Regeneration Wash are functionally relevant (based on nucleotide binding or equivalent property) to the putative ADoR-binding proteome in our study whereas only one out of the top-ten GO categories observed in the PBS Wash belongs to a functionally relevant category. The cluster frequency of the GO categories observed in the Regeneration Wash are also significantly higher than in PBS wash or in the overall proteome profile of the entire cell lysate without enrichment using the probe. Figs. 7 and 8 show the differences of GO categories observed for proteins from the Regeneration Wash fraction compared to the PBS Wash fraction. For completeness, the entire list of identified proteins from the proteomic analysis is provided in S2 Table and the complete list of proteins identified by BiNGO analysis is provided in S3–S5 Tables of supporting information.

**Table 1:** Top 10 statistically over-represented GO Biological Process terms in Regeneration Wash, according to BiNGO.

GO-ID	P-value	corrected P-value	Description	Cluster frequency	Total Frequency
17076	7.74E-18	8.04E-16	purine nucleotide binding	56.1%	6.1%
166	9.46E-18	8.04E-16	nucleotide binding	58.5%	7.1%
32555	6.05E-17	2.60E-15	purine ribonucleotide binding	53.7%	5.9%
32553	6.12E-17	2.60E-15	ribonucleotide binding	53.7%	5.9%
51082	1.25E-16	4.24E-15	unfolded protein binding	22.0%	0.2%
5488	2.42E-14	6.85E-13	binding	92.7%	35.5%
30554	4.45E-12	9.49E-11	adenyl nucleotide binding	41.5%	5.1%
5515	4.47E-12	9.49E-11	protein binding	68.3%	18.6%
1883	5.57E-12	1.01E-10	purine nucleoside binding	41.5%	5.2%
1882	5.93E-12	1.01E-10	nucleoside binding	41.5%	5.2%

**Table 2:** Top 10 statistically over-represented GO Biological Process terms in PBS Wash, according to BiNGO.

GO-ID	P-value	corrected P-value	Description	Cluster frequency	Total Frequency
5488	2.74E-12	4.77E-10	binding	89.7%	35.5%
3824	2.25E-08	1.96E-06	catalytic activity	56.4%	16.8%
30060	1.77E-06	1.02E-04	L-malate dehydrogenase activity	5.1%	0.0%
5515	4.14E-06	1.80E-04	protein binding	51.3%	18.6%
46912	1.76E-05	6.13E-04	transferase activity, transferring acyl groups, acyl groups converted into alkyl on transfer	5.1%	0.0%
16615	3.69E-05	9.51E-04	malate dehydrogenase activity	5.1%	0.0%
16740	4.34E-05	9.51E-04	transferase activity	25.6%	5.6%
16829	4.81E-05	9.51E-04	lyase activity	10.3%	0.5%
4372	4.92E-05	9.51E-04	glycine hydroxymethyltransferase activity	5.1%	0.0%
166	5.53E-05	9.62E-04	nucleotide binding	28.2%	7.1%

**Table 3:** Top 10 statistically over-represented GO Biological Process terms in Overall Identified Proteins, according to BiNGO.

GO-ID	P-value	corrected P-value	Description	Cluster frequency	Total Frequency
5488	4.26E-88	3.07E-85	binding	77.1%	35.6%
166	1.26E-53	4.53E-51	nucleotide binding	28.7%	7.1%
3824	2.10E-41	5.06E-39	catalytic activity	41.0%	16.8%
5515	1.48E-40	2.66E-38	protein binding	43.3%	18.6%
17076	8.63E-40	1.24E-37	purine nucleotide binding	23.3%	6.1%
32553	3.76E-38	4.52E-36	ribonucleotide binding	22.4%	5.9%
32555	1.73E-37	1.79E-35	purine ribonucleotide binding	22.2%	5.9%
3735	2.84E-34	2.56E-32	structural constituent of ribosome	6.5%	0.4%
5198	3.32E-27	2.66E-25	structural molecule activity	8.9%	1.2%
8135	4.26E-26	3.07E-24	translation factor activity, nucleic acid binding	5.2%	0.3%

Quantitation validation by Western analysis of selected proteins identified by LC-MS/MS from the Regeneration Wash (labeled) and the PBS Wash (non-labeled) yielded comparable results. For example, Western analysis of fructose bisphosphate aldolase A detected this protein in all three PBS Wash samples, but not in the Regeneration Wash samples as expected based on the LC-MS/MS data (S1a Fig.). Similarly, stress-induced-phosphoprotein 1 was detected in all three Regeneration Wash samples by Western analysis, but was not identified in the PBS Wash samples (S1b Fig.). Therefore, it was established that the AdoR-directed probe **1** can be used successfully to photolabel AdoR-binding proteins in a specific manner and that probe **1**•AdoR-binding conjugates can be enriched from complex protein mixtures (e.g., cell lysates) and quantified by mass spectrometry.

Further analysis of the top 150 proteins isolated by our enrichment approach using probe **1** showed that 111 out of 150 (74%) were nucleotide-binding proteins as compared to 82 out of 132 (62%) in an FSBA probe-based isolation approach reported in Jurkat cells [16]. Among the nucleotide-binding proteins identified by probe **1** in the Regeneration Wash fraction, 15 protein groups (a group condenses the protein identifications by combining multiple isoforms of the same protein within a particular group) were among the protein groups isolated by the FSBA approach in addition to 32 possibly unique nucleotide-binding protein groups in our dataset. A few examples of the protein groups that were only identified in our approach and are functionally annotated as nucleotide-binding proteins are ADP-ribosylation factor 3, tRNA ligases (alanine—tRNA ligase, cytoplasmic, arginine—tRNA ligase, cytoplasmic, and glycine—tRNA ligase; diadenosine), caprin-1, D-3-phosphoglycerate dehydrogenase; 3-PGDH, electron transfer flavoprotein subunit alpha, far upstream element-binding protein 1, glucosidase 2 subunit beta, histone H1.4, histone H2A type 1, lon protease homolog, poly(rC)-binding protein 1; alpha-CP1, trifunctional purine biosynthetic protein and V-type proton ATPase subunit B. Although it is important to consider that the proteome profiles are different between the two cell types when performing this comparison, several proteins identified after enrichment are from central processes such as energy metabolism and protein translation, and therefore should be represented in both cell types. For example, glyceraldehyde 3-phosphate dehydrogenase was identified in the Regeneration Wash using probe **1** and identified in the FSBA approach. Interestingly, this protein has been shown to be completely inactivated by a reactive ATP analog, N6-(6-bromoacetamidohexyl)-AMP•PCP [17] and thus provides additional evidence to the specificity or our probe for nucleoside/nucleotide binding. Alternatively, histones and tRNA ligases, for example, are expected to be in both cell types; however, these proteins appear to be unique to the dataset obtained by probe **1**. Overall, our results suggest that the probe used in the reported study can provide complementary data when compared to other probe designs with a reactive group in the 5′-position which specifically investigate proteins binding to only particular 5′-substituted AdoRs of the extended AdoR class. The reported methodology enables an efficient and scalable pipeline to generate reproducible protein expression profiles that could be used with other methods with the ultimate goal to enhance coverage of the nucleoside and nucleotide-binding sub-proteome.

## Supporting Information

S1 Document Experimental procedures for the preparation and characterization of probe 1. (DOCX)

S1 Fig Validation of proteins identified in PBS and Regeneration wash using Western Blot Analysis- Results are as expected based on the LC-MS/MS data. a) detection of bands for fructose bisphosphate aldolase A in all PBS Wash samples but not in the Regeneration Wash samples b) detection of bands for stress-induced-phosphoprotein 1 in all Regeneration Wash samples but not in the PBS Wash samples.
(TIF)

S1 Table GO analysis (by Scaffold) of the top 50 (as per average mass spectral counts) identified proteins in Regeneration Wash. (DOCX)

S2 Table GO analysis (by Scaffold) of the complete list of proteins identified from PBS Wash, Regeneration Wash and total cell lysate (control) of N_18_TG_2_ cells. (DOCX)

S3 Table Complete list of statistically over-represented GO Biological Process terms in Regeneration Wash, according to BiNGO. (DOCX)

S4 Table Complete list of statistically over-represented GO Biological Process terms in PBS Wash, according to BiNGO. (DOCX)

S5 Table Complete list of statistically over-represented GO Biological Process terms in Overall Identified Proteins, according to BiNGO. (DOCX)
