# Supplementary material for: Synthesis and Evaluation of a Novel Adenosine-Ribose Probe for Global-Scale Profiling of Nucleoside and Nucleotide-Binding Proteins
Source: PLoS One. 2015 Feb 11;10(2):e0115644. doi: 10.1371/journal.pone.0115644 (PMC4324776; doi:10.1371/journal.pone.0115644)
Supplement: S1 Document — (DOCX) [file pone.0115644.s001.docx]

**SUPPORTING INFORMATION**

**Synthesis and Evaluation of a Novel Adenosine-Ribose Probe for Global-Scale Profiling of Nucleoside and Nucleotide-Binding Proteins**

Shikha Mahajan^1^, Roman Manetsch^1^, David J. Merkler^1^, Stanley M. Stevens Jr.^2^

^1^Department of Chemistry, University of South Florida, 4202 E. Fowler Ave., Tampa, FL, 33620

^2^Department of Cell Biology, Microbiology and Molecular Biology, University of South Florida, 4202 E. Fowler Ave., Tampa, FL, 33620

**Probe Synthesis**

**General:** All reagents and solvents were purchased from commercial sources and used without further purification. All reactions were performed under an argon atmosphere unless otherwise indicated. Prior to the use of solvents in reactions, they were purified by passing the degassed solvents through a column of activated alumina and transferred by an oven-dried syringe. Thin layer chromatography was performed on Merck TLC plates (silica gel 60 F_254_). Spots were visualized under UV (254 or 356 nm) light. Preparative column chromatography was performed on silica gel (230-400 mesh) suspended in CH_2_Cl_2_. ^1^H NMR and ^13^C NMR were recorded on a Varian Inova 400 (400 MHz) or a Bruker Avance DPX-250 (250 MHz) instrument. The purification of designated compounds was carried out using reversed-phase HPLC (Agilent column Eclipse XDB-C_18_, 5 µm, 9.4 mm x 250 mm) on a Waters Prep LC 4000 system with a 996 photo-diode array detector. Compounds were eluted using a gradient elution of A:B (95:5 to 0:100) over 40 min at a flow rate of 5.0 mL/min, where solvent A was H_2_O (0.05% TFA) and solvent B was CH_3_CN (0.05% TFA). The high-resolution MS data were measured on an Agilent 6210 Series MSD/TOF with electrospray ionization. The LC/MS data were measured on an Agilent 1100 LC/MSD-VL with electrospray ionization. Compounds **3** and **4** were synthesized as previously reported.^[[1]](#footnote-1)^

**2',3',5'-Tri-*O*-acetyl-8-chloro-6-(*N*^6^-*tert*-butoxycarboyl-6-amino-1-hexylamino)-purine riboside (5):** To a solution of **4** (500 mg, 1.12 mmol) in dry DCM (5 mL) at -10^o^C, anhydrous DIPEA (157 µL, 1.12 mmol) and mono-*N-*Boc-1,6-hexandiamine (266 mg, 1.23 mmol) was added. The solution was stirred for 3 hours and then allowed to stir at room temperature overnight. The reaction mixture was washed with water and the product was extracted with DCM. The DCM extract was dried over anhydrous sodium sulfate, concentrated to a pale yellow oil and subjected to reverse phase HPLC column chromatography (30-100% acetonitrile in H_2_O) and subjected to freeze drying to give **5** (463 mg, 66 %) as white solid. R_f_ = 0.55 (CH_2_Cl_2_/ CH_3_OH, 95/5, v/v). ^1^H NMR (400 MHz, CDCl_3_) δ 8.40 (s, 1H), 6.05 (m, 1H), 5.90 (m, 1H), 5.73 (s, 1H), 5.52 (t, 1H), 4.74 (s, 1H), 4.37 (m, 1H), 4.29 (m, 2H), 3.47 (m, 1H), 3.43 (m, 1H), 2.99 (m, 2H), 2.02 (s, 3H), 1.94 (s, 3H), 1.93 (s, 3H), 1.56 (m, 2H), 1.39-1.27 (m, 15H). HRMS (ESI-TOF) m/z for [M+H]^+^ calcd for C_27_H_40_ClN_6_O_9_ 627.2545; found 627.2547.

**2',3',5'-Tri-*O*-acetyl-8-azido-6-(*N*^6^-*tert*-butoxycarboyl-6-amino-1-hexylamino)-purine riboside (6):** To a solution of **5** (463 mg, 0.73 mmol) in dioxane (10 mL), CsN_3_ (647 mg, 3.65 mmol) and trimethylsilyl azide (480 µL, 3.65 mmol) was added. The solution was refluxed for 3 days. The reaction was followed every 24 hours by LC-MS until the peak of the chloro derivative **5** completely disappeared providing the desired product **6**. The reaction mixture was washed with water and the product was extracted with DCM (3 × 25mL). The DCM extract was dried over anhydrous sodium sulfate, concentrated to a pale yellow solid and subjected to flash column chromatography (0-3% MeOH in DCM) to give **6** (369 mg, 79 %) as white solid. R_f_ = 0.55 (CH_2_Cl_2_/ CH_3_OH, 95/5, v/v). ^1^H NMR (400 MHz, CDCl_3_) δ 8.35 (s, 1H), 6.13 (m, 1H), 5.88 (m, 1H), 5.52 (bd, 2H), 4.58 (s, 1H), 4.46 (bd, 1H), 4.32 (m, 2H), 3.54 (m, 1H), 3.44 (m, 1H), 3.07 (m, 2H), 2.10 (s, 3H), 2.02 (s, 3H), 1.99 (s, 3H), 1.64 (m, 2H), 1.47 – 1.35 (m, 15H).

**2',3',5'-Tri-*O*-acetyl-8-azido-6-(6-amino-hexylamino)-purine riboside (7):** A solution of **6** (369 mg, 0.58 mmol) in 9:1 dry DCM:TFA (3.7 mL) was stirred at room temperature for 4 hours. The reaction was followed by LC-MS for complete deprotection of *N-*Boc group and formation of the desired product. The reaction was quenched with triethyl amine (2 mL) and extracted with DCM (3 × 15 mL). The DCM extract was dried over anhydrous sodium sulfate and concentrated to pale yellow solid **7** (251 mg, 81%). R_f_ = 0.45 (CH_2_Cl_2_/CH_3_OH, 90/10, v/v). Attempts to purify product using flash column chromatography resulted in a significant loss of product and hence crude product was directly subjected to the next step without further purification.

**2',3',5'-Tri-*O*-acetyl-8-azido-6-(*N*^6^-biotinyl-6-amino-1-hexylamino)-purine riboside (8):** To a solution of biotin (115 mg, 0.47 mmol), EDCI·HCl (90 mg, 0.47 mmol), and DMAP (86 mg, 0.70 mmol) in dry DMF (5 mL), a solution of crude **7** (251 mg, 0.47 mmol) in dry DCM (5 mL) was slowly added; the solution was stirred at room temperature for 8 hours. The reaction was washed with water (2 × 10 mL) and brine solution (1 × 10 mL) and the product was extracted with DCM. The DCM extract was dried over anhydrous sodium sulfate and concentrated to a pale yellow solid and subjected to reversed-phase column chromatography (30-100% acetonitrile in H_2_O) to give **8** (256 mg, 72%) after freeze-drying. R_f_ = 0.3 (CH_2_Cl_2_/CH_3_OH, 90/10, v/v). ^1^H NMR (400 MHz, CDCl_3_) δ 8.37 (s, 1H), 6.3 (bs, 1H), 6.28 (m, 1H), 6.17 (d, 1H), 5.95 (t, 1H), 5.62 (m, 1H), 4.54 – 4.46 (m, 2H), 4.41 (d, 1H), 4.37 (m, 2H), 4.30 (m, 1H), 3.56 – 3.46(m, 2H), 3.20 (m, 2H), 3.12 (m, 1H), 2.87 (m, 1H), 2.69 (m, 1H), 2.20 (m, 2H), 2.11 (s, 3H), 2.02 (s, 6H), 1.75 – 1.61 (m, 6H), 1.44 (m, 2 H), 1.41-1.25 (m, 6H). HRMS (ESI-TOF) m/z for [M+H]^+^ calcd for C_32_H_46_N_11_O_9_S 760.3195; found 760.3186.

**2',3'-Di-*O*-acetyl-8-azido-6-(*N*^6^-biotinyl-6-amino-1-hexylamino)-purine riboside (1):** To a solution of **8** (100 mg, 0.13 mmol) in MeOH (5 mL), ammonia solution was slowly added and stirred at -40˚C for 2 hours. The reaction mixture was diluted with MeOH and concentrated immediately under vacuum to give 73 mg of a foamy solid which was purified by HPLC chromatography (20-80% acetonitrile in H_2_O) followed by freeze-drying to give **1** (57 mg, 61%) along with small amount of the monoacetyl derivative. R_f_ = 0.25 (CH_2_Cl_2_/CH_3_OH, 90/10, v/v). ^1^H NMR (400 MHz, CDCl_3_) δ 8.43 (s, 1H), 6.78 (d, 1H), 6.57 (s, 2H), 6.45 (d, 1H), 5.67 – 5.58 (m, 1H), 5.51 (d, 1H), 4.49 (s, 1H), 4.28(s, 2H), 3.89 (dd, 2H), 3.51 (d, 2H), 3.10 (s, 3H), 2.76 (m, 2H), 2.18 (s, 2H), 2.11 (s, 6H), 1.61 (s, 6H), 1.46 – 1.21 (m, 8H). HRMS (ESI-TOF) m/z for [M+H]^+^ calcd for C_30_H_44_N_11_O_8_S 718.3090; found 718.3082.

1. a) [Roelen](http://pubs.acs.org/action/doSearch?action=search&author=Roelen%2C+H&qsSearchArea=author) H, [Veldman](http://pubs.acs.org/action/doSearch?action=search&author=Veldman%2C+N&qsSearchArea=author) N, [Spek](http://pubs.acs.org/action/doSearch?action=search&author=Spek%2C+A+L&qsSearchArea=author) AL , [von Frijtag Drabbe Künzel](http://pubs.acs.org/action/doSearch?action=search&author=von+Frijtag+Drabbe+K%C3%BCnzel%2C+J&qsSearchArea=author) J, [Mathôt](http://pubs.acs.org/action/doSearch?action=search&author=Math%C3%B4t%2C+R+A+A&qsSearchArea=author) RAA, [IJzerman](http://pubs.acs.org/action/doSearch?action=search&author=IJzerman%2C+A+P&qsSearchArea=author) AP (1996) *N*^6^,C8-Disubstituted Adenosine Derivatives as Partial Agonists for Adenosine A_1_ Receptors. J Med Chem, 39 (7), 1463-1471; b) Szekeres GL, Robins RK, Boswell KH, Long RA, (1975) Synthesis of 8-Amino-9-β-D ribofuranosylpurin-6-thione and Related 6-Substituted 8-Aminopurine Nucleosides. J Heterocycl Chem, 12, 15-19; c) Gersterb JF, Hinshaw BC, Roabins RK, Townsend LB (1968) Purine Nucleosides. XIX. The Synthesis of Certain 8-Chloropurine Nucleosides and Related Derivatives. 33 (3), 1070-1073. [↑](#footnote-ref-1)
