# Supplementary figures and images for: Synthesis and Evaluation of a Novel Adenosine-Ribose Probe for Global-Scale Profiling of Nucleoside and Nucleotide-Binding Proteins
Source: PLoS One. 2015 Feb 11;10(2):e0115644. doi: 10.1371/journal.pone.0115644 (PMC4324776; doi:10.1371/journal.pone.0115644)

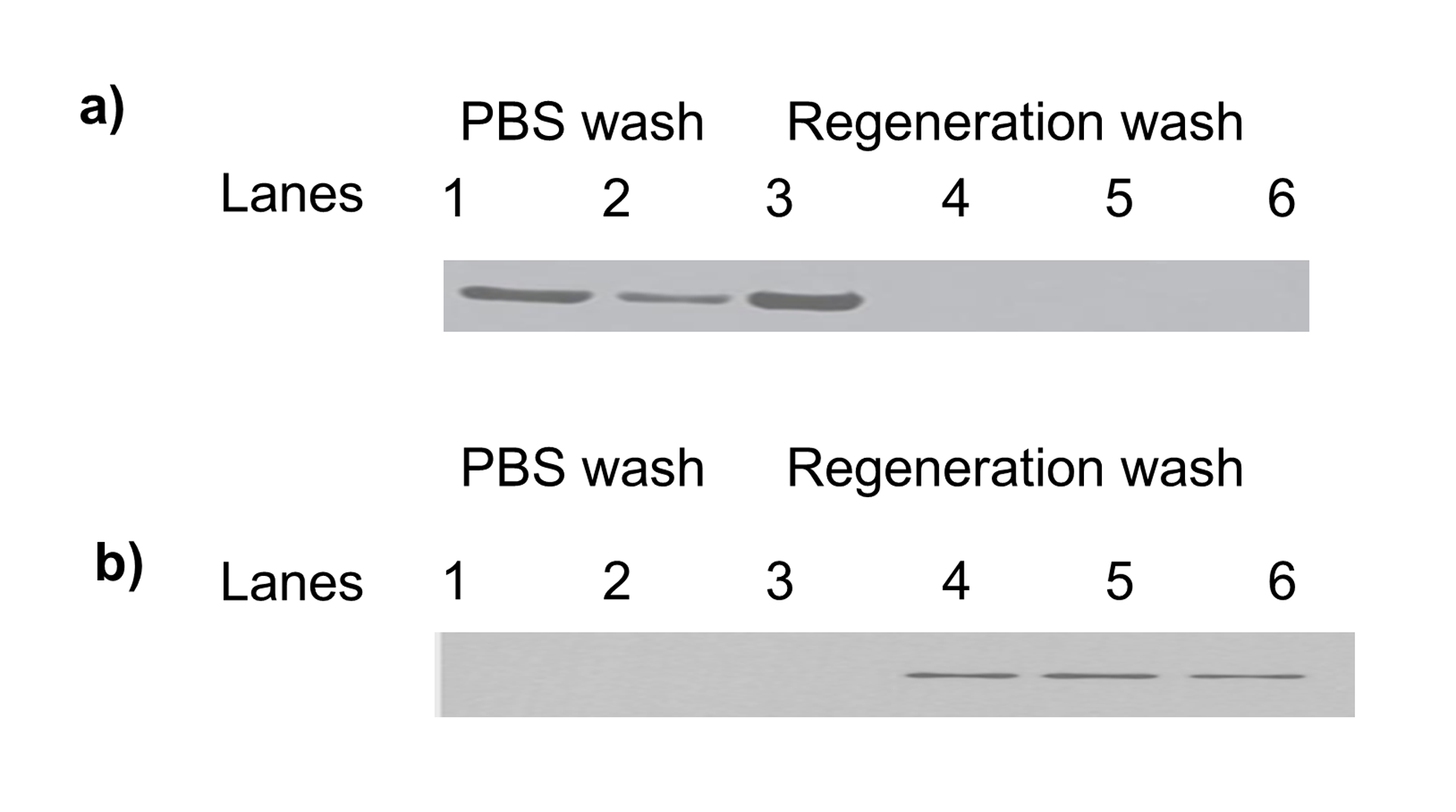

Supplement: S1 Fig — a) detection of bands for fructose bisphosphate aldolase A in all PBS Wash samples but not in the Regeneration Wash samples b) detection of bands for stress-induced-phosphoprotein 1 in all Regeneration Wash samples but not in the PBS Wash samples. (TIF) [file pone.0115644.s002.tif]
