# Supplementary material for: Synthesis and Evaluation of a Novel Adenosine-Ribose Probe for Global-Scale Profiling of Nucleoside and Nucleotide-Binding Proteins
Source: PLoS One. 2015 Feb 11;10(2):e0115644. doi: 10.1371/journal.pone.0115644 (PMC4324776; doi:10.1371/journal.pone.0115644)
Supplement: S1 Table — (DOCX) [file pone.0115644.s003.docx]

**Table S1** – GO analysis (by Scaffold) of the top 50 (as per average spectral count ) identified proteins in Regeneration Wash.

| Identified Proteins (757) | Accession Number | binding | Average Spectral Count | | |
| --- | --- | --- | --- | --- | --- |
|  |  |  | PBS | Regen | Control |
| Elongation factor 1-alpha 1; EF-1-alpha-1; | P10126\|EF1A1 | GTP binding | 11 | 68 | 80 |
| ATP synthase subunit beta, mitochondrial; Flags: | P56480\|ATPB | ATP binding | 20 | 64 | 58 |
| Tubulin beta-5 chain. | P99024\|TBB5 | GTP binding | 9 | 59 | 78 |
| Tubulin alpha-1B chain; Alpha-tubulin | P05213\|TBA1B | GTP binding | 0 | 62 | 97 |
| Tubulin alpha-1C chain; Alpha-tubulin | P68373\|TBA1C | GTP binding | 0 | 60 | 70 |
| Tubulin alpha-1A chain; Alpha-tubulin | P68369\|TBA1A | GTP binding | 13 | 60 | 97 |
| Tubulin beta-4B chain; Tubulin beta-2C | P68372\|TBB4B | GTP binding | 6 | 51 | 67 |
| Actin, cytoplasmic 1; Beta-actin; | P60710\|ACTB | ATP binding | 31 | 46 | 197 |
| Tubulin beta-2A chain OS=Mus musculus GN=Tubb2a PE=1 SV=1 | Q7TMM9\|TBB2A | GTP binding | 0 | 16 | 61 |
| Heat shock cognate 71 kDa protein; Heat | P63017\|HSP7C | ATP binding | 21 | 48 | 102 |
| Elongation factor 1-alpha 2 OS=Mus musculus GN=Eef1a2 PE=1 SV=1 | P62631\|EF1A2 | GTP binding | 0 | 33 | 21 |
| Tubulin beta-3 chain. | Q9ERD7\|TBB3 | GTP binding | 0 | 37 | 49 |
| ATP synthase subunit alpha, mitochondrial; Flags: | Q03265\|ATPA | ATP binding | 8 | 34 | 19 |
| Actin, alpha cardiac muscle 1; AltName: | P68033\|ACTC | ATP binding | 10 | 27 | 41 |
| Heat shock protein HSP 90-alpha; Heat | P07901\|HS90A | ATP binding | 38 | 31 | 78 |
| Elongation factor 2; EF-2. | P58252\|EF2 | GTP binding | 15 | 28 | 80 |
| Endoplasmin; 94 kDa glucose-regulated | P08113\|ENPL | ATP binding | 9 | 22 | 30 |
| T-complex protein 1 subunit beta; TCP-1-beta; | P80314\|TCPB | ATP binding | 12 | 24 | 17 |
| Stress-70 protein, mitochondrial; 75 | Q7TSZ0\|Q7TSZ0 | ATP binding | 5 | 23 | 20 |
| D-3-phosphoglycerate dehydrogenase; 3-PGDH; | Q61753\|SERA | NAD binding | 0 | 23 | 13 |
| T-complex protein 1 subunit delta; TCP-1-delta; | P80315\|TCPD | ATP binding | 4 | 25 | 10 |
| T-complex protein 1 subunit epsilon; | P80316\|TCPE | ATP binding | 3 | 21 | 13 |
| T-complex protein 1 subunit gamma; TCP-1-gamma; | P80318\|TCPG | ATP binding | 7 | 20 | 13 |
| 60S ribosomal protein L12. | P35979\|RL12 | RNA binding | 3 | 19 | 10 |
| 40S ribosomal protein S3. | P62908\|RS3 | mRNA binding | 0 | 18 | 6 |
| T-complex protein 1 subunit theta; TCP-1-theta; | P42932\|TCPQ | ATP binding | 4 | 21 | 9 |
| T-complex protein 1 subunit alpha; TCP-1-alpha; | P11983\|TCPA | ATP binding | 8 | 19 | 9 |
| Glycine--tRNA ligase; Diadenosine | Q9CZD3\|SYG | ATP binding | 0 | 11 | 8 |
| Far upstream element-binding protein 2; | Q3U0V1\|FUBP2 | mRNA binding | 0 | 14 | 0 |
| Electron transfer flavoprotein subunit alpha, | Q99LC5\|ETFA | FAD binding | 0 | 11 | 5 |
| Heterogeneous nuclear ribonucleoprotein K; | P61979\|HNRPK | RNA binding | 1 | 16 | 11 |
| Polyadenylate-binding protein 1; PABP-1; | P29341\|PABP1 | *[poly(A) RNA binding/ poly(A) binding/ poly(U) RNA binding](http://www.ebi.ac.uk/QuickGO/GTerm?id=GO:0044822) | 0 | 13 | 3 |
| Glyceraldehyde-3-phosphate dehydrogenase OS=Mus musculus GN=Gm10566 PE=3 SV=1 | E9PZH9\|E9PZH9 | *[NAD binding / *NADP binding](http://www.ebi.ac.uk/QuickGO/GTerm?id=GO:0051287) | 0 | 15 | 22 |
| 40S ribosomal protein S19. | Q9CZX8\|RS19 | *[poly(A) RNA binding](http://www.ebi.ac.uk/QuickGO/GTerm?id=GO:0044822) | 0 | 17 | 6 |
| 78 kDa glucose-regulated protein; GRP-78; | P20029\|GRP78 | *[ATP-binding / Nucleotide-binding](http://www.uniprot.org/keywords/KW-0067) | 6 | 17 | 51 |
| Importin subunit beta-1; Karyopherin | P70168\|IMB1 | *[poly(A) RNA binding](http://www.ebi.ac.uk/QuickGO/GTerm?id=GO:0044822) | 0 | 12 | 7 |
| 60 kDa heat shock protein, mitochondrial; AltName: | P63038\|CH60 | *[ATP-binding / Nucleotide-binding](http://www.uniprot.org/keywords/KW-0067) | 6 | 66 | 35 |
| Heat shock protein HSP 90-beta; Heat | P11499\|HS90B | *[ATP-binding / Nucleotide-binding](http://www.uniprot.org/keywords/KW-0067) | 46 | 58 | 120 |
| Glyceraldehyde-3-phosphate dehydrogenase; | P16858\|G3P | *[NAD binding / NADP binding](http://www.ebi.ac.uk/QuickGO/GTerm?id=GO:0051287) | 13 | 54 | 159 |
| Pyruvate kinase PKM; Pyruvate kinase | P52480\|KPYM | *ATP binding | 25 | 32 | 57 |
| Fatty acid synthase; Includes: RecName: | P19096\|FAS | *[NAD binding / NADP binding/ poly(A) RNA binding](http://www.ebi.ac.uk/QuickGO/GTerm?id=GO:0044822) | 6 | 28 | 16 |
| Poly(rC)-binding protein 1; Alpha-CP1; | P60335\|PCBP1 | *[RNA binding/ poly(A) RNA binding](http://www.ebi.ac.uk/QuickGO/GTerm?id=GO:0003723) | 1 | 16 | 9 |
| Stress-induced-phosphoprotein 1; STI1; | Q60864\|STIP1 | *[poly(A) RNA binding](http://www.ebi.ac.uk/QuickGO/GTerm?id=GO:0044822) | 0 | 18 | 5 |
| 14-3-3 protein zeta/delta; Protein | P63101\|1433Z | *[poly(A) RNA binding](http://www.ebi.ac.uk/QuickGO/GTerm?id=GO:0044822) | 20 | 14 | 29 |
| Peroxiredoxin-1; Macrophage 23 kDa | P35700\|PRDX1 | protein binding | 2 | 29 | 9 |
| Dihydropyrimidinase-related protein 3; DRP-3; | Q62188\|DPYL3 | SH3 domain binding | 3 | 24 | 10 |
| ATP synthase subunit O, mitochondrial; AltName: | Q9DB20\|ATPO | steroid binding | 0 | 21 | 2 |
| Peroxiredoxin-2; Thiol-specific | Q61171\|PRDX2 | protein binding | 1 | 21 | 9 |
| Cullin-associated NEDD8-dissociated protein 1; | Q6ZQ38\|CAND1 | protein binding | 0 | 16 | 7 |
| Serine/threonine-protein phosphatase 2A 65 kDa | Q76MZ3\|2AAA | antigen binding | 2 | 9 | 5 |
