# Supplementary material for: Synthesis and Evaluation of a Novel Adenosine-Ribose Probe for Global-Scale Profiling of Nucleoside and Nucleotide-Binding Proteins
Source: PLoS One. 2015 Feb 11;10(2):e0115644. doi: 10.1371/journal.pone.0115644 (PMC4324776; doi:10.1371/journal.pone.0115644)
Supplement: S2 Table — (DOCX) [file pone.0115644.s004.docx]

**Table S2.** GO analysis (by Scaffold) of the complete list of proteins identified from PBS Wash, Regeneration Wash and total cell lysate (control) of N_18_TG_2_ cells.

| Identified Proteins (757) | binding | | molecular function | Average Spectral Count | | |
| --- | --- | --- | --- | --- | --- | --- |
|  |  |  |  | PBS | Regen | Control |
| 60 kDa heat shock protein, mitochondrial; AltName: | lipopolysaccharide binding | double-stranded RNA binding | | 6 | 66 | 35 |
| Elongation factor 1-alpha 1; EF-1-alpha-1; | GTP binding | translation elongation factor activity | | 11 | 68 | 80 |
| ATP synthase subunit beta, mitochondrial; Flags: | ATP binding | ATP binding | | 20 | 64 | 58 |
| Heat shock protein HSP 90-beta; Heat | protein binding | nitric-oxide synthase regulator activity | | 46 | 58 | 120 |
| Tubulin beta-5 chain. | GTP binding | structural constituent of cytoskeleton | | 9 | 59 | 78 |
| Tubulin alpha-1B chain; Alpha-tubulin | GTP binding | structural constituent of cytoskeleton | | 0 | 62 | 97 |
| Tubulin alpha-1C chain; Alpha-tubulin | GTP binding | structural constituent of cytoskeleton | | 0 | 60 | 70 |
| Tubulin alpha-1A chain; Alpha-tubulin | GTP binding | structural constituent of cytoskeleton | | 13 | 60 | 97 |
| Glyceraldehyde-3-phosphate dehydrogenase; | protein binding | glyceraldehyde-3-phosphate dehydrogenase (phosphorylating) activity | | 13 | 54 | 159 |
| Tubulin beta-4B chain; Tubulin beta-2C | GTP binding | structural constituent of cytoskeleton | | 6 | 51 | 67 |
| Actin, cytoplasmic 1; Beta-actin; | ATP binding | protein binding | | 31 | 46 | 197 |
| Tubulin beta-2A chain OS=Mus musculus GN=Tubb2a PE=1 SV=1 | GTP binding | structural constituent of cytoskeleton | | 0 | 16 | 61 |
| Heat shock cognate 71 kDa protein; Heat | ATP binding | ATPase activity, coupled | | 21 | 48 | 102 |
| Glyceraldehyde-3-phosphate dehydrogenase OS=Mus musculus GN=Gm10566 PE=3 SV=1 |  |  | | 0 | 15 | 22 |
| Elongation factor 1-alpha 2 OS=Mus musculus GN=Eef1a2 PE=1 SV=1 | GTP binding | translation elongation factor activity | | 0 | 33 | 21 |
| Tubulin beta-3 chain. | GTP binding | structural constituent of cytoskeleton | | 0 | 37 | 49 |
| ATP synthase subunit alpha, mitochondrial; Flags: | ATP binding | ATP binding | | 8 | 34 | 19 |
| Actin, alpha cardiac muscle 1; AltName: | ATP binding | ATP binding | | 10 | 27 | 41 |
| Pyruvate kinase PKM; Pyruvate kinase | potassium ion binding | pyruvate kinase activity | | 25 | 32 | 57 |
| Heat shock protein HSP 90-alpha; Heat | ATP binding | nitric-oxide synthase regulator activity | | 38 | 31 | 78 |
| Fatty acid synthase; Includes: RecName: | drug binding | [acyl-carrier-protein] S-malonyltransferase activity | | 6 | 28 | 16 |
| Elongation factor 2; EF-2. | GTP binding | translation elongation factor activity | | 15 | 28 | 80 |
| Peroxiredoxin-1; Macrophage 23 kDa | protein binding | thioredoxin peroxidase activity | | 2 | 29 | 9 |
| Dihydropyrimidinase-related protein 3; DRP-3; | SH3 domain binding | SH3 domain binding | | 3 | 24 | 10 |
| Endoplasmin; 94 kDa glucose-regulated | ATP binding | RNA binding | | 9 | 22 | 30 |
| T-complex protein 1 subunit beta; TCP-1-beta; | ATP binding | ATP binding | | 12 | 24 | 17 |
| Stress-70 protein, mitochondrial; 75 | ATP binding | protein binding | | 5 | 23 | 20 |
| D-3-phosphoglycerate dehydrogenase; 3-PGDH; | NAD binding | NAD binding | | 0 | 23 | 13 |
| T-complex protein 1 subunit delta; TCP-1-delta; | ATP binding | protein binding | | 4 | 25 | 10 |
| T-complex protein 1 subunit epsilon; | ATP binding | protein binding | | 3 | 21 | 13 |
| ATP synthase subunit O, mitochondrial; AltName: | steroid binding | steroid binding | | 0 | 21 | 2 |
| Peroxiredoxin-2; Thiol-specific | protein binding | peroxidase activity | | 1 | 21 | 9 |
| T-complex protein 1 subunit gamma; TCP-1-gamma; | ATP binding | protein binding | | 7 | 20 | 13 |
| 60S ribosomal protein L12. | RNA binding | RNA binding | | 3 | 19 | 10 |
| Poly(rC)-binding protein 1; Alpha-CP1; | protein binding | translation activator activity | | 1 | 16 | 9 |
| 40S ribosomal protein S3. | mRNA binding | mRNA binding | | 0 | 18 | 6 |
| Heterogeneous nuclear ribonucleoprotein K; | protein binding | RNA binding | | 1 | 16 | 11 |
| Cullin-associated NEDD8-dissociated protein 1; | protein binding | protein binding | | 0 | 16 | 7 |
| T-complex protein 1 subunit theta; TCP-1-theta; | ATP binding | protein binding | | 4 | 21 | 9 |
| Stress-induced-phosphoprotein 1; STI1; | protein binding | protein binding | | 0 | 18 | 5 |
| Polyadenylate-binding protein 1; PABP-1; | protein binding | nucleotide binding | | 0 | 13 | 3 |
| T-complex protein 1 subunit alpha; TCP-1-alpha; | ATP binding | protein binding | | 8 | 19 | 9 |
| 40S ribosomal protein S19. |  | structural constituent of ribosome | | 0 | 17 | 6 |
| 78 kDa glucose-regulated protein; GRP-78; | protein binding | misfolded protein binding | | 6 | 17 | 51 |
| Importin subunit beta-1; Karyopherin | protein binding | protein transporter activity | | 0 | 12 | 7 |
| Glycine--tRNA ligase; Diadenosine | ATP binding | glycine-tRNA ligase activity | | 0 | 11 | 8 |
| 14-3-3 protein zeta/delta; Protein | protein binding | protein domain specific binding | | 20 | 14 | 29 |
| Far upstream element-binding protein 2; | mRNA binding | mRNA binding | | 0 | 14 | 0 |
| Serine/threonine-protein phosphatase 2A 65 kDa | antigen binding | protein serine/threonine phosphatase activity | | 2 | 9 | 5 |
| Electron transfer flavoprotein subunit alpha, | FAD binding | oxidoreductase activity | | 0 | 11 | 5 |
| Transitional endoplasmic reticulum ATPase; TER | protein binding | polyubiquitin binding | | 3 | 12 | 20 |
| Eukaryotic translation initiation factor 3 subunit I; | translation initiation factor activity | translation initiation factor activity | | 0 | 9 | 1 |
| Nucleosome assembly protein 1-like 1; AltName: | protein binding | protein binding | | 2 | 9 | 7 |
| 40S ribosomal protein S17. |  | structural constituent of ribosome | | 0 | 9 | 4 |
| ATP synthase subunit d, mitochondrial; ATPase |  | hydrogen ion transmembrane transporter activity | | 0 | 9 | 3 |
| 40S ribosomal protein S18; Ke-3; | rRNA binding | protein binding | | 0 | 9 | 5 |
| T-complex protein 1 subunit eta; TCP-1-eta; | ATP binding | protein binding | | 3 | 13 | 21 |
| Alanine--tRNA ligase, cytoplasmic; AltName: | tRNA binding | alanine-tRNA ligase activity | | 3 | 12 | 11 |
| 14-3-3 protein theta; 14-3-3 protein | protein binding | protein domain specific binding | | 13 | 14 | 21 |
| L-lactate dehydrogenase A chain; LDH-A; | protein binding | L-lactate dehydrogenase activity | | 11 | 11 | 47 |
| Far upstream element-binding protein 1; FBP; | protein binding | RNA binding | | 0 | 10 | 0 |
| Cofilin-1; Cofilin, non-muscle isoform. |  |  | | 7 | 13 | 19 |
| 40S ribosomal protein S12 OS=Mus musculus GN=Rps12 PE=2 SV=1 |  |  | | 0 | 11 | 3 |
| Uncharacterized protein (Fragment) OS=Mus musculus GN=Trap1 PE=3 SV=1 |  |  | | 0 | 10 | 6 |
| Poly(rC)-binding protein 2; Alpha-CP2; | RNA binding | RNA binding | | 2 | 11 | 8 |
| Uncharacterized protein OS=Mus musculus GN=Pcbp3 PE=4 SV=1 |  |  | | 0 | 9 | 0 |
| AP-1 complex subunit beta-1; AltName: | protein binding | protein transporter activity | | 0 | 7 | 4 |
| T-complex protein 1 subunit zeta; TCP-1-zeta; | ATP binding | protein binding | | 9 | 10 | 11 |
| Eukaryotic translation initiation factor 3 subunit F; | translation initiation factor activity | ubiquitin-specific protease activity | | 0 | 7 | 3 |
| Microtubule-associated protein 1B; MAP-1B; | protein binding | cytoskeletal regulatory protein binding | | 6 | 9 | 10 |
| Prohibitin-2; B-cell | protein binding | protein binding | | 2 | 8 | 5 |
| 40S ribosomal protein S16. | RNA binding | RNA binding | | 0 | 8 | 2 |
| 60S ribosomal protein L13; A52. |  | structural constituent of ribosome | | 0 | 9 | 5 |
| Trifunctional purine biosynthetic protein | ATP binding | phosphoribosylformylglycinamidine cyclo-ligase activity | | 0 | 7 | 3 |
| 14-3-3 protein epsilon; 14-3-3E. | protein binding | potassium channel regulator activity | | 7 | 9 | 24 |
| 14-3-3 protein gamma; Contains: 14-3-3 | protein binding | protein domain specific binding | | 13 | 7 | 21 |
| Dihydropyrimidinase-related protein 2; DRP-2; | protein kinase binding | hydrolase activity, acting on carbon-nitrogen (but not peptide) bonds, in cyclic amides | | 0 | 10 | 0 |
| Crmp1 protein OS=Mus musculus GN=Crmp1 PE=2 SV=1 |  |  | | 0 | 9 | 3 |
| Heat shock protein 105 kDa; 42 degrees | ATP binding | protein binding | | 8 | 8 | 7 |
| Multifunctional protein ADE2; Includes: RecName: | ATP binding | phosphoribosylaminoimidazolesuccinocarboxamide synthase activity | | 0 | 8 | 2 |
| Uncharacterized protein OS=Mus musculus GN=Eif3b PE=3 SV=1 |  |  | | 0 | 7 | 2 |
| Arginine--tRNA ligase, cytoplasmic; AltName: | tRNA binding | arginine binding | | 2 | 7 | 5 |
| 60S ribosomal protein L23. | protein binding | protein binding | | 1 | 8 | 2 |
| 26S protease regulatory subunit 6A; 26S | ATP binding | nucleoside-triphosphatase activity | | 0 | 6 | 3 |
| Histone H2A type 1. | DNA binding | DNA binding | | 0 | 6 | 15 |
| 40S ribosomal protein S13. | mRNA binding | mRNA binding | | 0 | 7 | 0 |
| Dynactin subunit 2; 50 kDa | protein binding | motor activity | | 0 | 8 | 0 |
| 40S ribosomal protein S10. |  | structural constituent of ribosome | | 0 | 7 | 0 |
| Uncharacterized protein OS=Mus musculus GN=Rps5 PE=3 SV=1 |  |  | | 4 | 7 | 4 |
| 60S acidic ribosomal protein P2. |  | structural constituent of ribosome | | 0 | 7 | 1 |
| Dihydrolipoyllysine-residue acetyltransferase |  | dihydrolipoyllysine-residue acetyltransferase activity | | 0 | 6 | 3 |
| 26S protease regulatory subunit 6B; 26S | ATP binding | nucleoside-triphosphatase activity | | 0 | 5 | 2 |
| 60S ribosomal protein L23a. | rRNA binding | nucleotide binding | | 1 | 6 | 2 |
| Myosin light polypeptide 6; 17 kDa | calcium ion binding | actin-dependent ATPase activity | | 0 | 5 | 2 |
| 40S ribosomal protein S14. | mRNA 5'-UTR binding | translation regulator activity | | 0 | 5 | 7 |
| Lon protease homolog, mitochondrial; AltName: | ATP binding | ATPase activity | | 0 | 6 | 8 |
| Nucleophosmin; NPM; Nucleolar | protein binding | protein kinase inhibitor activity | | 2 | 6 | 15 |
| 26S proteasome non-ATPase regulatory subunit 14; | protein binding | metal ion binding | | 0 | 4 | 0 |
| 40S ribosomal protein S25. |  | structural constituent of ribosome | | 0 | 6 | 2 |
| 60S ribosomal protein L22; AltName: | RNA binding | heparin binding | | 0 | 6 | 2 |
| 60S ribosomal protein L31. |  | structural constituent of ribosome | | 0 | 5 | 3 |
| V-type proton ATPase subunit E 1; V-ATPase | protein binding | hydrogen ion transporting ATPase activity, rotational mechanism | | 0 | 5 | 2 |
| ADP-ribosylation factor 3. | GTP binding | GTP binding | | 5 | 5 | 1 |
| Heterogeneous nuclear ribonucleoprotein A/B; | protein binding | nucleotide binding | | 5 | 6 | 17 |
| ADP/ATP translocase 1; ADP,ATP carrier | protein binding | transporter activity | | 1 | 5 | 4 |
| 26S protease regulatory subunit 10B; AltName: | ATP binding | nucleoside-triphosphatase activity | | 0 | 5 | 1 |
| Monofunctional C1-tetrahydrofolate synthase, | ATP binding | ATP binding | | 0 | 5 | 0 |
| Glucosidase 2 subunit beta; 80K-H | calcium ion binding | RNA binding | | 0 | 5 | 0 |
| 40S ribosomal protein SA; 37 kDa | laminin binding | structural constituent of ribosome | | 5 | 5 | 7 |
| Myosin regulatory light chain 12B; AltName: | calcium ion binding | calcium ion binding | | 0 | 5 | 0 |
| 60S ribosomal protein L27. |  | structural constituent of ribosome | | 0 | 4 | 1 |
| Exportin-1; Exp1; Chromosome | protein binding | protein transporter activity | | 0 | 4 | 5 |
| Histone H1.4; H1 VAR.2; AltName: | DNA binding | protein binding | | 0 | 5 | 4 |
| 40S ribosomal protein S11. | rRNA binding | rRNA binding | | 0 | 4 | 2 |
| Eukaryotic translation initiation factor 3 subunit H; | translation initiation factor activity | translation initiation factor activity | | 0 | 3 | 1 |
| 60S ribosomal protein L6; AltName: |  | structural constituent of ribosome | | 1 | 4 | 6 |
| Elongation factor 1-gamma; EF-1-gamma; AltName: | translation elongation factor activity | translation elongation factor activity | | 0 | 8 | 6 |
| Calnexin; Flags: Precursor. | calcium ion binding | protein binding | | 1 | 6 | 7 |
| 40S ribosomal protein S20. | RNA binding | RNA binding | | 0 | 5 | 2 |
| 40S ribosomal protein S7. |  | structural constituent of ribosome | | 1 | 5 | 7 |
| MCG13402, isoform CRA_a OS=Mus musculus GN=Ptbp1 PE=2 SV=1 |  |  | | 0 | 4 | 10 |
| Dolichyl-diphosphooligosaccharide--protein |  | dolichyl-diphosphooligosaccharide-protein glycotransferase activity | | 0 | 5 | 0 |
| Asparagine synthetase [glutamine-hydrolyzing]; | cofactor binding | asparagine synthase (glutamine-hydrolyzing) activity | | 3 | 5 | 8 |
| Protein dpy-30 homolog; Dpy-30-like |  |  | | 0 | 4 | 0 |
| Caprin-1; Cytoplasmic activation- and | RNA binding | RNA binding | | 0 | 4 | 2 |
| Ras-related protein Rab-1A; AltName: | GTP binding | protein binding | | 1 | 6 | 5 |
| 60S ribosomal protein L35. |  | structural constituent of ribosome | | 0 | 5 | 0 |
| Prohibitin; B-cell receptor-associated |  |  | | 1 | 4 | 1 |
| Importin subunit alpha-1; Importin | protein binding | protein transporter activity | | 0 | 4 | 1 |
| Transcription intermediary factor 1-beta; | protein binding | transcription coactivator activity | | 0 | 3 | 0 |
| Methionine--tRNA ligase, cytoplasmic; AltName: | tRNA binding | ATP binding | | 0 | 3 | 4 |
| Elongation factor 1-delta; EF-1-delta. | translation elongation factor activity | translation elongation factor activity | | 2 | 3 | 0 |
| 60S acidic ribosomal protein P0; 60S |  | structural constituent of ribosome | | 2 | 3 | 12 |
| 60S ribosomal protein L21. |  | structural constituent of ribosome | | 1 | 2 | 1 |
| V-type proton ATPase subunit B, brain isoform; | ATP binding | hydrolase activity, acting on acid anhydrides, catalyzing transmembrane movement of substances | | 0 | 4 | 0 |
| Ras-related protein Rab-1B. | GTP binding | GTP binding | | 0 | 5 | 1 |
| 26S proteasome non-ATPase regulatory subunit 11; | protein binding | protein binding | | 0 | 5 | 0 |
| Cytochrome c oxidase subunit 5A, mitochondrial; | metal ion binding | metal ion binding | | 1 | 5 | 0 |
| Ras-related protein Rab-11B; Flags: Precursor. | GTP binding | protein binding | | 0 | 4 | 0 |
| Synaptic vesicle membrane protein VAT-1 homolog. | zinc ion binding | oxidoreductase activity | | 0 | 4 | 0 |
| Exportin-2; Exp2; Chromosome | protein binding | protein binding | | 0 | 4 | 3 |
| Small glutamine-rich tetratricopeptide | protein heterodimerization activity | protein heterodimerization activity | | 0 | 3 | 0 |
| 40S ribosomal protein S2; 40S ribosomal | RNA binding | RNA binding | | 0 | 5 | 8 |
| 39S ribosomal protein L12, mitochondrial; |  | structural constituent of ribosome | | 0 | 5 | 3 |
| 40S ribosomal protein S3a; Protein | protein binding | protein binding | | 0 | 4 | 6 |
| Importin-7; Imp7; Ran-binding | protein binding | protein binding | | 0 | 4 | 0 |
| Aspartate--tRNA ligase, cytoplasmic; AltName: | ATP binding | nucleic acid binding | | 0 | 3 | 0 |
| Splicing factor 3B subunit 3; AltName: | nucleic acid binding | nucleic acid binding | | 0 | 3 | 0 |
| Dolichyl-diphosphooligosaccharide--protein |  | dolichyl-diphosphooligosaccharide-protein glycotransferase activity | | 0 | 4 | 1 |
| Ras-related protein Rab-5C. | GTP binding | protein binding | | 0 | 4 | 0 |
| Eukaryotic translation initiation factor 4 gamma 1; | translation initiation factor activity | translation initiation factor activity | | 0 | 4 | 0 |
| Spermidine synthase; SPDSY; AltName: |  | spermidine synthase activity | | 0 | 3 | 1 |
| 39S ribosomal protein L10, mitochondrial; |  | structural constituent of ribosome | | 0 | 3 | 0 |
| Serine/threonine-protein phosphatase PP1-alpha | ribonucleoprotein binding | metal ion binding | | 1 | 3 | 2 |
| C-1-tetrahydrofolate synthase, cytoplasmic; | ATP binding | ATP binding | | 0 | 3 | 0 |
| UDP-glucose:glycoprotein glucosyltransferase 1; | protein binding | UDP-glucose:glycoprotein glucosyltransferase activity | | 0 | 3 | 2 |
| Asparagine--tRNA ligase, cytoplasmic; AltName: | ATP binding | asparagine-tRNA ligase activity | | 0 | 3 | 1 |
| SUMO-activating enzyme subunit 1; AltName: | protein C-terminus binding | protein C-terminus binding | | 0 | 3 | 0 |
| Importin subunit alpha-7; Importin | protein binding | protein transporter activity | | 0 | 3 | 1 |
| Ras-related protein Rab-5B. | GTP binding | protein binding | | 0 | 3 | 0 |
| Cell division control protein 42 homolog; AltName: | protein binding | GTPase activity | | 4 | 3 | 2 |
| UPF0568 protein C14orf166 homolog. |  |  | | 0 | 4 | 0 |
| Histone H2B type 1-F/J/L; H2B 291A. | DNA binding | DNA binding | | 0 | 3 | 16 |
| Mitochondrial import receptor subunit TOM34; AltName: | protein binding | protein binding | | 0 | 2 | 0 |
| Ubiquilin-2; Chap1; DSK2 | protein binding | protein binding | | 0 | 2 | 0 |
| Eukaryotic translation initiation factor 5A-1; | ribosome binding | translation elongation factor activity | | 7 | 4 | 23 |
| Uncharacterized protein OS=Mus musculus GN=Atp2a2 PE=3 SV=1 |  |  | | 0 | 3 | 0 |
| Isoform 2 of Transportin-1 OS=Mus musculus GN=Tnpo1 |  |  | | 0 | 3 | 0 |
| 40S ribosomal protein S26. |  | structural constituent of ribosome | | 1 | 3 | 1 |
| E3 ubiquitin-protein ligase HUWE1; AltName: | DNA binding | DNA binding | | 0 | 2 | 0 |
| Ras-related protein Rab-2A. | GTP binding | GTP binding | | 0 | 2 | 0 |
| NADH-cytochrome b5 reductase 3; B5R; | ADP binding | cytochrome-b5 reductase activity | | 0 | 2 | 0 |
| Ribose-phosphate pyrophosphokinase 1; AltName: | protein homodimerization activity | ribose phosphate diphosphokinase activity | | 0 | 2 | 0 |
| Alpha-enolase; 2-phospho-D-glycerate | magnesium ion binding | magnesium ion binding | | 45 | 2 | 154 |
| MCG49690 OS=Mus musculus GN=Gm4987 PE=4 SV=1 |  |  | | 0 | 2 | 1 |
| Isoform 2 of Plasminogen activator inhibitor 1 RNA-binding protein OS=Mus musculus GN=Serbp1 |  |  | | 0 | 1 | 0 |
| 60S ribosomal protein L5. | 5S rRNA binding | protein binding | | 0 | 1 | 3 |
| ATP-citrate synthase; ATP-citrate | cofactor binding | succinate-CoA ligase (ADP-forming) activity | | 15 | 5 | 18 |
| Electron transfer flavoprotein subunit beta; |  | electron carrier activity | | 0 | 5 | 0 |
| 40S ribosomal protein S4, X isoform. | rRNA binding | rRNA binding | | 0 | 4 | 3 |
| Ubiquitin carboxyl-terminal hydrolase 5; AltName: | protein binding | omega peptidase activity | | 0 | 4 | 3 |
| 26S protease regulatory subunit 7; 26S | ATP binding | ATPase activity | | 0 | 4 | 2 |
| Proteasome activator complex subunit 3; AltName: | p53 binding | p53 binding | | 1 | 3 | 2 |
| Small nuclear ribonucleoprotein Sm D1; Sm-D1; |  |  | | 0 | 3 | 3 |
| Succinate dehydrogenase [ubiquinone] iron-sulfur | ubiquinone binding | succinate dehydrogenase (ubiquinone) activity | | 0 | 3 | 0 |
| Deoxyhypusine hydroxylase; DOHH; AltName: | metal ion binding | metal ion binding | | 0 | 3 | 0 |
| Exportin-T; Exportin(tRNA); AltName: | tRNA binding | tRNA binding | | 0 | 2 | 0 |
| 40S ribosomal protein S15a. |  | structural constituent of ribosome | | 0 | 3 | 1 |
| 26S protease regulatory subunit 8; 26S | ATP binding | ATPase activity | | 0 | 2 | 2 |
| 60S ribosomal protein L10a; CSA-19; | RNA binding | RNA binding | | 0 | 3 | 2 |
| Small nuclear ribonucleoprotein Sm D3; Sm-D3; |  |  | | 0 | 2 | 1 |
| Eukaryotic translation initiation factor 3 subunit E; | translation initiation factor activity | translation initiation factor activity | | 0 | 2 | 2 |
| Nap1l4 protein OS=Mus musculus GN=Nap1l4 PE=2 SV=1 |  |  | | 1 | 2 | 3 |
| Transforming protein RhoA; Flags: Precursor. | protein binding | GTPase activity | | 2 | 2 | 0 |
| Dolichyl-diphosphooligosaccharide--protein | ribosome binding | transferase activity, transferring glycosyl groups | | 0 | 2 | 0 |
| Glutaminase kidney isoform, mitochondrial; GLS; | protein binding | glutaminase activity | | 0 | 1 | 0 |
| RuvB-like 1; 49 kDa TATA box-binding | ATP binding | ATP binding | | 0 | 1 | 0 |
| Uncharacterized protein OS=Mus musculus GN=Smarcc2 PE=2 SV=1 |  |  | | 0 | 1 | 0 |
| 26S proteasome non-ATPase regulatory subunit 2; | protein binding | enzyme regulator activity | | 0 | 3 | 1 |
| Acetyl-CoA acetyltransferase, mitochondrial; AltName: | coenzyme binding | acetyl-CoA C-acetyltransferase activity | | 0 | 3 | 3 |
| Ran-specific GTPase-activating protein; AltName: |  | GTPase activator activity | | 4 | 5 | 2 |
| Protein disulfide-isomerase A6; AltName: |  | protein disulfide oxidoreductase activity | | 6 | 3 | 15 |
| Importin-5; Imp5; Importin | protein binding | protein transporter activity | | 0 | 2 | 1 |
| Myosin-9; Cellular myosin heavy chain, | protein binding | microfilament motor activity | | 0 | 3 | 2 |
| Dynein light chain Tctex-type 1; AltName: | protein binding | motor activity | | 0 | 3 | 1 |
| Eukaryotic translation initiation factor 3 subunit K; | ribosome binding | translation initiation factor activity | | 0 | 3 | 0 |
| 40S ribosomal protein S21. | ribosome binding | ribosome binding | | 0 | 3 | 0 |
| Basic leucine zipper and W2 domain-containing protein |  |  | | 0 | 3 | 0 |
| Dynein light chain roadblock-type 1; AltName: | protein binding | motor activity | | 0 | 3 | 0 |
| Leucine--tRNA ligase, cytoplasmic; AltName: | ATP binding | ATP binding | | 1 | 3 | 2 |
| Peptidyl-prolyl cis-trans isomerase A; PPIase | peptide binding | peptide binding | | 15 | 3 | 96 |
| Uridine 5'-monophosphate synthase; UMP |  | orotidine-5'-phosphate decarboxylase activity | | 0 | 2 | 0 |
| Delta-1-pyrroline-5-carboxylate synthase; P5CS; | ATP binding | ATP binding | | 0 | 2 | 2 |
| Ribonuclease inhibitor; AltName: |  | ribonuclease inhibitor activity | | 0 | 2 | 0 |
| ATPase Asna1; Arsenical pump-driving | metal ion binding | ATPase activity | | 0 | 2 | 0 |
| Eukaryotic peptide chain release factor GTP-binding | GTP binding | translation release factor activity | | 0 | 2 | 0 |
| Cytochrome b-c1 complex subunit 2, mitochondrial; | metal ion binding | metal ion binding | | 1 | 2 | 2 |
| ATP synthase subunit e, mitochondrial; ATPase |  | ATPase activity | | 0 | 3 | 0 |
| 26S protease regulatory subunit 4; P26s4; | ATP binding | nucleoside-triphosphatase activity | | 0 | 2 | 1 |
| Importin subunit alpha-5; Importin | protein binding | protein transporter activity | | 0 | 2 | 0 |
| 26S proteasome non-ATPase regulatory subunit 12; |  |  | | 0 | 2 | 0 |
| ATP synthase F(0) complex subunit B1, mitochondrial; |  | hydrogen ion transporting ATP synthase activity, rotational mechanism | | 0 | 2 | 2 |
| Nuclear autoantigenic sperm protein; NASP. | protein binding | protein binding | | 1 | 2 | 0 |
| Sulfated glycoprotein 1; SGP-1; AltName: |  |  | | 0 | 2 | 0 |
| ATP synthase gamma chain OS=Mus musculus GN=Atp5c1 PE=3 SV=1 |  |  | | 0 | 3 | 1 |
| Phosphate carrier protein, mitochondrial; AltName: | protein complex binding | protein complex binding | | 0 | 2 | 3 |
| Elongation factor Tu, mitochondrial; Flags: | GTP binding | translation elongation factor activity | | 0 | 2 | 5 |
| Aminoacyl tRNA synthase complex-interacting | protein binding | protein binding | | 0 | 2 | 2 |
| Ribosomal protein S27 OS=Mus musculus GN=Gm17241 PE=3 SV=1 |  |  | | 0 | 2 | 2 |
| Retrovirus-related Env polyprotein from Fv-4 locus. |  | structural molecule activity | | 0 | 2 | 2 |
| Uncharacterized protein OS=Mus musculus GN=Atp5j2 PE=4 SV=1 |  |  | | 0 | 2 | 0 |
| Inosine-5'-monophosphate dehydrogenase 2; IMP | metal ion binding | IMP dehydrogenase activity | | 0 | 2 | 0 |
| Replication protein A 14 kDa subunit; RP-A p14; |  |  | | 0 | 2 | 0 |
| AH receptor-interacting protein; AIP; AltName: | protein binding | transcription cofactor activity | | 0 | 2 | 0 |
| 40S ribosomal protein S30. |  | structural constituent of ribosome | | 0 | 2 | 0 |
| 60S ribosomal protein L36a; 60S |  | structural constituent of ribosome | | 0 | 2 | 0 |
| Treacle protein; Treacher Collins |  |  | | 0 | 2 | 0 |
| Mitochondrial import inner membrane translocase | metal ion binding | metal ion binding | | 0 | 2 | 0 |
| 60S acidic ribosomal protein P1. |  | structural constituent of ribosome | | 1 | 2 | 8 |
| 60S ribosomal protein L24. |  | structural constituent of ribosome | | 1 | 2 | 1 |
| 60S ribosomal protein L30. |  | structural constituent of ribosome | | 2 | 2 | 2 |
| Farnesyl pyrophosphate synthase; FPP synthase; | metal ion binding | geranyltranstransferase activity | | 3 | 2 | 4 |
| Voltage-dependent anion-selective channel protein 1; | nucleotide binding | porin activity | | 0 | 2 | 7 |
| ATP synthase subunit delta, mitochondrial; AltName: |  | hydrogen ion transporting ATPase activity, rotational mechanism | | 0 | 2 | 2 |
| TBC1 domain family member 15; AltName: |  | Rab GTPase activator activity | | 0 | 2 | 0 |
| Succinate dehydrogenase [ubiquinone] flavoprotein | FAD binding | FAD binding | | 0 | 2 | 2 |
| Ragulator complex protein LAMTOR2; AltName: | protein binding | protein binding | | 0 | 2 | 0 |
| Tetratricopeptide repeat protein 1; TPR repeat |  |  | | 0 | 2 | 0 |
| Bifunctional glutamate/proline--tRNA ligase; AltName: | ATP binding | ATP binding | | 0 | 1 | 3 |
| Ran GTPase-activating protein 1; RanGAP1. |  | Ran GTPase activator activity | | 0 | 1 | 3 |
| Ubiquitin carboxyl-terminal hydrolase 15; AltName: |  | cysteine-type endopeptidase activity | | 0 | 1 | 1 |
| Isoform 2 of 2-oxoglutarate dehydrogenase, mitochondrial OS=Mus musculus GN=Ogdh |  |  | | 0 | 1 | 0 |
| Poly (ADP-ribose) polymerase family, member 1 OS=Mus musculus GN=Parp1 PE=2 SV=1 |  |  | | 0 | 1 | 0 |
| Hsc70-interacting protein; Hip; AltName: | dATP binding | dATP binding | | 0 | 1 | 1 |
| Septin-9; SL3-3 integration site 1 | GTP binding | GTP binding | | 0 | 1 | 0 |
| S-phase kinase-associated protein 1; AltName: | protein binding | ubiquitin-protein ligase activity | | 0 | 1 | 0 |
| Coatomer subunit beta'; Beta'-coat |  | structural molecule activity | | 0 | 1 | 0 |
| SUMO-activating enzyme subunit 2; AltName: | metal ion binding | ligase activity | | 0 | 1 | 0 |
| Protein arginine N-methyltransferase 5; AltName: | ribonucleoprotein binding | protein binding | | 0 | 1 | 0 |
| Trifunctional enzyme subunit alpha, mitochondrial; | NAD binding | acyl-CoA binding | | 0 | 1 | 0 |
| Phospholipase A-2-activating protein; PLA2P; | protein binding | phospholipase A2 activator activity | | 0 | 1 | 0 |
| Apoptosis regulator BAX. | protein binding | channel activity | | 0 | 1 | 0 |
| Sodium/potassium-transporting ATPase subunit alpha-1; | protein binding | sodium:potassium-exchanging ATPase activity | | 0 | 1 | 0 |
| Retinoid-inducible serine carboxypeptidase; AltName: |  | serine-type carboxypeptidase activity | | 0 | 1 | 0 |
| Stromal cell-derived factor 2-like protein 1; | ATPase binding | ATPase binding | | 0 | 1 | 0 |
| Acid ceramidase; AC; ACDase; Acid |  | ceramidase activity | | 0 | 1 | 0 |
| LETM1 and EF-hand domain-containing protein 1, | metal ion binding | metal ion binding | | 0 | 1 | 0 |
| 60S ribosomal protein L28. |  | structural constituent of ribosome | | 0 | 1 | 0 |
| 40S ribosomal protein S15; RIG protein. | protein binding | RNA binding | | 1 | 1 | 4 |
| ADP-ribosylation factor-like protein 1. | GTP binding | metal ion binding | | 1 | 1 | 2 |
| NADH dehydrogenase [ubiquinone] flavoprotein 2, | metal ion binding | electron carrier activity | | 1 | 1 | 0 |
| Hypoxia up-regulated protein 1; GRP-170; | ATP binding | ATP binding | | 4 | 1 | 2 |
| Clathrin heavy chain 1. | double-stranded RNA binding | structural molecule activity | | 3 | 1 | 7 |
| Uncharacterized protein OS=Mus musculus GN=Vars PE=3 SV=1 |  |  | | 1 | 1 | 4 |
| Apoptosis inhibitor 5; API-5; AltName: | fibroblast growth factor binding | fibroblast growth factor binding | | 2 | 1 | 1 |
| Nuclease-sensitive element-binding protein 1; | protein binding | RNA binding | | 1 | 2 | 0 |
| Coatomer subunit beta OS=Mus musculus GN=Copb1 PE=3 SV=1 |  |  | | 0 | 1 | 1 |
| Importin subunit alpha-3; Importin | protein binding | protein transporter activity | | 0 | 1 | 1 |
| Surfeit locus protein 4. |  |  | | 0 | 1 | 0 |
| mRNA turnover protein 4 homolog. |  |  | | 0 | 1 | 0 |
| Ras-related protein Rab-7a. | GTP binding | protein binding | | 1 | 1 | 0 |
| Cytochrome b-c1 complex subunit 1, mitochondrial; | metal ion binding | catalytic activity | | 1 | 1 | 0 |
| 40S ribosomal protein S24 OS=Mus musculus GN=Rps24 PE=3 SV=1 |  |  | | 0 | 2 | 2 |
| Ras-related protein Rab-21; Rab-12; | GTP binding | protein binding | | 0 | 2 | 0 |
| Dynein heavy chain 2, axonemal OS=Mus musculus GN=Dnah2 PE=2 SV=1 |  |  | | 0 | 1 | 0 |
| Small nuclear ribonucleoprotein-associated protein B; |  |  | | 0 | 1 | 0 |
| Protein transport protein Sec31A; AltName: |  |  | | 0 | 1 | 1 |
| Eukaryotic translation initiation factor 3 subunit C OS=Mus musculus GN=Eif3c PE=1 SV=1 | translation initiation factor activity | translation initiation factor activity | | 0 | 1 | 0 |
| Uncharacterized protein OS=Mus musculus GN=Asap2 PE=4 SV=1 |  |  | | 0 | 1 | 0 |
| Histone deacetylase 1; HD1. | protein binding | transcription factor activity | | 0 | 1 | 0 |
| Protein transport protein Sec61 subunit alpha isoform | ribosome binding | ribosome binding | | 0 | 1 | 0 |
| Nodal modulator 1; Flags: Precursor. | carbohydrate binding | carbohydrate binding | | 0 | 1 | 0 |
| Protein TBRG4; Transforming growth |  | protein kinase activity | | 0 | 1 | 0 |
| Transmembrane emp24 domain-containing protein 10; |  |  | | 0 | 1 | 0 |
| 28S ribosomal protein S29, mitochondrial; |  |  | | 0 | 1 | 0 |
| Large proline-rich protein BAG6; BAG | protein binding | polyubiquitin binding | | 0 | 1 | 0 |
| E3 ubiquitin-protein ligase NEDD4; AltName: | protein binding | sodium channel inhibitor activity | | 0 | 1 | 0 |
| 60S ribosomal protein L7a; Surfeit | protein binding | protein binding | | 1 | 1 | 6 |
| Ubiquitin-like modifier-activating enzyme 1; AltName: | ATP binding | small protein activating enzyme activity | | 19 | 6 | 21 |
| Leucine-rich PPR motif-containing protein, | protein binding | RNA binding | | 0 | 2 | 0 |
| Pyruvate dehydrogenase E1 component subunit beta, |  | pyruvate dehydrogenase (acetyl-transferring) activity | | 0 | 2 | 2 |
| 26S proteasome non-ATPase regulatory subunit 1; | protein binding | enzyme regulator activity | | 0 | 2 | 0 |
| GTP-binding nuclear protein Ran; GTPase | GTP binding | protein binding | | 8 | 2 | 16 |
| Eukaryotic translation elongation factor 1 epsilon-1; | protein binding | protein binding | | 0 | 1 | 0 |
| Peroxiredoxin-6; 1-Cys peroxiredoxin; |  | hydrolase activity | | 3 | 1 | 5 |
| Myristoylated alanine-rich C-kinase substrate; | protein binding | protein binding | | 1 | 2 | 0 |
| Aminoacyl tRNA synthase complex-interacting | tRNA binding | cytokine activity | | 0 | 2 | 3 |
| Ataxin-2-like protein. |  |  | | 0 | 2 | 0 |
| Lysine--tRNA ligase; Lysyl-tRNA | metal ion binding | nucleic acid binding | | 1 | 2 | 1 |
| Probable ATP-dependent RNA helicase DDX5; AltName: | protein binding | RNA helicase activity | | 0 | 1 | 4 |
| Uncharacterized protein OS=Mus musculus GN=Hk2 PE=3 SV=1 |  |  | | 0 | 1 | 0 |
| NADH-ubiquinone oxidoreductase 75 kDa subunit, | 4 iron, 4 sulfur cluster binding | electron carrier activity | | 0 | 1 | 0 |
| ATP-dependent 6-phosphofructokinase, liver type; | ATP binding | 6-phosphofructokinase activity | | 0 | 1 | 0 |
| Filamin, alpha OS=Mus musculus GN=Flna PE=4 SV=1 |  |  | | 0 | 1 | 1 |
| V-type proton ATPase catalytic subunit A; | ATP binding | hydrogen ion transporting ATPase activity, rotational mechanism | | 0 | 1 | 2 |
| Beta-centractin; Actin-related protein | ATP binding | ATP binding | | 0 | 1 | 1 |
| Dynamin-1-like protein; Dynamin family | GTP binding | lipid binding | | 0 | 1 | 1 |
| Uncharacterized protein OS=Mus musculus GN=Cstf2 PE=4 SV=1 |  |  | | 0 | 1 | 0 |
| Son of sevenless homolog 2 OS=Mus musculus GN=Sos2 PE=1 SV=2 |  |  | | 0 | 1 | 0 |
| Putative pre-mRNA-splicing factor ATP-dependent RNA | ATP binding | ATP-dependent helicase activity | | 0 | 1 | 0 |
| Estradiol 17-beta-dehydrogenase 12; AltName: | collagen binding | estradiol 17-beta-dehydrogenase activity | | 0 | 1 | 0 |
| Pyrroline-5-carboxylate reductase 3; P5C | protein binding | pyrroline-5-carboxylate reductase activity | | 0 | 1 | 0 |
| Triosephosphate isomerase; TIM; AltName: |  | triose-phosphate isomerase activity | | 10 | 1 | 7 |
| Heat shock protein 4 OS=Mus musculus GN=Hspa4 PE=2 SV=1 |  |  | | 11 | 1 | 10 |
| Ras-related protein Rap-1b; GTP-binding | GTP binding | protein binding | | 1 | 1 | 2 |
| 60S ribosomal protein L38. |  | structural constituent of ribosome | | 0 | 1 | 0 |
| Small nuclear ribonucleoprotein Sm D2; Sm-D2; |  |  | | 0 | 1 | 1 |
| Heterogeneous nuclear ribonucleoprotein H; | RNA binding | nucleotide binding | | 0 | 1 | 9 |
| Ubiquitin carboxyl-terminal hydrolase 14; AltName: |  | cysteine-type peptidase activity | | 0 | 1 | 2 |
| Isocitrate dehydrogenase 3 (NAD+) beta OS=Mus musculus GN=Idh3b PE=2 SV=1 |  |  | | 0 | 1 | 1 |
| Uncharacterized protein OS=Mus musculus GN=Klc1 PE=4 SV=2 |  |  | | 0 | 1 | 0 |
| Ribosomal RNA processing protein 1 homolog A; |  |  | | 0 | 1 | 1 |
| Ataxin-10; Brain protein E46; AltName: | protein binding | protein binding | | 0 | 1 | 0 |
| Uncharacterized protein OS=Mus musculus GN=Zfp644 PE=4 SV=1 |  |  | | 0 | 1 | 0 |
| Ribonucleoprotein PTB-binding 1; AltName: | RNA binding | nucleotide binding | | 0 | 1 | 0 |
| GTP-binding protein SAR1a. | GTP binding | GTP binding | | 0 | 1 | 0 |
| MCG67985 OS=Mus musculus GN=Uqcrb PE=4 SV=1 |  |  | | 0 | 1 | 0 |
| Tryptophan--tRNA ligase, cytoplasmic; AltName: | ATP binding | ATP binding | | 5 | 1 | 1 |
| BAG family molecular chaperone regulator 3; | protein binding | protein binding | | 0 | 1 | 0 |
| Isoform 2 of Neutral alpha-glucosidase AB OS=Mus musculus GN=Ganab |  |  | | 0 | 0 | 0 |
| Ribosomal protein L26 (Fragment) OS=Mus musculus GN=Rpl26 PE=4 SV=1 |  |  | | 0 | 0 | 1 |
| Splicing factor 3B subunit 4. | RNA binding | nucleotide binding | | 0 | 0 | 1 |
| ATP synthase protein 8; A6L; AltName: |  | hydrogen ion transmembrane transporter activity | | 0 | 0 | 0 |
| Uncharacterized protein OS=Mus musculus GN=Ipo9 PE=4 SV=1 |  |  | | 0 | 0 | 1 |
| DnaJ homolog subfamily A member 3, mitochondrial; | protein binding | small GTPase regulator activity | | 0 | 0 | 0 |
| Cytochrome b-c1 complex subunit 6, mitochondrial OS=Mus musculus GN=Uqcrh PE=1 SV=2 |  | ubiquinol-cytochrome-c reductase activity | | 0 | 0 | 0 |
| Dephospho-CoA kinase domain-containing protein. | ATP binding | ATP binding | | 0 | 0 | 0 |
| Uncharacterized protein OS=Mus musculus GN=Ubap2l PE=4 SV=1 |  |  | | 0 | 0 | 0 |
| Solute carrier family 1 (Glutamate/neutral amino acid transporter), member 4 OS=Mus musculus GN=Slc1a4 PE=2 SV=1 |  |  | | 0 | 0 | 0 |
| Q8C872\|Q8C872 |  |  | | 0 | 0 | 0 |
| UBX domain-containing protein 1; AltName: |  |  | | 0 | 0 | 0 |
| Eukaryotic translation initiation factor 3 subunit G; | translation initiation factor activity | translation initiation factor activity | | 0 | 0 | 0 |
| Uncharacterized protein OS=Mus musculus GN=Try5 PE=3 SV=1 |  |  | | 4 | 0 | 1 |
| Proliferating cell nuclear antigen; PCNA; | protein binding | DNA polymerase processivity factor activity | | 5 | 0 | 3 |
| Isoform M1 of Pyruvate kinase isozymes M1/M2 OS=Mus musculus GN=Pkm2 |  |  | | 14 | 11 | 0 |
| Eukaryotic initiation factor 4A-I; eIF-4A-I; | ATP binding | ATP-dependent helicase activity | | 15 | 2 | 25 |
| Transgelin-2; SM22-beta. |  |  | | 3 | 2 | 15 |
| F-actin-capping protein subunit beta; AltName: | protein binding | beta-tubulin binding | | 0 | 1 | 4 |
| Copine-3; Copine III. |  | protein serine/threonine kinase activity | | 0 | 1 | 0 |
| Prefoldin subunit 2. |  |  | | 0 | 2 | 0 |
| Reticulon-4; Neurite outgrowth | protein binding | protein binding | | 0 | 1 | 0 |
| PR domain zinc finger protein 16; PR | protein binding | transcription coactivator activity | | 0 | 1 | 0 |
| Isoform 2 of Proteasome-associated protein ECM29 homolog OS=Mus musculus GN=Ecm29 |  |  | | 0 | 1 | 0 |
| C-terminal-binding protein 1; CtBP1. | NAD binding | oxidoreductase activity, acting on the CH-OH group of donors, NAD or NADP as acceptor | | 0 | 1 | 0 |
| Guanine nucleotide-binding protein G(I)/G(S)/G(T) | spectrin binding | signal transducer activity | | 0 | 1 | 2 |
| Uncharacterized protein OS=Mus musculus GN=Lrrc59 PE=2 SV=1 |  |  | | 0 | 1 | 0 |
| Cleavage and polyadenylation specificity factor | mRNA binding | mRNA binding | | 0 | 1 | 1 |
| COP9 signalosome complex subunit 3; SGN3; | protein binding | protein binding | | 0 | 1 | 1 |
| Ragulator complex protein LAMTOR3; Late | protein binding | protein binding | | 0 | 1 | 0 |
| E9Q0U1\|E9Q0U1 |  |  | | 0 | 1 | 0 |
| Ubiquitin fusion degradation protein 1 homolog; |  |  | | 0 | 1 | 0 |
| Inorganic pyrophosphatase; AltName: | magnesium ion binding | magnesium ion binding | | 0 | 1 | 0 |
| Vesicle-fusing ATPase; AltName: | metal ion binding | ATPase activity, coupled | | 0 | 1 | 0 |
| Protein phosphatase 1G; Fibroblast | metal ion binding | phosphoprotein phosphatase activity | | 0 | 1 | 0 |
| HEAT repeat-containing protein 3. |  |  | | 0 | 1 | 0 |
| Coiled-coil domain-containing protein 47; AltName: | calcium ion binding | calcium ion binding | | 0 | 1 | 0 |
| Phosphatidylinositide phosphatase SAC1; AltName: |  | phosphatidylinositol-4-phosphate phosphatase activity | | 0 | 1 | 0 |
| Centrin-2; Caltractin isoform 1. | ATP binding | ATP-dependent helicase activity | | 0 | 1 | 0 |
| Lipoamide acyltransferase component of branched-chain |  | dihydrolipoyllysine-residue (2-methylpropanoyl)transferase activity | | 0 | 1 | 0 |
| B2RPU8\|B2RPU8 |  |  | | 0 | 1 | 0 |
| Developmentally-regulated GTP-binding protein 2; | GTP binding | GTP binding | | 0 | 1 | 0 |
| Actin-like protein 6A; 53 kDa | chromatin binding | protein binding | | 1 | 1 | 0 |
| Cyclin-dependent kinase 1; CDK1; AltName: | protein binding | kinase activity | | 1 | 1 | 0 |
| Basic leucine zipper and W2 domain-containing protein |  |  | | 1 | 1 | 0 |
| Thioredoxin-dependent peroxide reductase, | identical protein binding | identical protein binding | | 2 | 1 | 0 |
| Hsp90 co-chaperone Cdc37; Hsp90 | Hsp90 protein binding | Hsp90 protein binding | | 2 | 1 | 2 |
| Protein disulfide-isomerase; PDI; AltName: |  | protein disulfide oxidoreductase activity | | 2 | 1 | 6 |
| Nucleolin; Protein C23. | protein binding | nucleotide binding | | 5 | 1 | 17 |
| Prostaglandin E synthase 3; Cytosolic | unfolded protein binding | unfolded protein binding | | 3 | 1 | 11 |
| Calmodulin; CaM. | calcium ion binding | protein binding | | 6 | 1 | 0 |
| Acidic leucine-rich nuclear phosphoprotein 32 family | histone binding | phosphatase inhibitor activity | | 7 | 1 | 4 |
| Fructose-bisphosphate aldolase A; AltName: | protease binding | fructose-bisphosphate aldolase activity | | 12 | 1 | 22 |
| Transketolase; TK; P68. | cofactor binding | transketolase activity | | 15 | 1 | 7 |
| Uncharacterized protein OS=Mus musculus GN=Gm10126 PE=4 SV=1 |  |  | | 0 | 1 | 2 |
| Elongation factor 1-beta; EF-1-beta. | translation elongation factor activity | translation elongation factor activity | | 0 | 1 | 2 |
| Spliceosome RNA helicase Ddx39b; 56 kDa | ATP binding | ATP-dependent RNA helicase activity | | 1 | 1 | 3 |
| Uncharacterized protein OS=Mus musculus GN=Rbbp4 PE=4 SV=1 |  |  | | 0 | 1 | 0 |
| ADP/ATP translocase 2 OS=Mus musculus GN=Slc25a5 PE=1 SV=3 |  | transporter activity | | 0 | 1 | 0 |
| Coatomer subunit gamma-1; Gamma-1-coat |  | structural molecule activity | | 0 | 1 | 0 |
| Cysteine-rich protein 2; CRP-2; AltName: | protein binding | zinc ion binding | | 0 | 1 | 0 |
| Histone H4. | DNA binding | protein binding | | 0 | 0 | 13 |
| Uncharacterized protein OS=Mus musculus GN=Sept2 PE=4 SV=1 |  |  | | 0 | 0 | 2 |
| Splicing factor 1; CW17; AltName: | RNA binding | zinc ion binding | | 0 | 0 | 0 |
| DnaJ homolog subfamily B member 11; AltName: | protein binding | protein binding | | 0 | 0 | 0 |
| Programmed cell death protein 6; AltName: | calcium ion binding | calcium-dependent cysteine-type endopeptidase activity | | 0 | 0 | 0 |
| Uncharacterized protein OS=Mus musculus GN=Trim2 PE=4 SV=1 |  |  | | 0 | 0 | 0 |
| Protein FAM114A2. |  |  | | 0 | 0 | 0 |
| Heme oxygenase 2; HO-2. | metal ion binding | heme oxygenase (decyclizing) activity | | 0 | 0 | 0 |
| Similar to transmembrane 9 superfamily member 2 (Tm9sf2) OS=Mus musculus GN=Gm364 PE=4 SV=1 |  |  | | 0 | 0 | 0 |
| Uncharacterized protein (Fragment) OS=Mus musculus GN=Wdr61 PE=4 SV=1 |  |  | | 0 | 0 | 0 |
| F-actin-capping protein subunit alpha-1; AltName: | protein binding | protein binding | | 0 | 0 | 0 |
| Actin-related protein 10; Actin-related | protein binding | protein binding | | 0 | 0 | 0 |
| Uncharacterized protein OS=Mus musculus GN=Grinl1a PE=4 SV=1 |  |  | | 0 | 0 | 0 |
| Argininosuccinate synthase; AltName: | ATP binding | argininosuccinate synthase activity | | 1 | 0 | 16 |
| Myb-binding protein 1A; Myb-binding | protein binding | DNA-directed DNA polymerase activity | | 3 | 0 | 7 |
| S-methyl-5'-thioadenosine phosphorylase; AltName: |  | S-methyl-5-thioadenosine phosphorylase activity | | 1 | 0 | 3 |
| Peptidyl-prolyl cis-trans isomerase FKBP4; | protein binding | peptidyl-prolyl cis-trans isomerase activity | | 3 | 0 | 3 |
| 14-3-3 protein eta. | protein binding | sodium channel regulator activity | | 2 | 2 | 13 |
| Beta-actin-like protein 2 OS=Mus musculus GN=Actbl2 PE=1 SV=1 | ATP binding | ATP binding | | 3 | 2 | 8 |
| Galectin-1; Gal-1; 14 kDa lectin; | protein binding | signal transducer activity | | 0 | 1 | 0 |
| Splicing factor, proline- and glutamine-rich; | protein binding | nucleotide binding | | 0 | 1 | 4 |
| Keratin, type II cytoskeletal 1b; AltName: |  | structural molecule activity | | 0 | 1 | 2 |
| UV excision repair protein RAD23 homolog B; | damaged DNA binding | protein binding | | 0 | 1 | 1 |
| Thioredoxin; Trx; ATL-derived | protein binding | protein binding | | 0 | 1 | 0 |
| Isoform 2 of Nuclear protein localization protein 4 homolog OS=Mus musculus GN=Nploc4 |  |  | | 0 | 1 | 0 |
| Heterogeneous nuclear ribonucleoprotein A1; | RNA binding | nucleotide binding | | 0 | 0 | 10 |
| GMP synthase [glutamine-hydrolyzing]; AltName: | ATP binding | ATP binding | | 0 | 0 | 1 |
| 26S proteasome non-ATPase regulatory subunit 8; |  |  | | 0 | 0 | 0 |
| RANDOM_E9Q4F7\|E9Q4F7 |  |  | | 0 | 0 | 0 |
| DnaJ homolog subfamily A member 2; AltName: | metal ion binding | ATP binding | | 0 | 0 | 0 |
| Novel protein OS=Mus musculus GN=Gm5936 PE=4 SV=1 |  |  | | 0 | 0 | 0 |
| 26S proteasome non-ATPase regulatory subunit 7; |  |  | | 0 | 0 | 0 |
| Complement component 1, q subcomponent binding protein OS=Mus musculus GN=C1qbp PE=2 SV=1 |  |  | | 1 | 0 | 5 |
| Isoform 2 of A-kinase anchor protein 12 OS=Mus musculus GN=Akap12 |  |  | | 1 | 0 | 0 |
| Keratin, type II cytoskeletal 6A; AltName: |  | structural molecule activity | | 1 | 0 | 3 |
| Procollagen-lysine,2-oxoglutarate 5-dioxygenase 3; | L-ascorbic acid binding | iron ion binding | | 1 | 0 | 0 |
| Obg-like ATPase 1; GTP-binding protein | ribosome binding | ATPase activity | | 3 | 0 | 1 |
| Histone H3.2. | DNA binding | DNA binding | | 0 | 0 | 58 |
| Peripherin. | protein binding | structural molecule activity | | 0 | 0 | 45 |
| Histone H2A (Fragment) OS=Mus musculus GN=Hist1h2al PE=3 SV=1 |  |  | | 0 | 0 | 31 |
| Uncharacterized protein OS=Mus musculus GN=Vim PE=3 SV=1 |  |  | | 0 | 0 | 17 |
| Phosphatidylethanolamine-binding protein 1; | lipid binding | ATP binding | | 0 | 0 | 14 |
| Uncharacterized protein OS=Mus musculus GN=2810422J05Rik PE=4 SV=1 |  |  | | 0 | 0 | 9 |
| Histone H3.3. | DNA binding | DNA binding | | 0 | 0 | 3 |
| Heterogeneous nuclear ribonucleoprotein U, isoform CRA_b OS=Mus musculus GN=Hnrnpu PE=4 SV=1 |  |  | | 0 | 0 | 7 |
| Heterogeneous nuclear ribonucleoproteins A2/B1; | RNA binding | nucleotide binding | | 0 | 0 | 8 |
| Cytoplasmic dynein 1 heavy chain 1; AltName: | ATP binding | microtubule motor activity | | 0 | 0 | 4 |
| Macrophage migration inhibitory factor; MIF; | cytokine activity | chemoattractant activity | | 0 | 0 | 7 |
| 60S ribosomal protein L4. | protein binding | protein binding | | 0 | 0 | 5 |
| Adenine phosphoribosyltransferase; APRT. | AMP binding | adenine phosphoribosyltransferase activity | | 0 | 0 | 4 |
| Aldose reductase; AR; Aldehyde |  | aldehyde reductase activity | | 0 | 0 | 6 |
| Histone H2A.Z; H2A/z. | DNA binding | DNA binding | | 0 | 0 | 7 |
| Heterogeneous nuclear ribonucleoprotein A3; | RNA binding | nucleotide binding | | 0 | 0 | 5 |
| Heterogeneous nuclear ribonucleoprotein L; | RNA binding | nucleotide binding | | 0 | 0 | 4 |
| 40S ribosomal protein S9. | rRNA binding | translation regulator activity | | 0 | 0 | 2 |
| Ubiquitin-conjugating enzyme E2 L3; AltName: | transcription coactivator activity | transcription coactivator activity | | 0 | 0 | 4 |
| 60S ribosomal protein L27a; L29. |  | structural constituent of ribosome | | 0 | 0 | 5 |
| Phosphoribosyl pyrophosphate synthase-associated | magnesium ion binding | magnesium ion binding | | 0 | 0 | 3 |
| Histone H3.1. | DNA binding | protein binding | | 0 | 0 | 4 |
| 40S ribosomal protein S6; AltName: | protein binding | protein binding | | 0 | 0 | 2 |
| Spectrin alpha chain, non-erythrocytic 1; AltName: | protein heterodimerization activity | calcium ion binding | | 0 | 0 | 4 |
| Protein-L-isoaspartate(D-aspartate) |  | protein-L-isoaspartate (D-aspartate) O-methyltransferase activity | | 0 | 0 | 4 |
| Lipid phosphate phosphohydrolase 1; 35 |  | phosphatidate phosphatase activity | | 0 | 0 | 4 |
| TAR DNA-binding protein 43; TDP-43. | mRNA 3'-UTR binding | nucleotide binding | | 0 | 0 | 3 |
| Lamin-B1; Flags: Precursor. | JUN kinase binding | structural molecule activity | | 0 | 0 | 3 |
| Adenylyl cyclase-associated protein 1; CAP 1. | protein binding | protein binding | | 0 | 0 | 4 |
| 60S ribosomal protein L8. | rRNA binding | rRNA binding | | 0 | 0 | 3 |
| Uncharacterized protein OS=Mus musculus GN=Prmt1 PE=4 SV=1 |  |  | | 0 | 0 | 1 |
| Calcyclin-binding protein; CacyBP; AltName: | protein binding | protein binding | | 0 | 0 | 2 |
| Uncharacterized protein OS=Mus musculus GN=Ank1 PE=4 SV=1 |  |  | | 0 | 0 | 0 |
| Phosphoribosylformylglycinamidine synthase; | ATP binding | ATP binding | | 0 | 0 | 2 |
| Isoform 2 of Heterogeneous nuclear ribonucleoprotein F OS=Mus musculus GN=Hnrnpf |  |  | | 0 | 0 | 5 |
| Coactosin-like protein. | protein binding | protein binding | | 0 | 0 | 1 |
| ATP-dependent RNA helicase A; RHA; AltName: | ATP binding | ATP-dependent helicase activity | | 0 | 0 | 3 |
| Aspartate aminotransferase, cytoplasmic; | carboxylic acid binding | aspartate transaminase activity | | 0 | 0 | 2 |
| NADPH--cytochrome P450 reductase; CPR; | FMN binding | NADP binding | | 0 | 0 | 3 |
| Peroxiredoxin-4 OS=Mus musculus GN=Prdx4 PE=1 SV=1 | protein homodimerization activity | protein homodimerization activity | | 0 | 0 | 6 |
| Eukaryotic translation initiation factor 3 subunit M; | translation initiation factor binding | translation initiation factor activity | | 0 | 0 | 2 |
| N-acetyltransferase 10. | ATP binding | ATP binding | | 0 | 0 | 2 |
| Translationally-controlled tumor protein; TCTP; | transcription factor binding | calcium ion binding | | 0 | 0 | 2 |
| Proteasome subunit alpha type-4; AltName: |  | threonine-type endopeptidase activity | | 0 | 0 | 2 |
| Endoplasmic reticulum resident protein 29; |  |  | | 0 | 0 | 2 |
| Keratin, type II cytoskeletal 2 oral; AltName: |  | structural molecule activity | | 0 | 0 | 2 |
| Lupus La protein homolog; La | RNA binding | nucleotide binding | | 0 | 0 | 1 |
| Eukaryotic translation initiation factor 3 subunit A; | translation initiation factor activity | translation initiation factor activity | | 0 | 0 | 1 |
| COP9 signalosome complex subunit 1; SGN1; | protein binding | protein binding | | 0 | 0 | 1 |
| Spectrin beta chain, non-erythrocytic 1; AltName: | protein binding | structural constituent of cytoskeleton | | 0 | 0 | 1 |
| Eukaryotic translation initiation factor 4H; | translation initiation factor activity | translation initiation factor activity | | 0 | 0 | 1 |
| 26S proteasome non-ATPase regulatory subunit 3; | protein binding | enzyme regulator activity | | 0 | 0 | 2 |
| Tpm1 protein OS=Mus musculus GN=Tpm1 PE=2 SV=1 |  |  | | 0 | 0 | 2 |
| 5'(3')-deoxyribonucleotidase, cytosolic type; | metal ion binding | pyrimidine nucleotide binding | | 0 | 0 | 2 |
| Cytochrome b5 type B; Cytochrome b5 | metal ion binding | enzyme activator activity | | 0 | 0 | 2 |
| Uncharacterized protein OS=Mus musculus GN=Synj2 PE=4 SV=1 |  |  | | 0 | 0 | 1 |
| A kinase (PRKA) anchor protein 14 OS=Mus musculus GN=Akap14 PE=2 SV=1 |  |  | | 0 | 0 | 1 |
| Isoleucine--tRNA ligase, cytoplasmic; AltName: | ATP binding | ATP binding | | 0 | 0 | 1 |
| E9QA37\|E9QA37 |  |  | | 0 | 0 | 0 |
| Xaa-Pro aminopeptidase 1; AltName: | manganese ion binding | manganese ion binding | | 0 | 0 | 1 |
| Adenylosuccinate lyase; ASL; AltName: |  | adenylosuccinate lyase activity | | 0 | 0 | 2 |
| Isoform 2 of AP-2 complex subunit beta OS=Mus musculus GN=Ap2b1 |  |  | | 0 | 0 | 5 |
| Proteasome activator complex subunit 2; AltName: |  |  | | 0 | 0 | 2 |
| Nucleoside diphosphate kinase OS=Mus musculus GN=Nme2 PE=3 SV=1 |  |  | | 0 | 0 | 7 |
| Proteasome subunit beta type-1; AltName: |  | threonine-type endopeptidase activity | | 0 | 0 | 2 |
| Isocitrate dehydrogenase [NADP] cytoplasmic; | NADP binding | isocitrate dehydrogenase (NADP+) activity | | 0 | 0 | 2 |
| ELAV-like protein 1; Elav-like generic | protein binding | nucleotide binding | | 0 | 0 | 2 |
| Fragile X mental retardation syndrome-related protein | mRNA 3'-UTR binding | protein binding | | 0 | 0 | 2 |
| Cleavage and polyadenylation specificity factor | mRNA binding | nucleotide binding | | 0 | 0 | 2 |
| Synaptic vesicle membrane protein VAT-1 homolog-like. | zinc ion binding | oxidoreductase activity | | 0 | 0 | 2 |
| Activator of 90 kDa heat shock protein ATPase homolog |  | ATPase activator activity | | 0 | 0 | 2 |
| ATP-dependent RNA helicase DDX1; DEAD | protein binding | transcription cofactor activity | | 0 | 0 | 2 |
| Lactoylglutathione lyase; AltName: | zinc ion binding | lactoylglutathione lyase activity | | 0 | 0 | 2 |
| Translin-associated protein X; AltName: | metal ion binding | sequence-specific DNA binding | | 0 | 0 | 2 |
| Proteasome subunit beta type-3; AltName: |  | threonine-type endopeptidase activity | | 0 | 0 | 2 |
| Matrin-3. | RNA binding | zinc ion binding | | 0 | 0 | 2 |
| Ubiquitin carboxyl-terminal hydrolase isozyme L4; |  | ubiquitin-specific protease activity | | 0 | 0 | 2 |
| DnaJ homolog subfamily C member 8. |  |  | | 0 | 0 | 1 |
| Isoform 2 of Cytosol aminopeptidase OS=Mus musculus GN=Lap3 |  |  | | 0 | 0 | 1 |
| Annexin A5; Anchorin CII; AltName: | calcium-dependent phospholipid binding | protein binding | | 0 | 0 | 2 |
| ATPase, Cu++ transporting, alpha polypeptide OS=Mus musculus GN=Atp7a PE=3 SV=1 |  |  | | 0 | 0 | 0 |
| Proteasome subunit beta type-4; Low | lipopolysaccharide binding | threonine-type endopeptidase activity | | 0 | 0 | 1 |
| 3-hydroxyacyl-CoA dehydrogenase type-2; AltName: |  | 3-hydroxy-2-methylbutyryl-CoA dehydrogenase activity | | 0 | 0 | 2 |
| cAMP-dependent protein kinase catalytic subunit | protein binding | ATP binding | | 0 | 0 | 2 |
| Histone H2AX; H2a/x; Histone | damaged DNA binding | protein binding | | 0 | 0 | 8 |
| Histone H1.5; H1 VAR.5; AltName: | DNA binding | protein binding | | 0 | 0 | 2 |
| Glutamine--fructose-6-phosphate aminotransferase | carbohydrate binding | glutamine-fructose-6-phosphate transaminase (isomerizing) activity | | 0 | 0 | 1 |
| Hydroxyacyl-coenzyme A dehydrogenase, mitochondrial; |  | 3-hydroxyacyl-CoA dehydrogenase activity | | 0 | 0 | 2 |
| Eukaryotic translation initiation factor 2 subunit 3, | GTP binding | translation factor activity, nucleic acid binding | | 0 | 0 | 1 |
| ATPase family AAA domain-containing protein 1; | ATP binding | ATPase activity | | 0 | 0 | 2 |
| Peptidyl-prolyl cis-trans isomerase FKBP1A; | protein binding | peptidyl-prolyl cis-trans isomerase activity | | 0 | 0 | 1 |
| Translin; Component 3 of promoter of | protein binding | endonuclease activity | | 0 | 0 | 1 |
| Vesicle-associated membrane protein-associated |  | structural molecule activity | | 0 | 0 | 1 |
| Protein phosphatase 1 regulatory subunit 14B; |  | protein phosphatase inhibitor activity | | 0 | 0 | 1 |
| Chloride intracellular channel protein 4; | protein binding | protein binding | | 0 | 0 | 1 |
| E9Q7G0\|E9Q7G0 |  |  | | 0 | 0 | 1 |
| Isoform 2 of STE20-like serine/threonine-protein kinase OS=Mus musculus GN=Slk |  |  | | 0 | 0 | 1 |
| Eukaryotic translation initiation factor 4B; | translation initiation factor activity | translation initiation factor activity | | 0 | 0 | 1 |
| Casein kinase 2, alpha 1 polypeptide (Fragment) OS=Mus musculus GN=Csnk2a1 PE=3 SV=1 |  |  | | 0 | 0 | 0 |
| Uncharacterized protein OS=Mus musculus GN=Syncrip PE=4 SV=1 |  |  | | 0 | 0 | 0 |
| Uncharacterized protein OS=Mus musculus GN=Uba6 PE=4 SV=1 |  |  | | 0 | 0 | 1 |
| Uncharacterized protein (Fragment) OS=Mus musculus GN=Fus PE=4 SV=1 |  |  | | 0 | 0 | 1 |
| Enhancer of rudimentary homolog; Mer. |  |  | | 0 | 0 | 1 |
| Isoform 2 of Drebrin-like protein OS=Mus musculus GN=Dbnl |  |  | | 0 | 0 | 1 |
| Saccharopine dehydrogenase-like oxidoreductase. |  | oxidoreductase activity | | 0 | 0 | 1 |
| Nuclear pore complex protein Nup155; AltName: |  | structural constituent of nuclear pore | | 0 | 0 | 1 |
| Dynamin-like 120 kDa protein, mitochondrial; AltName: | GTP binding | protein binding | | 0 | 0 | 1 |
| Aquaporin-11; AQP-11. |  | water channel activity | | 0 | 0 | 0 |
| Uncharacterized protein OS=Mus musculus GN=Otub1 PE=4 SV=1 |  |  | | 0 | 0 | 1 |
| P61027\|RAB10 | GTP binding | protein binding | | 0 | 0 | 2 |
| 60S ribosomal protein L18a. |  | structural constituent of ribosome | | 0 | 0 | 1 |
| Deoxynucleoside triphosphate triphosphohydrolase | dGTP binding | phosphoric diester hydrolase activity | | 0 | 0 | 1 |
| Uncharacterized protein OS=Mus musculus GN=Smarcc1 PE=4 SV=1 |  |  | | 0 | 0 | 1 |
| Lysosome membrane protein 2; 85 kDa | enzyme binding | receptor activity | | 0 | 0 | 1 |
| Splicing factor 3B subunit 1; AltName: | chromatin binding | protein binding | | 0 | 0 | 1 |
| 60S ribosomal protein L37a. | metal ion binding | metal ion binding | | 0 | 0 | 1 |
| Ubiquitin-conjugating enzyme E2 D3; AltName: | ATP binding | ubiquitin-protein ligase activity | | 0 | 0 | 1 |
| Chromodomain helicase DNA binding protein 3 OS=Mus musculus GN=Chd3 PE=4 SV=1 |  |  | | 0 | 0 | 0 |
| Eukaryotic translation initiation factor 5B OS=Mus musculus GN=Eif5b PE=1 SV=2 | GTP binding | translation initiation factor activity | | 0 | 0 | 0 |
| Prefoldin subunit 5; C-Myc-binding |  |  | | 0 | 0 | 1 |
| DNA topoisomerase 2-alpha; DNA | ATP binding | DNA topoisomerase (ATP-hydrolyzing) activity | | 0 | 0 | 1 |
| Cyclin-K. |  | RNA polymerase subunit kinase activity | | 0 | 0 | 1 |
| Suppressor of G2 allele of SKP1 homolog. |  |  | | 0 | 0 | 1 |
| Serine/threonine-protein phosphatase PP1-beta catalytic subunit OS=Mus musculus GN=Ppp1cb PE=1 SV=3 | protein binding | metal ion binding | | 0 | 0 | 2 |
| U6 snRNA-associated Sm-like protein LSm2; AltName: | protein binding | RNA binding | | 0 | 0 | 1 |
| Regulator of microtubule dynamics protein 3; |  |  | | 0 | 0 | 1 |
| RWD domain-containing protein 1; DRG |  |  | | 0 | 0 | 1 |
| Delta-aminolevulinic acid dehydratase; ALADH; | identical protein binding | porphobilinogen synthase activity | | 0 | 0 | 1 |
| Arsenite methyltransferase; AltName: |  | arsenite methyltransferase activity | | 0 | 0 | 0 |
| Pre-mRNA-processing-splicing factor 8; AltName: | U5 snRNA binding | U5 snRNA binding | | 0 | 0 | 0 |
| Alpha actinin 1a OS=Mus musculus GN=Actn1 PE=2 SV=1 |  |  | | 0 | 0 | 1 |
| 1-phosphatidylinositol 4,5-bisphosphate | calcium ion binding | phosphoinositide phospholipase C activity | | 0 | 0 | 0 |
| Isochorismatase domain-containing protein 1. |  | catalytic activity | | 0 | 0 | 1 |
| Elongation factor Ts, mitochondrial; EF-Ts; | translation elongation factor activity | translation elongation factor activity | | 0 | 0 | 1 |
| 60S ribosomal protein L13a; AltName: | mRNA binding | mRNA binding | | 0 | 0 | 1 |
| 60S ribosomal protein L3; J1 protein. |  | structural constituent of ribosome | | 0 | 0 | 1 |
| 3-ketoacyl-CoA thiolase A, peroxisomal; AltName: |  | acetyl-CoA C-acetyltransferase activity | | 0 | 0 | 1 |
| COP9 signalosome complex subunit 8; SGN8; | protein binding | protein binding | | 0 | 0 | 1 |
| Signal recognition particle receptor subunit beta; | GTP binding | GTP binding | | 0 | 0 | 1 |
| Uncharacterized protein OS=Mus musculus GN=Snrpe PE=4 SV=1 |  |  | | 0 | 0 | 0 |
| Voltage-dependent anion-selective channel protein 3 OS=Mus musculus GN=Vdac3 PE=1 SV=1 | nucleotide binding | voltage-gated anion channel activity | | 0 | 0 | 0 |
| Pyruvate dehydrogenase protein X component, |  | transferase activity, transferring acyl groups | | 0 | 0 | 0 |
| Bifunctional methylenetetrahydrofolate | phosphate binding | magnesium ion binding | | 0 | 0 | 0 |
| Uncharacterized protein OS=Mus musculus GN=Gm4511 PE=4 SV=1 |  |  | | 0 | 0 | 1 |
| Transcription elongation factor B polypeptide 2; | protein binding | protein binding | | 0 | 0 | 1 |
| Heterogeneous nuclear ribonucleoprotein R OS=Mus musculus GN=Hnrnpr PE=2 SV=1 |  |  | | 0 | 0 | 0 |
| Proteasome subunit alpha type-5; AltName: |  | threonine-type endopeptidase activity | | 0 | 0 | 1 |
| Cytochrome c oxidase subunit 2; AltName: | copper ion binding | cytochrome-c oxidase activity | | 0 | 0 | 1 |
| Exocyst complex component 1; Exocyst |  |  | | 0 | 0 | 1 |
| Phosphoglucomutase-1; PGM 1; AltName: | magnesium ion binding | magnesium ion binding | | 0 | 0 | 0 |
| Uncharacterized protein OS=Mus musculus GN=Fen1 PE=3 SV=1 |  |  | | 0 | 0 | 0 |
| Q60668-3\|HNRPD |  |  | | 0 | 0 | 1 |
| Calcium-binding mitochondrial carrier protein | calcium ion binding | L-aspartate transmembrane transporter activity | | 0 | 0 | 0 |
| Splicing factor 3A subunit 1; SF3a120. | RNA binding | RNA binding | | 0 | 0 | 0 |
| Cation-dependent mannose-6-phosphate receptor; | mannose binding | mannose transmembrane transporter activity | | 0 | 0 | 0 |
| Aminopeptidase N; AP-N; mAPN; AltName: | zinc ion binding | aminopeptidase activity | | 0 | 0 | 0 |
| RANDOM_Q9D9U9\|Q9D9U9 |  |  | | 0 | 0 | 0 |
| Sorting nexin 1 OS=Mus musculus GN=Snx1 PE=2 SV=1 |  |  | | 0 | 0 | 0 |
| Glyceraldehyde-3-phosphate dehydrogenase OS=Mus musculus GN=Gm10358 PE=3 SV=1 |  |  | | 0 | 0 | 50 |
| Coronin-1C; Coronin-3. | actin filament binding | actin filament binding | | 0 | 0 | 0 |
| Ipo4 protein OS=Mus musculus GN=Ipo4 PE=2 SV=1 |  |  | | 0 | 0 | 0 |
| Interleukin enhancer-binding factor 2; AltName: | ATP binding | transferase activity | | 0 | 0 | 0 |
| Elongator complex protein 1; ELP1; AltName: | DNA binding | DNA binding | | 0 | 0 | 0 |
| A2A9R4\|A2A9R4 |  |  | | 0 | 0 | 1 |
| Protein phosphatase 2, regulatory subunit B, delta isoform, isoform CRA_d OS=Mus musculus GN=Ppp2r2d PE=4 SV=2 |  |  | | 0 | 0 | 0 |
| Uncharacterized protein OS=Mus musculus GN=Gcn1l1 PE=4 SV=1 |  |  | | 0 | 0 | 1 |
| Non-POU domain-containing octamer-binding protein; | protein binding | nucleotide binding | | 0 | 0 | 10 |
| 60S ribosomal protein L15. |  | structural constituent of ribosome | | 0 | 0 | 5 |
| Fascin; Singed-like protein. | protein binding | drug binding | | 0 | 0 | 5 |
| Protein RCC2. |  |  | | 0 | 0 | 2 |
| Protein FAM208A. |  |  | | 0 | 0 | 0 |
| Alcohol dehydrogenase class-3; Alcohol | protein homodimerization activity | alcohol dehydrogenase activity | | 0 | 0 | 0 |
| CD9 antigen; AltName: CD_antigen=CD9. | protein binding | protein binding | | 0 | 0 | 0 |
| 60S ribosomal protein L18. |  | structural constituent of ribosome | | 1 | 0 | 3 |
| Transcription factor BTF3; Nascent |  |  | | 1 | 0 | 3 |
| High mobility group protein B1; High | protein binding | transcription factor activity | | 1 | 0 | 1 |
| Lamin-B receptor; Integral nuclear | DNA binding | DNA binding | | 1 | 0 | 0 |
| SAP domain-containing ribonucleoprotein; AltName: | RNA binding | RNA binding | | 1 | 0 | 0 |
| Cysteinyl-tRNA synthetase, cytoplasmic OS=Mus musculus GN=Cars PE=1 SV=2 | metal ion binding | ATP binding | | 1 | 0 | 0 |
| Proteolipid protein 2. | chemokine binding | chemokine binding | | 1 | 0 | 0 |
| Glucose-6-phosphate isomerase; GPI; AltName: | protein binding | glucose-6-phosphate isomerase activity | | 1 | 0 | 11 |
| 60S ribosomal protein L7. | protein homodimerization activity | mRNA binding | | 0 | 0 | 6 |
| Cytochrome c, somatic. | heme binding | electron carrier activity | | 0 | 0 | 3 |
| DNA-(apurinic or apyrimidinic site) lyase; AltName: | transcription coactivator activity | transcription coactivator activity | | 0 | 0 | 2 |
| Uncharacterized protein OS=Mus musculus GN=Ino80 PE=4 SV=2 |  |  | | 0 | 0 | 0 |
| Peroxisomal multifunctional enzyme type 2 OS=Mus musculus GN=Hsd17b4 PE=1 SV=3 | sterol binding | sterol binding | | 0 | 0 | 0 |
| Isoform E of Leptin receptor OS=Mus musculus GN=Lepr |  |  | | 0 | 0 | 0 |
| Lysosome-associated membrane glycoprotein 1; | protein binding | protein domain specific binding | | 0 | 0 | 0 |
| Actin-related protein 2/3 complex subunit 4; AltName: |  |  | | 0 | 0 | 0 |
| Hematological and neurological expressed 1 protein; |  |  | | 0 | 0 | 0 |
| FAS-associated death domain protein; AltName: | protein binding | protein binding | | 0 | 0 | 0 |
| 39S ribosomal protein L1, mitochondrial; L1mt; | RNA binding | RNA binding | | 0 | 0 | 0 |
| Thioredoxin reductase 1, cytoplasmic; TR; | FAD binding | thioredoxin-disulfide reductase activity | | 0 | 0 | 0 |
| Profilin-1; Profilin I. | phosphatidylinositol-4,5-bisphosphate binding | Rho GTPase binding | | 1 | 0 | 7 |
| Anamorsin; Cytokine-induced apoptosis | metal ion binding | electron carrier activity | | 1 | 0 | 0 |
| Tyrosine--tRNA ligase, cytoplasmic; AltName: | tRNA binding | tyrosine-tRNA ligase activity | | 1 | 0 | 1 |
| 40S ribosomal protein S8. |  | structural constituent of ribosome | | 1 | 0 | 8 |
| ATP-dependent RNA helicase DDX3X; AltName: | protein binding | ATPase activity | | 1 | 0 | 4 |
| Uncharacterized protein (Fragment) OS=Mus musculus GN=Tln1 PE=4 SV=1 |  |  | | 1 | 0 | 1 |
| 60S ribosomal protein L19. |  | structural constituent of ribosome | | 1 | 0 | 2 |
| Isoform 2 of tRNA (cytosine(34)-C(5))-methyltransferase OS=Mus musculus GN=Nsun2 |  |  | | 1 | 0 | 0 |
| 60S ribosomal protein L9. | rRNA binding | rRNA binding | | 1 | 0 | 1 |
| MCG11809 OS=Mus musculus GN=Gm10288 PE=3 SV=1 |  |  | | 1 | 0 | 0 |
| S-adenosylmethionine synthase isoform type-2; | metal ion binding | methionine adenosyltransferase activity | | 1 | 0 | 0 |
| Mannose-1-phosphate guanyltransferase beta; AltName: | GTP binding | GTP binding | | 1 | 0 | 0 |
| Ezrin; Cytovillin; AltName: | protein binding | cell adhesion molecule binding | | 1 | 0 | 0 |
| COP9 signalosome complex subunit 4; SGN4; | protein binding | protein binding | | 1 | 0 | 0 |
| Leukotriene A-4 hydrolase; LTA-4 hydrolase; | protein binding | leukotriene-A4 hydrolase activity | | 1 | 0 | 0 |
| Isocitrate dehydrogenase [NADP], mitochondrial; | magnesium ion binding | isocitrate dehydrogenase (NADP+) activity | | 1 | 0 | 0 |
| Protein CDV3; Carnitine |  |  | | 1 | 0 | 0 |
| Prolyl 4-hydroxylase subunit alpha-2; 4-PH | L-ascorbic acid binding | procollagen-proline 4-dioxygenase activity | | 1 | 0 | 0 |
| Q61029-2\|LAP2B |  |  | | 1 | 0 | 0 |
| Fructose-2,6-bisphosphatase TIGAR; AltName: |  | fructose-2,6-bisphosphate 2-phosphatase activity | | 1 | 0 | 0 |
| UTP--glucose-1-phosphate uridylyltransferase; | pyrimidine ribonucleotide binding | UTP:glucose-1-phosphate uridylyltransferase activity | | 1 | 0 | 0 |
| NSFL1 cofactor p47; p97 cofactor p47. | lipid binding | protein binding | | 1 | 0 | 0 |
| ADP-ribosylation factor-like protein 3. | GTP binding | metal ion binding | | 1 | 0 | 0 |
| Prolyl endopeptidase; PE; AltName: |  | serine-type exopeptidase activity | | 1 | 0 | 2 |
| COP9 signalosome complex subunit 2; SGN2; | protein binding | transcription corepressor activity | | 1 | 0 | 1 |
| Peptidyl-prolyl cis-trans isomerase D; PPIase | peptide binding | peptidyl-prolyl cis-trans isomerase activity | | 1 | 0 | 0 |
| AP-2 complex subunit alpha-2; 100 kDa | lipid binding | protein transporter activity | | 1 | 0 | 0 |
| Annexin A6; 67 kDa calelectrin; | calcium-dependent phospholipid binding | calcium ion binding | | 1 | 0 | 6 |
| 60S ribosomal protein L10; Protein QM |  | structural constituent of ribosome | | 1 | 0 | 3 |
| Isoform 3 of Programmed cell death 6-interacting protein OS=Mus musculus GN=Pdcd6ip |  |  | | 1 | 0 | 1 |
| Radixin; ESP10. | protein binding | protein binding | | 1 | 0 | 0 |
| Dihydrofolate reductase. | mRNA binding | dihydrofolate reductase activity | | 1 | 0 | 0 |
| MARCKS-related protein; Brain protein |  |  | | 1 | 0 | 0 |
| BRO1 domain-containing protein BROX; AltName: |  |  | | 1 | 0 | 0 |
| Ubiquitin-conjugating enzyme E2 variant 1; | protein binding | ubiquitin-protein ligase activity | | 1 | 0 | 0 |
| Transaldolase. | monosaccharide binding | transaldolase activity | | 2 | 0 | 3 |
| Ubiquitin carboxyl-terminal hydrolase isozyme L1; | ubiquitin binding | ubiquitin binding | | 2 | 0 | 17 |
| Isoform 2 of Rab GDP dissociation inhibitor beta OS=Mus musculus GN=Gdi2 |  |  | | 2 | 0 | 9 |
| Ubiquitin-conjugating enzyme E2 N; AltName: | ATP binding | ubiquitin binding | | 2 | 0 | 2 |
| Nucleoside diphosphate kinase A; NDK A; | ATP binding | deoxyribonuclease activity | | 3 | 0 | 7 |
| Eukaryotic translation initiation factor 6; | ribosome binding | translation initiation factor activity | | 1 | 0 | 0 |
| Cofilin-2 OS=Mus musculus GN=Cfl2 PE=1 SV=1 |  |  | | 1 | 0 | 0 |
| Glutathione S-transferase P 1; Gst P1; AltName: | JUN kinase binding | glutathione transferase activity | | 1 | 0 | 3 |
| 40S ribosomal protein S28. |  | structural constituent of ribosome | | 2 | 0 | 2 |
| Annexin A2; Annexin II; AltName: | calcium ion binding | phospholipase inhibitor activity | | 2 | 0 | 1 |
| Brain acid soluble protein 1; 22 kDa | protein binding | transcription corepressor activity | | 1 | 0 | 3 |
| Adenosylhomocysteinase; AdoHcyase; AltName: | protein binding | adenosylhomocysteinase activity | | 1 | 0 | 0 |
| 28 kDa heat- and acid-stable phosphoprotein; AltName: |  |  | | 1 | 0 | 0 |
| Thimet oligopeptidase. | metal ion binding | metalloendopeptidase activity | | 1 | 0 | 0 |
| Copper transport protein ATOX1; Metal | copper chaperone activity | copper chaperone activity | | 1 | 0 | 0 |
| Argininosuccinate lyase; ASAL; AltName: |  | argininosuccinate lyase activity | | 2 | 0 | 4 |
| Protein canopy homolog 2; AltName: |  |  | | 2 | 0 | 2 |
| Serum albumin; Flags: Precursor. | oxygen binding | zinc ion binding | | 2 | 0 | 0 |
| Eukaryotic translation initiation factor 2A; | ribosome binding | translation initiation factor activity | | 2 | 0 | 0 |
| Rho GDP-dissociation inhibitor 1; Rho GDI 1; | protein binding | GTPase activator activity | | 4 | 0 | 9 |
| TBC1 domain family member 9B OS=Mus musculus GN=Tbc1d9b PE=2 SV=1 |  |  | | 0 | 0 | 0 |
| Eukaryotic translation initiation factor 5; | GTP binding | translation initiation factor activity | | 0 | 0 | 0 |
| Isoform 2 of Leukocyte surface antigen CD47 OS=Mus musculus GN=Cd47 |  |  | | 0 | 0 | 0 |
| Ras-related C3 botulinum toxin substrate 3; AltName: | GTP binding | protein binding | | 1 | 0 | 0 |
| Polyribonucleotide nucleotidyltransferase 1, | poly(U) binding | 3'-5'-exoribonuclease activity | | 1 | 0 | 0 |
| Arginine-rich, mutated in early stage tumors, isoform CRA_b OS=Mus musculus GN=Manf PE=2 SV=1 |  |  | | 1 | 0 | 3 |
| Adenylosuccinate synthetase isozyme 2; AMPSase | GTP binding | magnesium ion binding | | 2 | 0 | 0 |
| ATP-binding cassette sub-family E member 1; AltName: | iron-sulfur cluster binding | ATPase activity | | 2 | 0 | 0 |
| Isocitrate dehydrogenase [NAD] subunit alpha, | magnesium ion binding | isocitrate dehydrogenase (NAD+) activity | | 2 | 0 | 2 |
| Transcription elongation factor A protein 1; AltName: | DNA binding | zinc ion binding | | 2 | 0 | 0 |
| Acidic leucine-rich nuclear phosphoprotein 32 family | protein binding | protein binding | | 3 | 0 | 1 |
| tRNA methyltransferase 112 homolog; AltName: |  | protein methyltransferase activity | | 1 | 0 | 0 |
| Neuromodulin; Axonal membrane protein |  |  | | 1 | 0 | 1 |
| Phosphatidylinositol transfer protein beta isoform; | phospholipid binding | phospholipid binding | | 1 | 0 | 0 |
| Methionine aminopeptidase 2; MAP 2; MetAP | metal ion binding | protein binding | | 1 | 0 | 0 |
| Actin-related protein 3; Actin-like | ATP binding | protein binding | | 2 | 0 | 1 |
| High mobility group protein B2; High | damaged DNA binding | transcription factor activity | | 2 | 0 | 0 |
| Cathepsin D; Flags: Precursor. |  | aspartic-type endopeptidase activity | | 1 | 0 | 2 |
| Uncharacterized protein OS=Mus musculus GN=Mat2b PE=4 SV=1 |  |  | | 2 | 0 | 0 |
| Rab GDP dissociation inhibitor alpha; Rab GDI |  | GTPase activator activity | | 2 | 0 | 5 |
| Eukaryotic translation initiation factor 2 subunit 1; | ribosome binding | translation initiation factor activity | | 2 | 0 | 3 |
| Protein phosphatase 1 regulatory subunit 7; AltName: |  |  | | 2 | 0 | 2 |
| Prefoldin subunit 3; Von |  |  | | 2 | 0 | 3 |
| Eukaryotic peptide chain release factor subunit 1; | translation release factor activity, codon specific | translation release factor activity, codon specific | | 2 | 0 | 2 |
| Isoform 2 of Adenylate kinase isoenzyme 1 OS=Mus musculus GN=Ak1 |  |  | | 2 | 0 | 0 |
| Nuclear migration protein nudC; Nuclear | protein binding | protein binding | | 2 | 0 | 1 |
| 60S ribosomal protein L14. |  | structural constituent of ribosome | | 2 | 0 | 0 |
| Small ubiquitin-related modifier 2; SUMO-2; | ubiquitin protein ligase binding | SUMO ligase activity | | 2 | 0 | 0 |
| Actin-related protein 2/3 complex subunit 3; AltName: |  |  | | 2 | 0 | 0 |
| Uncharacterized protein OS=Mus musculus GN=Anp32b PE=4 SV=1 |  |  | | 2 | 0 | 1 |
| Histidine triad nucleotide-binding protein 1; | nucleotide binding | hydrolase activity | | 2 | 0 | 5 |
| Dual specificity mitogen-activated protein kinase | ATP binding | receptor signaling protein tyrosine phosphatase activity | | 2 | 0 | 0 |
| Phosphoserine aminotransferase; PSAT; AltName: | pyridoxal phosphate binding | pyridoxal phosphate binding | | 2 | 0 | 1 |
| Aspartate aminotransferase, mitochondrial; | pyridoxal phosphate binding | pyridoxal phosphate binding | | 3 | 0 | 1 |
| Phosphoglycerate mutase 1; AltName: |  | phosphoglycerate mutase activity | | 3 | 0 | 12 |
| Protein disulfide-isomerase A4; AltName: |  | protein disulfide oxidoreductase activity | | 3 | 0 | 0 |
| IgE-binding protein. | nucleic acid binding | nucleic acid binding | | 3 | 0 | 4 |
| 6-phosphogluconate dehydrogenase, decarboxylating. | NADP binding | phosphogluconate dehydrogenase (decarboxylating) activity | | 3 | 0 | 2 |
| Protein DJ-1; Parkinson disease protein | protein homodimerization activity | peroxiredoxin activity | | 4 | 0 | 7 |
| Nicotinamide phosphoribosyltransferase; | drug binding | drug binding | | 3 | 0 | 1 |
| Serine--tRNA ligase, cytoplasmic; AltName: | ATP binding | serine-tRNA ligase activity | | 4 | 0 | 5 |
| Malate dehydrogenase, cytoplasmic; AltName: | NAD binding | L-malate dehydrogenase activity | | 5 | 0 | 16 |
| Stathmin; Leukemia-associated gene | protein binding | protein binding | | 4 | 0 | 0 |
| Threonine--tRNA ligase, cytoplasmic; AltName: | ATP binding | ATP binding | | 3 | 0 | 0 |
| Serine hydroxymethyltransferase OS=Mus musculus GN=Shmt2 PE=2 SV=1 |  |  | | 5 | 0 | 3 |
| Ornithine aminotransferase, mitochondrial; AltName: | pyridoxal phosphate binding | ornithine-oxo-acid transaminase activity | | 4 | 0 | 2 |
| Programmed cell death protein 5; TF-1 | DNA binding | DNA binding | | 1 | 0 | 0 |
| Cytosolic acyl coenzyme A thioester hydrolase; |  | palmitoyl-CoA hydrolase activity | | 1 | 0 | 0 |
| Actin-related protein 2; Actin-like | ATP binding | ATP binding | | 3 | 0 | 0 |
| Nascent polypeptide-associated complex subunit alpha, | TATA-binding protein binding | transcription coactivator activity | | 4 | 0 | 2 |
| Protein SET; Phosphatase 2A inhibitor | DNA binding | DNA binding | | 5 | 0 | 5 |
| Succinyl-CoA:3-ketoacid coenzyme A transferase 1, |  | 3-oxoacid CoA-transferase activity | | 5 | 0 | 0 |
| Histidine--tRNA ligase, cytoplasmic; AltName: | ATP binding | histidine-tRNA ligase activity | | 5 | 0 | 0 |
| Proliferation-associated protein 2G4; AltName: | transcription factor activity | transcription factor activity | | 6 | 0 | 4 |
| Glyceraldehyde-3-phosphate dehydrogenase OS=Mus musculus GN=Gm6316 PE=3 SV=1 |  |  | | 7 | 0 | 0 |
| 10 kDa heat shock protein, mitochondrial; | chaperone binding | ATP binding | | 5 | 0 | 1 |
| Sepiapterin reductase; SPR. | protein homodimerization activity | protein homodimerization activity | | 4 | 0 | 0 |
| Adenylate kinase 2, mitochondrial; AK 2; | ATP binding | ATP binding | | 5 | 0 | 3 |
| Prothymosin alpha; Contains: RecName: | histone binding | histone binding | | 4 | 0 | 0 |
| Isoform Cytoplasmic of Fumarate hydratase, mitochondrial OS=Mus musculus GN=Fh |  |  | | 5 | 0 | 1 |
| Heat shock protein 90kDa alpha (Cytosolic), class A member 1 (Fragment) OS=Mus musculus GN=Hsp90aa1 PE=4 SV=2 |  |  | | 6 | 0 | 0 |
| Superoxide dismutase [Cu-Zn]. | protein binding | superoxide dismutase activity | | 7 | 0 | 10 |
| Citrate synthase, mitochondrial; AltName: |  | citrate (Si)-synthase activity | | 7 | 0 | 10 |
| Staphylococcal nuclease domain-containing protein 1; | nucleic acid binding | nuclease activity | | 6 | 0 | 1 |
| Guanine nucleotide-binding protein subunit | protein binding | receptor activity | | 10 | 0 | 9 |
| Bifunctional purine biosynthesis protein PURH; | protein homodimerization activity | protein homodimerization activity | | 8 | 0 | 3 |
| Phosphoglycerate kinase 1. | ATP binding | ATP binding | | 11 | 0 | 8 |
| Calreticulin; CRP55; AltName: | mRNA binding | carbohydrate binding | | 10 | 0 | 12 |
| Isoform Short of 14-3-3 protein beta/alpha OS=Mus musculus GN=Ywhab |  |  | | 11 | 0 | 17 |
| Aconitate hydratase, mitochondrial; Aconitase; | iron ion binding | aconitate hydratase activity | | 13 | 0 | 9 |
| Protein disulfide-isomerase A3; 58 kDa | protein binding | protein disulfide oxidoreductase activity | | 21 | 0 | 25 |
| Malate dehydrogenase, mitochondrial; Flags: |  | malate dehydrogenase activity | | 23 | 0 | 34 |
