# Supplementary material for: Synthesis and Evaluation of a Novel Adenosine-Ribose Probe for Global-Scale Profiling of Nucleoside and Nucleotide-Binding Proteins
Source: PLoS One. 2015 Feb 11;10(2):e0115644. doi: 10.1371/journal.pone.0115644 (PMC4324776; doi:10.1371/journal.pone.0115644)
Supplement: S3 Table — (DOCX) [file pone.0115644.s005.docx]

**Table S3** – Complete list of statistically over-represented GO Biological Process terms in Regeneration Wash, according to BiNGO.

| GO-ID | P-value | Corr P-value | Description | Cluster frequency | Total Frequency | Genes in test set |
| --- | --- | --- | --- | --- | --- | --- |
| 17076 | 7.74E-18 | 8.04E-16 | purine nucleotide binding | 56.10% | 6.13% | P58252\|P42932\|P56480\|P20029\|P63038\|P62631\|P63017\|P08113\|P68372\|Q8BGQ7\|P80314\|P80315\|Q9CZD3\|P80318\|Q03265\|P80316\|Q9ERD7\|P10126\|Q7TSZ0\|P99024\|Q99LC5\|P11983\|P68033 |
| 166 | 9.46E-18 | 8.04E-16 | nucleotide binding | 58.54% | 7.05% | P58252\|P42932\|P56480\|P20029\|P63038\|P62631\|P63017\|P08113\|P68372\|Q8BGQ7\|P80314\|P80315\|Q61753\|Q9CZD3\|P80318\|Q03265\|P80316\|Q9ERD7\|P10126\|Q7TSZ0\|P99024\|Q99LC5\|P11983\|P68033 |
| 32555 | 6.05E-17 | 2.60E-15 | purine ribonucleotide binding | 53.66% | 5.88% | P58252\|P42932\|P56480\|P20029\|P63038\|P62631\|P63017\|P08113\|P68372\|Q8BGQ7\|P80314\|P80315\|Q9CZD3\|P80318\|Q03265\|P80316\|Q9ERD7\|P10126\|Q7TSZ0\|P99024\|P11983\|P68033 |
| 32553 | 6.12E-17 | 2.60E-15 | ribonucleotide binding | 53.66% | 5.88% | P58252\|P42932\|P56480\|P20029\|P63038\|P62631\|P63017\|P08113\|P68372\|Q8BGQ7\|P80314\|P80315\|Q9CZD3\|P80318\|Q03265\|P80316\|Q9ERD7\|P10126\|Q7TSZ0\|P99024\|P11983\|P68033 |
| 51082 | 1.25E-16 | 4.24E-15 | unfolded protein binding | 21.95% | 0.21% | P80318\|P42932\|P80316\|Q7TSZ0\|P63017\|P08113\|P11983\|P80314\|P80315 |
| 5488 | 2.42E-14 | 6.85E-13 | binding | 92.68% | 35.54% | P58252\|P42932\|P63038\|P61979\|P62631\|P63017\|P68372\|P08113\|P35700\|P80314\|P80315\|P68369\|Q60864\|P80318\|P35979\|Q03265\|Q61171\|P80316\|Q99LC5\|P68033\|P62908\|P20029\|P56480\|Q6ZQ38\|Q8BGQ7\|Q9DB20\|P60335\|Q61753\|Q9CZD3\|Q9ERD7\|P60710\|P10126\|Q7TSZ0\|P99024\|Q3U0V1\|P70168\|P11983\|P19096 |
| 30554 | 4.45E-12 | 9.49E-11 | adenyl nucleotide binding | 41.46% | 5.09% | P42932\|P56480\|P63038\|P20029\|P63017\|P08113\|Q8BGQ7\|P80314\|P80315\|Q9CZD3\|P80318\|Q03265\|P80316\|Q7TSZ0\|Q99LC5\|P11983\|P68033 |
| 5515 | 4.47E-12 | 9.49E-11 | protein binding | 68.29% | 18.59% | P42932\|P63038\|P61979\|P62631\|P63017\|P08113\|P35700\|P80314\|P80315\|Q60864\|P68369\|P80318\|P35979\|Q61171\|P80316\|P68033\|P20029\|P56480\|Q6ZQ38\|P60335\|Q9DB20\|Q9CZD3\|P60710\|P10126\|Q7TSZ0\|P99024\|P70168\|P11983 |
| 1883 | 5.57E-12 | 1.01E-10 | purine nucleoside binding | 41.46% | 5.16% | P42932\|P56480\|P63038\|P20029\|P63017\|P08113\|Q8BGQ7\|P80314\|P80315\|Q9CZD3\|P80318\|Q03265\|P80316\|Q7TSZ0\|Q99LC5\|P11983\|P68033 |
| 1882 | 5.93E-12 | 1.01E-10 | nucleoside binding | 41.46% | 5.18% | P42932\|P56480\|P63038\|P20029\|P63017\|P08113\|Q8BGQ7\|P80314\|P80315\|Q9CZD3\|P80318\|Q03265\|P80316\|Q7TSZ0\|Q99LC5\|P11983\|P68033 |
| 5524 | 2.04E-11 | 3.16E-10 | ATP binding | 39.02% | 4.75% | P42932\|P63038\|P20029\|P56480\|P63017\|P08113\|Q8BGQ7\|P80314\|P80315\|Q9CZD3\|P80318\|Q03265\|P80316\|Q7TSZ0\|P11983\|P68033 |
| 32559 | 2.70E-11 | 3.82E-10 | adenyl ribonucleotide binding | 39.02% | 4.84% | P42932\|P63038\|P20029\|P56480\|P63017\|P08113\|Q8BGQ7\|P80314\|P80315\|Q9CZD3\|P80318\|Q03265\|P80316\|Q7TSZ0\|P11983\|P68033 |
| 3924 | 3.77E-08 | 4.93E-07 | GTPase activity | 14.63% | 0.47% | P58252\|P62631\|Q9ERD7\|P10126\|P99024\|P68372 |
| 17111 | 7.95E-08 | 9.65E-07 | nucleoside-triphosphatase activity | 21.95% | 1.96% | P58252\|Q03265\|P56480\|P62631\|Q9ERD7\|P10126\|P99024\|P63017\|P68372 |
| 16462 | 1.21E-07 | 1.28E-06 | pyrophosphatase activity | 21.95% | 2.06% | P58252\|Q03265\|P56480\|P62631\|Q9ERD7\|P10126\|P99024\|P63017\|P68372 |
| 16818 | 1.27E-07 | 1.28E-06 | hydrolase activity, acting on acid anhydrides, in phosphorus-containing anhydrides | 21.95% | 2.07% | P58252\|Q03265\|P56480\|P62631\|Q9ERD7\|P10126\|P99024\|P63017\|P68372 |
| 16817 | 1.28E-07 | 1.28E-06 | hydrolase activity, acting on acid anhydrides | 21.95% | 2.07% | P58252\|Q03265\|P56480\|P62631\|Q9ERD7\|P10126\|P99024\|P63017\|P68372 |
| 46933 | 3.14E-07 | 2.96E-06 | hydrogen ion transporting ATP synthase activity, rotational mechanism | 7.32% | 0.03% | Q03265\|P56480\|Q9DB20 |
| 5525 | 5.34E-06 | 4.78E-05 | GTP binding | 14.63% | 1.09% | P58252\|P62631\|Q9ERD7\|P10126\|P99024\|P68372 |
| 51787 | 5.86E-06 | 4.98E-05 | misfolded protein binding | 4.88% | 0.01% | P20029\|P63038 |
| 19001 | 7.07E-06 | 5.46E-05 | guanyl nucleotide binding | 14.63% | 1.15% | P58252\|P62631\|Q9ERD7\|P10126\|P99024\|P68372 |
| 32561 | 7.07E-06 | 5.46E-05 | guanyl ribonucleotide binding | 14.63% | 1.15% | P58252\|P62631\|Q9ERD7\|P10126\|P99024\|P68372 |
| 3746 | 1.04E-05 | 7.69E-05 | translation elongation factor activity | 7.32% | 0.10% | P58252\|P62631\|P10126 |
| 51920 | 4.09E-05 | 2.89E-04 | peroxiredoxin activity | 4.88% | 0.02% | Q61171\|P35700 |
| 5198 | 1.33E-04 | 9.04E-04 | structural molecule activity | 12.20% | 1.21% | Q9ERD7\|P99024\|P68372\|P62908\|Q9CZX8 |
| 46961 | 1.51E-04 | 9.87E-04 | proton-transporting ATPase activity, rotational mechanism | 4.88% | 0.04% | Q03265\|P56480 |
| 15078 | 1.59E-04 | 1.00E-03 | hydrogen ion transmembrane transporter activity | 7.32% | 0.26% | Q03265\|P56480\|Q9DB20 |
| 43531 | 2.62E-04 | 1.59E-03 | ADP binding | 4.88% | 0.06% | Q03265\|P63017 |
| 8135 | 3.23E-04 | 1.89E-03 | translation factor activity, nucleic acid binding | 7.32% | 0.32% | P58252\|P62631\|P10126 |
| 19829 | 4.04E-04 | 2.29E-03 | cation-transporting ATPase activity | 4.88% | 0.07% | Q03265\|P56480 |
| 16787 | 5.21E-04 | 2.85E-03 | hydrolase activity | 24.39% | 7.20% | P58252\|Q03265\|P56480\|P62631\|Q9ERD7\|P10126\|P99024\|P63017\|P68372\|P19096 |
| 3824 | 5.71E-04 | 3.03E-03 | catalytic activity | 39.02% | 16.78% | P58252\|P56480\|P62631\|P63017\|P68372\|P35700\|Q8BGQ7\|Q9CZD3\|Q61753\|Q03265\|Q61171\|Q9ERD7\|P10126\|P99024\|P19096\|P16858 |
| 16684 | 7.75E-04 | 3.87E-03 | oxidoreductase activity, acting on peroxide as acceptor | 4.88% | 0.10% | Q61171\|P35700 |
| 4601 | 7.75E-04 | 3.87E-03 | peroxidase activity | 4.88% | 0.10% | Q61171\|P35700 |
| 15077 | 9.47E-04 | 4.60E-03 | monovalent inorganic cation transmembrane transporter activity | 7.32% | 0.47% | Q03265\|P56480\|Q9DB20 |
| 16209 | 1.40E-03 | 5.60E-03 | antioxidant activity | 4.88% | 0.13% | Q61171\|P35700 |
| 4617 | 1.42E-03 | 5.60E-03 | phosphoglycerate dehydrogenase activity | 2.44% | 0.00% | Q61753 |
| 16631 | 1.42E-03 | 5.60E-03 | enoyl-[acyl-carrier-protein] reductase activity | 2.44% | 0.00% | P19096 |
| 19171 | 1.42E-03 | 5.60E-03 | 3-hydroxyacyl-[acyl-carrier-protein] dehydratase activity | 2.44% | 0.00% | P19096 |
| 4317 | 1.42E-03 | 5.60E-03 | 3-hydroxypalmitoyl-[acyl-carrier-protein] dehydratase activity | 2.44% | 0.00% | P19096 |
| 4316 | 1.42E-03 | 5.60E-03 | 3-oxoacyl-[acyl-carrier-protein] reductase activity | 2.44% | 0.00% | P19096 |
| 4319 | 1.42E-03 | 5.60E-03 | enoyl-[acyl-carrier-protein] reductase (NADPH, B-specific) activity | 2.44% | 0.00% | P19096 |
| 4313 | 1.42E-03 | 5.60E-03 | [acyl-carrier-protein] S-acetyltransferase activity | 2.44% | 0.00% | P19096 |
| 3723 | 1.50E-03 | 5.78E-03 | RNA binding | 12.20% | 2.06% | P61979\|Q3U0V1\|Q8BGQ7\|P62908\|P60335 |
| 16875 | 1.70E-03 | 6.15E-03 | ligase activity, forming carbon-oxygen bonds | 4.88% | 0.15% | Q9CZD3\|Q8BGQ7 |
| 16876 | 1.70E-03 | 6.15E-03 | ligase activity, forming aminoacyl-tRNA and related compounds | 4.88% | 0.15% | Q9CZD3\|Q8BGQ7 |
| 4812 | 1.70E-03 | 6.15E-03 | aminoacyl-tRNA ligase activity | 4.88% | 0.15% | Q9CZD3\|Q8BGQ7 |
| 19899 | 2.11E-03 | 7.34E-03 | enzyme binding | 9.76% | 1.33% | P63038\|P60710\|Q7TSZ0\|P08113 |
| 22890 | 2.12E-03 | 7.34E-03 | inorganic cation transmembrane transporter activity | 7.32% | 0.62% | Q03265\|P56480\|Q9DB20 |
| 42623 | 2.43E-03 | 7.76E-03 | ATPase activity, coupled | 7.32% | 0.65% | Q03265\|P56480\|P63017 |
| 16491 | 2.57E-03 | 7.76E-03 | oxidoreductase activity | 12.20% | 2.34% | Q61753\|Q61171\|P35700\|P19096\|P16858 |
| 16297 | 2.83E-03 | 7.76E-03 | acyl-[acyl-carrier-protein] hydrolase activity | 2.44% | 0.01% | P19096 |
| 8494 | 2.83E-03 | 7.76E-03 | translation activator activity | 2.44% | 0.01% | P60335 |
| 16420 | 2.83E-03 | 7.76E-03 | malonyltransferase activity | 2.44% | 0.01% | P19096 |
| 16418 | 2.83E-03 | 7.76E-03 | S-acetyltransferase activity | 2.44% | 0.01% | P19096 |
| 16419 | 2.83E-03 | 7.76E-03 | S-malonyltransferase activity | 2.44% | 0.01% | P19096 |
| 4813 | 2.83E-03 | 7.76E-03 | alanine-tRNA ligase activity | 2.44% | 0.01% | Q8BGQ7 |
| 4320 | 2.83E-03 | 7.76E-03 | oleoyl-[acyl-carrier-protein] hydrolase activity | 2.44% | 0.01% | P19096 |
| 4820 | 2.83E-03 | 7.76E-03 | glycine-tRNA ligase activity | 2.44% | 0.01% | Q9CZD3 |
| 8379 | 2.83E-03 | 7.76E-03 | thioredoxin peroxidase activity | 2.44% | 0.01% | Q61171 |
| 4315 | 2.83E-03 | 7.76E-03 | 3-oxoacyl-[acyl-carrier-protein] synthase activity | 2.44% | 0.01% | P19096 |
| 4314 | 2.83E-03 | 7.76E-03 | [acyl-carrier-protein] S-malonyltransferase activity | 2.44% | 0.01% | P19096 |
| 16597 | 2.97E-03 | 8.01E-03 | amino acid binding | 4.88% | 0.20% | P19096\|Q8BGQ7 |
| 42625 | 3.61E-03 | 9.60E-03 | ATPase activity, coupled to transmembrane movement of ions | 4.88% | 0.22% | Q03265\|P56480 |
| 16887 | 4.12E-03 | 1.08E-02 | ATPase activity | 7.32% | 0.79% | Q03265\|P56480\|P63017 |
| 4365 | 4.24E-03 | 1.08E-02 | glyceraldehyde-3-phosphate dehydrogenase (phosphorylating) activity | 2.44% | 0.01% | P16858 |
| 8943 | 4.24E-03 | 1.08E-02 | glyceraldehyde-3-phosphate dehydrogenase activity | 2.44% | 0.01% | P16858 |
| 48037 | 4.32E-03 | 1.08E-02 | cofactor binding | 7.32% | 0.80% | Q61753\|Q99LC5\|P19096 |
| 32403 | 4.37E-03 | 1.08E-02 | protein complex binding | 7.32% | 0.80% | P63038\|P99024\|Q9DB20 |
| 17025 | 5.65E-03 | 1.31E-02 | TATA-binding protein binding | 2.44% | 0.01% | Q6ZQ38 |
| 31177 | 5.65E-03 | 1.31E-02 | phosphopantetheine binding | 2.44% | 0.01% | P19096 |
| 43559 | 5.65E-03 | 1.31E-02 | insulin binding | 2.44% | 0.01% | P63038 |
| 4312 | 5.65E-03 | 1.31E-02 | fatty acid synthase activity | 2.44% | 0.01% | P19096 |
| 22892 | 5.70E-03 | 1.31E-02 | substrate-specific transporter activity | 12.20% | 2.82% | Q03265\|P56480\|P70168\|P19096\|Q9DB20 |
| 16820 | 6.78E-03 | 1.52E-02 | hydrolase activity, acting on acid anhydrides, catalyzing transmembrane movement of substances | 4.88% | 0.30% | Q03265\|P56480 |
| 42626 | 6.78E-03 | 1.52E-02 | ATPase activity, coupled to transmembrane movement of substances | 4.88% | 0.30% | Q03265\|P56480 |
| 43492 | 6.93E-03 | 1.52E-02 | ATPase activity, coupled to movement of substances | 4.88% | 0.30% | Q03265\|P56480 |
| 36 | 7.06E-03 | 1.52E-02 | acyl carrier activity | 2.44% | 0.02% | P19096 |
| 16417 | 7.06E-03 | 1.52E-02 | S-acyltransferase activity | 2.44% | 0.02% | P19096 |
| 15405 | 8.03E-03 | 1.71E-02 | P-P-bond-hydrolysis-driven transmembrane transporter activity | 4.88% | 0.33% | Q03265\|P56480 |
| 15399 | 8.20E-03 | 1.72E-02 | primary active transmembrane transporter activity | 4.88% | 0.33% | Q03265\|P56480 |
| 90079 | 8.46E-03 | 1.73E-02 | translation regulator activity, nucleic acid binding | 2.44% | 0.02% | P60335 |
| 30544 | 8.46E-03 | 1.73E-02 | Hsp70 protein binding | 2.44% | 0.02% | Q60864 |
| 43176 | 9.21E-03 | 1.86E-02 | amine binding | 4.88% | 0.35% | P19096\|Q8BGQ7 |
| 3735 | 9.73E-03 | 1.95E-02 | structural constituent of ribosome | 4.88% | 0.36% | P62908\|Q9CZX8 |
| 16616 | 1.05E-02 | 2.07E-02 | oxidoreductase activity, acting on the CH-OH group of donors, NAD or NADP as acceptor | 4.88% | 0.38% | Q61753\|P19096 |
| 16614 | 1.24E-02 | 2.42E-02 | oxidoreductase activity, acting on CH-OH group of donors | 4.88% | 0.41% | Q61753\|P19096 |
| 5215 | 1.26E-02 | 2.44E-02 | transporter activity | 12.20% | 3.43% | Q03265\|P56480\|P70168\|P19096\|Q9DB20 |
| 3730 | 1.41E-02 | 2.63E-02 | mRNA 3'-UTR binding | 2.44% | 0.03% | Q3U0V1 |
| 1530 | 1.41E-02 | 2.63E-02 | lipopolysaccharide binding | 2.44% | 0.03% | P63038 |
| 50750 | 1.41E-02 | 2.63E-02 | low-density lipoprotein receptor binding | 2.44% | 0.03% | P08113 |
| 16705 | 1.50E-02 | 2.78E-02 | oxidoreductase activity, acting on paired donors, with incorporation or reduction of molecular oxygen | 4.88% | 0.46% | Q61171\|P35700 |
| 31406 | 1.53E-02 | 2.79E-02 | carboxylic acid binding | 4.88% | 0.46% | P19096\|Q8BGQ7 |
| 70325 | 1.55E-02 | 2.80E-02 | lipoprotein receptor binding | 2.44% | 0.04% | P08113 |
| 30228 | 1.69E-02 | 3.02E-02 | lipoprotein receptor activity | 2.44% | 0.04% | P56480 |
| 3676 | 1.92E-02 | 3.34E-02 | nucleic acid binding | 19.51% | 8.41% | P58252\|P61979\|P62631\|P10126\|Q3U0V1\|Q8BGQ7\|P62908\|P60335 |
| 45182 | 1.96E-02 | 3.34E-02 | translation regulator activity | 2.44% | 0.05% | P60335 |
| 8553 | 1.96E-02 | 3.34E-02 | hydrogen-exporting ATPase activity, phosphorylative mechanism | 2.44% | 0.05% | P56480 |
| 16628 | 1.96E-02 | 3.34E-02 | oxidoreductase activity, acting on the CH-CH group of donors, NAD or NADP as acceptor | 2.44% | 0.05% | P19096 |
| 2020 | 1.96E-02 | 3.34E-02 | protease binding | 2.44% | 0.05% | P63038 |
| 8430 | 2.10E-02 | 3.50E-02 | selenium binding | 2.44% | 0.05% | Q61171 |
| 43022 | 2.10E-02 | 3.50E-02 | ribosome binding | 2.44% | 0.05% | P20029 |
| 50662 | 2.31E-02 | 3.82E-02 | coenzyme binding | 4.88% | 0.57% | Q61753\|Q99LC5 |
| 51087 | 2.66E-02 | 4.34E-02 | chaperone binding | 2.44% | 0.07% | Q60864 |
| 49 | 2.79E-02 | 4.52E-02 | tRNA binding | 2.44% | 0.07% | Q8BGQ7 |
| 8324 | 2.82E-02 | 4.53E-02 | cation transmembrane transporter activity | 7.32% | 1.61% | Q03265\|P56480\|Q9DB20 |
