# Supplementary material for: Synthesis and Evaluation of a Novel Adenosine-Ribose Probe for Global-Scale Profiling of Nucleoside and Nucleotide-Binding Proteins
Source: PLoS One. 2015 Feb 11;10(2):e0115644. doi: 10.1371/journal.pone.0115644 (PMC4324776; doi:10.1371/journal.pone.0115644)
Supplement: S4 Table — (DOCX) [file pone.0115644.s006.docx]

**Table S4** – Complete list of statistically over-represented GO Biological Process terms in PBS Wash, according to BiNGO.

| GO-ID | P-value | Corr P-value | Description | Cluster frequency | Total Freq. | Genes in test set |
| --- | --- | --- | --- | --- | --- | --- |
| 5488 | 2.74E-12 | 4.77E-10 | binding | 89.74% | 35.53% | Q9D0K2\|Q99KI0\|P32921\|P61982\|Q9CZN7\|P50580\|Q91V92\|P17742\|Q99KQ4\|Q64433\|P14152\|P08249\|P63101\|P07901\|P14211\|Q02053\|P62204\|P62827\|Q9WTP6\|P17182\|P08228\|P09411\|P54227\|P97822\|P09405\|P29758\|P40142\|Q99LX0\|Q3U2G2\|Q78PY7\|Q99PT1\|P68040\|P70670\|P17918\|P63242 |
| 3824 | 2.25E-08 | 1.96E-06 | catalytic activity | 56.41% | 16.78% | Q99KI0\|Q9D0K2\|P32921\|Q9CZN7\|Q9WTP6\|P17751\|P17182\|P08228\|Q91V92\|Q99KQ4\|P14152\|P09411\|P27773\|P08249\|Q9CWJ9\|P29758\|P40142\|Q99LX0\|Q78PY7\|Q02053\|P62827\|Q9CZU6 |
| 30060 | 1.77E-06 | 1.02E-04 | L-malate dehydrogenase activity | 5.13% | 0.01% | P08249\|P14152 |
| 5515 | 4.14E-06 | 1.80E-04 | protein binding | 51.28% | 18.59% | Q9D0K2\|P61982\|Q9CZN7\|P17182\|P08228\|P17742\|Q99KQ4\|P97822\|P54227\|P08249\|P63101\|P07901\|P14211\|P09405\|Q78PY7\|Q99PT1\|P68040\|P70670\|P17918\|P62827 |
| 46912 | 1.76E-05 | 6.13E-04 | transferase activity, transferring acyl groups, acyl groups converted into alkyl on transfer | 5.13% | 0.02% | Q91V92\|Q9CZU6 |
| 16615 | 3.69E-05 | 9.51E-04 | malate dehydrogenase activity | 5.13% | 0.02% | P08249\|P14152 |
| 16740 | 4.34E-05 | 9.51E-04 | transferase activity | 25.64% | 5.62% | Q9D0K2\|Q9CWJ9\|P29758\|Q9CZN7\|P40142\|Q9WTP6\|Q91V92\|Q9CZU6\|Q99KQ4\|P09411 |
| 16829 | 4.81E-05 | 9.51E-04 | lyase activity | 10.26% | 0.51% | Q99KI0\|Q9CZN7\|P17182\|Q91V92 |
| 4372 | 4.92E-05 | 9.51E-04 | glycine hydroxymethyltransferase activity | 5.13% | 0.03% | Q9CWJ9\|Q9CZN7 |
| 166 | 5.53E-05 | 9.62E-04 | nucleotide binding | 28.21% | 7.05% | P32921\|P07901\|P09405\|Q9WTP6\|Q3U2G2\|Q02053\|Q91V92\|P62827\|Q64433\|P14152\|P09411 |
| 42802 | 1.22E-04 | 1.93E-03 | identical protein binding | 12.82% | 1.25% | Q9D0K2\|P07901\|Q9CZN7\|P17182\|Q99KQ4 |
| 48037 | 2.65E-04 | 3.84E-03 | cofactor binding | 10.26% | 0.80% | P29758\|Q9CZN7\|P40142\|P14152 |
| 42803 | 3.36E-04 | 4.05E-03 | protein homodimerization activity | 10.26% | 0.85% | Q9D0K2\|P07901\|P17182\|Q99KQ4 |
| 32555 | 3.48E-04 | 4.05E-03 | purine ribonucleotide binding | 23.08% | 5.88% | P32921\|P07901\|Q9WTP6\|Q3U2G2\|Q02053\|Q91V92\|P62827\|Q64433\|P09411 |
| 32553 | 3.49E-04 | 4.05E-03 | ribonucleotide binding | 23.08% | 5.88% | P32921\|P07901\|Q9WTP6\|Q3U2G2\|Q02053\|Q91V92\|P62827\|Q64433\|P09411 |
| 5524 | 4.17E-04 | 4.53E-03 | ATP binding | 20.51% | 4.75% | P32921\|P07901\|Q9WTP6\|Q3U2G2\|Q02053\|Q91V92\|Q64433\|P09411 |
| 32559 | 4.72E-04 | 4.57E-03 | adenyl ribonucleotide binding | 20.51% | 4.84% | P32921\|P07901\|Q9WTP6\|Q3U2G2\|Q02053\|Q91V92\|Q64433\|P09411 |
| 17076 | 4.73E-04 | 4.57E-03 | purine nucleotide binding | 23.08% | 6.13% | P32921\|P07901\|Q9WTP6\|Q3U2G2\|Q02053\|Q91V92\|P62827\|Q64433\|P09411 |
| 19842 | 5.68E-04 | 5.21E-03 | vitamin binding | 7.69% | 0.41% | P29758\|Q9CZN7\|P40142 |
| 30554 | 6.61E-04 | 5.75E-03 | adenyl nucleotide binding | 20.51% | 5.09% | P32921\|P07901\|Q9WTP6\|Q3U2G2\|Q02053\|Q91V92\|Q64433\|P09411 |
| 1883 | 7.25E-04 | 5.89E-03 | purine nucleoside binding | 20.51% | 5.16% | P32921\|P07901\|Q9WTP6\|Q3U2G2\|Q02053\|Q91V92\|Q64433\|P09411 |
| 1882 | 7.45E-04 | 5.89E-03 | nucleoside binding | 20.51% | 5.18% | P32921\|P07901\|Q9WTP6\|Q3U2G2\|Q02053\|Q91V92\|Q64433\|P09411 |
| 16860 | 1.02E-03 | 7.10E-03 | intramolecular oxidoreductase activity | 5.13% | 0.12% | P27773\|P17751 |
| 3723 | 1.19E-03 | 7.10E-03 | RNA binding | 12.82% | 2.06% | P14211\|P09405\|Q99LX0\|P50580\|P63242 |
| 16836 | 1.33E-03 | 7.10E-03 | hydro-lyase activity | 5.13% | 0.14% | Q99KI0\|P17182 |
| 30337 | 1.35E-03 | 7.10E-03 | DNA polymerase processivity factor activity | 2.56% | 0.00% | P17918 |
| 4643 | 1.35E-03 | 7.10E-03 | phosphoribosylaminoimidazolecarboxamide formyltransferase activity | 2.56% | 0.00% | Q9CWJ9 |
| 4108 | 1.35E-03 | 7.10E-03 | citrate (Si)-synthase activity | 2.56% | 0.00% | Q9CZU6 |
| 47280 | 1.35E-03 | 7.10E-03 | nicotinamide phosphoribosyltransferase activity | 2.56% | 0.00% | Q99KQ4 |
| 3937 | 1.35E-03 | 7.10E-03 | IMP cyclohydrolase activity | 2.56% | 0.00% | Q9CWJ9 |
| 4807 | 1.35E-03 | 7.10E-03 | triose-phosphate isomerase activity | 2.56% | 0.00% | P17751 |
| 4587 | 1.35E-03 | 7.10E-03 | ornithine-oxo-acid transaminase activity | 2.56% | 0.00% | P29758 |
| 46554 | 1.35E-03 | 7.10E-03 | malate dehydrogenase (NADP+) activity | 2.56% | 0.00% | P08249 |
| 30170 | 2.08E-03 | 9.96E-03 | pyridoxal phosphate binding | 5.13% | 0.17% | P29758\|Q9CZN7 |
| 70279 | 2.08E-03 | 9.96E-03 | vitamin B6 binding | 5.13% | 0.17% | P29758\|Q9CZN7 |
| 16835 | 2.25E-03 | 9.96E-03 | carbon-oxygen lyase activity | 5.13% | 0.18% | Q99KI0\|P17182 |
| 46983 | 2.53E-03 | 9.96E-03 | protein dimerization activity | 10.26% | 1.47% | Q9D0K2\|P07901\|P17182\|Q99KQ4 |
| 8260 | 2.69E-03 | 9.96E-03 | 3-oxoacid CoA-transferase activity | 2.56% | 0.01% | Q9D0K2 |
| 4618 | 2.69E-03 | 9.96E-03 | phosphoglycerate kinase activity | 2.56% | 0.01% | P09411 |
| 51538 | 2.69E-03 | 9.96E-03 | 3 iron, 4 sulfur cluster binding | 2.56% | 0.01% | Q99KI0 |
| 8732 | 2.69E-03 | 9.96E-03 | L-allo-threonine aldolase activity | 2.56% | 0.01% | Q9CZN7 |
| 3994 | 2.69E-03 | 9.96E-03 | aconitate hydratase activity | 2.56% | 0.01% | Q99KI0 |
| 30235 | 2.69E-03 | 9.96E-03 | nitric-oxide synthase regulator activity | 2.56% | 0.01% | P07901 |
| 8410 | 2.69E-03 | 9.96E-03 | CoA-transferase activity | 2.56% | 0.01% | Q9D0K2 |
| 4793 | 2.69E-03 | 9.96E-03 | threonine aldolase activity | 2.56% | 0.01% | Q9CZN7 |
| 3878 | 2.69E-03 | 9.96E-03 | ATP citrate synthase activity | 2.56% | 0.01% | Q91V92 |
| 4830 | 2.69E-03 | 9.96E-03 | tryptophan-tRNA ligase activity | 2.56% | 0.01% | P32921 |
| 51082 | 2.98E-03 | 1.08E-02 | unfolded protein binding | 5.13% | 0.21% | P07901\|P14211 |
| 4775 | 4.03E-03 | 1.32E-02 | succinate-CoA ligase (ADP-forming) activity | 2.56% | 0.01% | Q91V92 |
| 4784 | 4.03E-03 | 1.32E-02 | superoxide dismutase activity | 2.56% | 0.01% | P08228 |
| 16721 | 4.03E-03 | 1.32E-02 | oxidoreductase activity, acting on superoxide radicals as acceptor | 2.56% | 0.01% | P08228 |
| 4802 | 4.03E-03 | 1.32E-02 | transketolase activity | 2.56% | 0.01% | P40142 |
| 5094 | 4.03E-03 | 1.32E-02 | Rho GDP-dissociation inhibitor activity | 2.56% | 0.01% | Q99PT1 |
| 16774 | 5.38E-03 | 1.61E-02 | phosphotransferase activity, carboxyl group as acceptor | 2.56% | 0.01% | P09411 |
| 4634 | 5.38E-03 | 1.61E-02 | phosphopyruvate hydratase activity | 2.56% | 0.01% | P17182 |
| 17025 | 5.38E-03 | 1.61E-02 | TATA-binding protein binding | 2.56% | 0.01% | P70670 |
| 4774 | 5.38E-03 | 1.61E-02 | succinate-CoA ligase activity | 2.56% | 0.01% | Q91V92 |
| 16744 | 5.38E-03 | 1.61E-02 | transferase activity, transferring aldehyde or ketonic groups | 2.56% | 0.01% | P40142 |
| 19238 | 6.71E-03 | 1.92E-02 | cyclohydrolase activity | 2.56% | 0.02% | Q9CWJ9 |
| 30976 | 6.71E-03 | 1.92E-02 | thiamin pyrophosphate binding | 2.56% | 0.02% | P40142 |
| 5092 | 6.71E-03 | 1.92E-02 | GDP-dissociation inhibitor activity | 2.56% | 0.02% | Q99PT1 |
| 30971 | 8.05E-03 | 2.22E-02 | receptor tyrosine kinase binding | 2.56% | 0.02% | P61982 |
| 16742 | 8.05E-03 | 2.22E-02 | hydroxymethyl-, formyl- and related transferase activity | 2.56% | 0.02% | Q9CWJ9 |
| 4017 | 9.39E-03 | 2.43E-02 | adenylate kinase activity | 2.56% | 0.02% | Q9WTP6 |
| 3756 | 9.39E-03 | 2.43E-02 | protein disulfide isomerase activity | 2.56% | 0.02% | P27773 |
| 16864 | 9.39E-03 | 2.43E-02 | intramolecular oxidoreductase activity, transposing S-S bonds | 2.56% | 0.02% | P27773 |
| 51920 | 9.39E-03 | 2.43E-02 | peroxiredoxin activity | 2.56% | 0.02% | Q99LX0 |
| 16616 | 9.50E-03 | 2.43E-02 | oxidoreductase activity, acting on the CH-OH group of donors, NAD or NADP as acceptor | 5.13% | 0.38% | P08249\|P14152 |
| 16862 | 1.07E-02 | 2.63E-02 | intramolecular oxidoreductase activity, interconverting keto- and enol-groups | 2.56% | 0.03% | P27773 |
| 16405 | 1.07E-02 | 2.63E-02 | CoA-ligase activity | 2.56% | 0.03% | Q91V92 |
| 8641 | 1.07E-02 | 2.63E-02 | small protein activating enzyme activity | 2.56% | 0.03% | Q02053 |
| 16614 | 1.12E-02 | 2.71E-02 | oxidoreductase activity, acting on CH-OH group of donors | 5.13% | 0.41% | P08249\|P14152 |
| 30234 | 1.15E-02 | 2.73E-02 | enzyme regulator activity | 10.26% | 2.26% | P97822\|P07901\|Q99PT1\|P17918 |
| 16874 | 1.19E-02 | 2.75E-02 | ligase activity | 7.69% | 1.22% | P32921\|Q02053\|Q91V92 |
| 16861 | 1.21E-02 | 2.75E-02 | intramolecular oxidoreductase activity, interconverting aldoses and ketoses | 2.56% | 0.03% | P17751 |
| 16832 | 1.21E-02 | 2.75E-02 | aldehyde-lyase activity | 2.56% | 0.03% | Q9CZN7 |
| 16853 | 1.21E-02 | 2.75E-02 | isomerase activity | 5.13% | 0.43% | P27773\|P17751 |
| 16491 | 1.27E-02 | 2.84E-02 | oxidoreductase activity | 10.26% | 2.34% | P08249\|Q99LX0\|P08228\|P14152 |
| 3676 | 1.44E-02 | 3.16E-02 | nucleic acid binding | 20.51% | 8.41% | P14211\|P09405\|Q99LX0\|Q78PY7\|P70670\|P17918\|P50580\|P63242 |
| 19201 | 1.74E-02 | 3.78E-02 | nucleotide kinase activity | 2.56% | 0.04% | Q9WTP6 |
| 8168 | 1.83E-02 | 3.93E-02 | methyltransferase activity | 5.13% | 0.53% | Q9CWJ9\|Q9CZN7 |
| 16741 | 1.90E-02 | 4.03E-02 | transferase activity, transferring one-carbon groups | 5.13% | 0.54% | Q9CWJ9\|Q9CZN7 |
| 16878 | 2.00E-02 | 4.15E-02 | acid-thiol ligase activity | 2.56% | 0.05% | Q91V92 |
| 43022 | 2.00E-02 | 4.15E-02 | ribosome binding | 2.56% | 0.05% | P63242 |
| 50662 | 2.11E-02 | 4.31E-02 | coenzyme binding | 5.13% | 0.57% | P40142\|P14152 |
| 5506 | 2.13E-02 | 4.31E-02 | iron ion binding | 5.13% | 0.58% | Q99KI0\|P14211 |
| 43531 | 2.27E-02 | 4.53E-02 | ADP binding | 2.56% | 0.06% | P09411 |
| 19212 | 2.53E-02 | 4.94E-02 | phosphatase inhibitor activity | 2.56% | 0.07% | P97822 |
| 51087 | 2.53E-02 | 4.94E-02 | chaperone binding | 2.56% | 0.07% | P08228 |
