# Supplementary material for: Synthesis and Evaluation of a Novel Adenosine-Ribose Probe for Global-Scale Profiling of Nucleoside and Nucleotide-Binding Proteins
Source: PLoS One. 2015 Feb 11;10(2):e0115644. doi: 10.1371/journal.pone.0115644 (PMC4324776; doi:10.1371/journal.pone.0115644)
Supplement: S5 Table — (DOCX) [file pone.0115644.s007.docx]

**Table S5** – Complete list of statistically over-represented GO Biological Process terms in Overall Identified Proteins, according to BiNGO

| GO-ID | P-value | Corr P-value | Description | Cluster frequency | Total Frequency | Genes in test set |
| --- | --- | --- | --- | --- | --- | --- |
| 5488 | 4.26E-88 | 3.07E-85 | binding | 77.08% | 35.57% | P54071\|O35737\|Q9WTX5\|Q8VBV7\|Q91WQ3\|Q8BMS1\|Q8BH59\|P40240\|P34884\|P60122\|Q8K2B3\|O35343\|Q99M87\|O35345\|Q9JMH6\|Q91ZJ5\|P12787\|P46935\|P07724\|Q07813\|P68510\|Q99KV1\|P27048\|Q9DCD0\|Q8BP47\|O08915\|O70133\|P27612\|P53811\|Q9JHS3\|Q3TXS7\|P63101\|Q9ER72\|O08795\|P60764\|Q8R5C5\|P60766\|Q68FL6\|P26883\|O08663\|Q8BK64\|Q6P1B1\|Q3UM45\|Q8BJU0\|Q9DCC4\|Q8BGQ7\|P70296\|Q9CZD3\|P50516\|Q76MZ3\|Q9D6R2\|Q99LX0\|Q8CGK3\|Q3U2G2\|P70168\|P61202\|P60229\|Q99JI6\|P61205\|Q91YM4\|Q60864\|O35593\|Q80TB8\|O70252\|O70251\|P24547\|Q60960\|P97372\|O35381\|Q6ZQ38\|Q9D1D4\|Q9Z1R2\|P61211\|Q8BJW6\|P54227\|O55029\|P47753\|O35841\|Q7TSZ0\|Q8QZY9\|P51660\|P47758\|Q9D5T0\|B7FAU9\|P51150\|Q8BWY3\|Q91VK1\|Q3UJU9\|Q6ZWN5\|P26645\|Q91WK2\|P14206\|Q9Z1N5\|Q8R127\|P07901\|P24668\|P14211\|O55135\|Q9CYG7\|P27661\|Q9QUR6\|P60335\|Q921K2\|P60710\|Q9D1G1\|P61979\|Q61024\|P11440\|P47856\|P23116\|P50580\|Q61171\|Q8QZT1\|Q99LN9\|P28656\|P18242\|P28658\|Q9ERK4\|Q99K85\|P97822\|Q61160\|Q3UHX2\|O35685\|P68040\|Q8K1M6\|Q8R5L1\|P28474\|Q9CXY6\|P28667\|Q9DBZ5\|P61027\|Q99JX4\|Q9Z2N8\|P61021\|P62196\|P80314\|Q64433\|P56399\|P80315\|P80313\|Q6NVF9\|P61222\|O08997\|P12815\|P80318\|P80317\|Q9DBG6\|P80316\|Q05D44\|Q9CQF3\|Q61937\|P53994\|P68033\|P62702\|P62908\|P30681\|P30416\|Q99JY9\|P11438\|Q9Z0N1\|O09106\|Q8CGC7\|P31938\|P61620\|O88712\|P58281\|Q8BKZ9\|P28352\|P25444\|Q8BQM4\|P62821\|P06837\|P68372\|Q99L47\|P62137\|Q62318\|Q61990\|Q8BTZ7\|P48036\|P68369\|P62281\|P59325\|Q9D3D9\|Q9QUI0\|P62827\|P50247\|P62814\|P62141\|P06745\|Q99K48\|P99026\|P62918\|Q91VR5\|P99028\|Q9CQC6\|P99024\|Q99PT1\|P11983\|P62192\|P58252\|P17742\|Q99KQ4\|P14152\|P61161\|Q9Z1F9\|P14148\|O54984\|Q8K310\|Q9R0P9\|P62806\|Q9R1T2\|P10711\|P00375\|P60867\|P57776\|Q7TPV4\|Q61074\|Q61081\|P48962\|Q9ERD7\|Q9CQA3\|Q3U0V1\|P70670\|P62270\|Q8BKC5\|Q9WUK2\|Q8BHC4\|Q9CZ44\|P61079\|Q5SUR0\|P37040\|Q7TMY8\|Q9QYJ0\|Q9QZE7\|Q9QZE5\|Q9CZ30\|Q9JM76\|Q3THE2\|P10518\|P68254\|P62962\|Q62348\|P10126\|P62259\|Q9DCT8\|P46664\|P17918\|P19096\|P62869\|P62960\|P62631\|Q99P72\|Q99PV0\|P35700\|Q91V92\|Q9WUM4\|Q9CR16\|Q03265\|P46638\|Q8K224\|Q8VEM8\|Q9R0E1\|Q6PK77\|Q91XV3\|Q3TMX5\|Q99P88\|P20029\|Q6PB66\|Q8K1R3\|P10639\|P61759\|P61089\|Q61753\|Q9WUL7\|Q62465\|P62843\|Q9D1M4\|Q99KI0\|Q9CQI6\|Q9QZB7\|Q8R1B4\|Q9D1J3\|Q8VDD5\|P62897\|Q6P5F9\|Q62261\|P43274\|P43276\|Q9R1K9\|P62320\|Q9D8E6\|P56480\|P67984\|P17182\|Q99KJ8\|P62627\|P62317\|P29758\|Q78PY7\|Q8BMF4\|Q9D0R2\|P61982\|Q8R574\|Q6NZJ6\|Q01853\|P08113\|Q9CZY3\|Q9QZD9\|P16460\|Q9D024\|P62204\|Q8C1A5\|Q3V0I7\|Q3THK7\|Q61425\|O70591\|P05132\|Q6P5E4\|P62334\|P62075\|P32921\|Q9CZN7\|O35114\|Q60931\|Q60932\|P97449\|P18760\|Q921M3\|Q9QYB1\|Q64737\|Q9D0F9\|Q02053\|Q9CX34\|Q6NZD2\|O35129\|P20108\|Q9EPL8\|O88543\|P08228\|Q8BFZ3\|P97351\|P09411\|Q8BFR5\|P09405\|Q9D6J6\|P46460\|P63242\|Q9DB77\|Q9CQX2\|P45591\|P08249\|Q8K4Z5\|P00405\|Q8BG05\|Q91Z38\|Q920E5\|Q8BMJ2\|Q9CPU0\|Q9DCN2\|Q7TT37\|O88569\|Q99MD9\|Q61553\|Q9WV55\|Q9D0K2\|Q9CZR8\|P42932\|P07356\|P63158\|P63017\|P35564\|P35979\|P16045\|Q9JKR6\|Q61584\|Q8VDM4\|Q9D8N0\|Q3THS6\|Q62084\|P32067\|Q9CW46\|Q6ZWX6\|Q8VIJ6\|Q9QXB9\|Q3U9G9\|Q3UID0\|Q9D0I9\|P26040\|Q9WU28\|P63038\|P52480\|Q9Z1D1\|O35900\|Q9CZ13\|Q60716\|O70503\|Q9QZM0\|Q8R081\|P15532\|Q8VHM5\|Q6NZB0\|Q68FD5\|Q99LC5\|P05202\|Q9WTP6\|Q80UG5\|P35278\|A2AG68\|Q9DB20\|Q93092\|P40142\|Q9JM14\|Q9D819\|Q91VD9\|Q61699\|P35282\|Q8VDN2 |
| 166 | 1.26E-53 | 4.53E-51 | nucleotide binding | 28.65% | 7.06% | P54071\|P32921\|O35737\|Q61024\|Q60931\|Q60932\|Q91WQ3\|Q8BMS1\|P11440\|Q9WUK2\|Q8BHC4\|P61079\|Q64737\|Q5SUR0\|P60122\|Q8K2B3\|P37040\|Q9QYJ0\|Q02053\|Q99M87\|Q9JMH6\|Q91ZJ5\|Q8BFZ3\|Q9CZ30\|P09411\|Q8BFR5\|P09405\|Q9D6J6\|P10126\|P46664\|Q8K1M6\|P46460\|Q9CXY6\|Q9DCD0\|Q8BP47\|P62631\|P61027\|Q9Z2N8\|Q91V92\|P61021\|P62196\|P80314\|O70133\|Q64433\|P80315\|P80313\|Q03265\|P80318\|P61222\|Q6NVF9\|P80317\|P80316\|Q9ER72\|P46638\|Q05D44\|Q8BG05\|Q8K224\|P60764\|Q6PK77\|P68033\|P53994\|Q8BMJ2\|Q8R5C5\|P60766\|Q68FL6\|Q9DCN2\|P20029\|P30416\|Q99JY9\|Q9Z0N1\|Q8CGC7\|Q7TT37\|P61089\|P31938\|Q8BGQ7\|O88712\|O88569\|P58281\|Q9WUL7\|Q61753\|Q9CZD3\|P70296\|P50516\|Q9D6R2\|Q8CGK3\|Q3U2G2\|P62821\|P42932\|P61205\|Q99JI6\|P63017\|P68372\|Q99L47\|Q91YM4\|Q8BTZ7\|P59325\|Q8VDD5\|Q9JKR6\|P62827\|Q9QUI0\|Q9R1K9\|Q3THS6\|P50247\|P32067\|P56480\|P62814\|Q9CW46\|P61211\|Q99K48\|Q8VIJ6\|Q9QXB9\|Q91VR5\|Q8QZY9\|Q7TSZ0\|P99024\|P47758\|Q9D5T0\|P11983\|Q9D0I9\|P62192\|Q9D0R2\|P58252\|P63038\|P52480\|P51150\|Q01853\|Q9Z1D1\|P08113\|P14152\|Q8R081\|P16460\|Q9Z1N5\|P61161\|P15532\|P07901\|Q9Z1F9\|Q99LC5\|O54984\|Q8K310\|P00375\|Q3THK7\|Q9WTP6\|Q61425\|Q80UG5\|P35278\|A2AG68\|P05132\|Q921K2\|Q9ERD7\|P62334\|Q9JM14\|Q9D1G1\|Q61699\|P35282\|Q8VDN2 |
| 3824 | 2.10E-41 | 5.06E-39 | catalytic activity | 41.04% | 16.80% | P54071\|Q9WTX5\|Q91YI0\|Q8BMS1\|Q91WQ3\|Q9Z2U1\|Q8BHC4\|P61079\|Q5SUR0\|P34884\|Q8K2B3\|P60122\|P37040\|Q7TMY8\|Q9JMH6\|P19157\|Q91ZJ5\|P12787\|P46935\|Q9EP69\|Q9CZ30\|Q3THE2\|P10518\|P68254\|Q8BZA9\|Q9CWJ9\|P10126\|P62259\|P46664\|P19096\|Q9CZU6\|Q921H8\|Q9DCD0\|Q8BP47\|P62631\|P35700\|Q91V92\|O70133\|Q9CR16\|Q03265\|Q9ER72\|O08795\|Q8K224\|Q9R0E1\|Q6PK77\|P60766\|Q68FL6\|O08663\|P26883\|Q8K1R3\|Q6P1B1\|P10639\|P61089\|Q8BGQ7\|Q9DCC4\|P50518\|Q61469\|Q9CZD3\|Q61753\|P50516\|Q9D6R2\|Q99LX0\|Q62465\|Q8CGK3\|Q99KI0\|Q99JI6\|P58321\|Q91YM4\|Q8VDD5\|O35593\|Q80TB8\|O70252\|P24547\|Q9R1K9\|P56480\|P17182\|Q99KJ8\|P62627\|O09061\|P27773\|P29758\|P51660\|Q78PY7\|Q9D5T0\|Q8BMF4\|Q9D0R2\|Q8R574\|O08553\|Q01853\|Q9Z1N5\|Q8R127\|P62874\|P16460\|Q8C1A5\|Q3V0I7\|Q9DCG9\|Q3THK7\|Q61425\|Q9QUR6\|P05132\|Q921K2\|Q6P5E4\|P62334\|Q64674\|P16858\|P70349\|P32921\|Q9CZN7\|Q61024\|P11440\|P97449\|P47856\|Q64737\|Q61171\|Q8QZT1\|Q9D0F9\|Q99LN9\|Q02053\|P18242\|P20108\|Q922R8\|P08228\|P18155\|Q99K85\|P09411\|Q8BFR5\|Q9D6J6\|Q8K1M6\|P28474\|P46460\|Q9CXY6\|P61957\|Q9DB77\|Q6P1J1\|P61021\|Q9D051\|P62196\|P56399\|O08709\|P61222\|Q9DBG6\|P08249\|Q05D44\|P00405\|Q9R1P0\|Q9R1P1\|Q920E5\|Q91VA7\|P09103\|Q8BMJ2\|Q9CPU0\|Q9DCN2\|Q9CQ65\|P30416\|Q9Z0N1\|O09106\|Q8CGC7\|Q7TT37\|P31938\|Q9DBJ1\|O88712\|P58281\|Q9WV54\|Q8BKZ9\|P28352\|Q91V12\|Q9D0K2\|P61290\|P63017\|P68372\|P62137\|Q62318\|Q8BTZ7\|Q9D3D9\|Q9QUI0\|P62827\|Q3THS6\|Q91V64\|P50247\|P62814\|P62141\|P17751\|P06745\|P99026\|Q91VR5\|P99028\|P99024\|P45376\|Q9D0I9\|P62192\|P58252\|P52480\|Q9CZ13\|Q99KQ4\|Q60716\|P13439\|P14152\|O70503\|P15532\|Q9CQB4\|O08807\|Q60710\|Q9Z1F9\|O54984\|Q9R0P9\|Q9R1T2\|P00375\|P05202\|Q9WTP6\|P35278\|Q7TPV4\|A2AG68\|Q61074\|Q93092\|Q9ERD7\|P40142\|Q9JM14\|Q9D819\|Q91VD9\|Q9CQA3\|Q8CIG8\|Q8VDN2 |
| 5515 | 1.48E-40 | 2.66E-38 | protein binding | 43.25% | 18.61% | Q9WTX5\|Q8VBV7\|Q8BKC5\|Q9CZ44\|P40240\|P34884\|P37040\|Q9QYJ0\|O35343\|Q99M87\|O35345\|Q9QZE7\|Q9QZE5\|P46935\|Q9JM76\|P10518\|P68254\|P62962\|Q62348\|P10126\|Q07813\|P68510\|P62259\|Q99KV1\|P17918\|P62869\|P27048\|P62960\|P62631\|O08915\|Q99P72\|P35700\|P27612\|Q9WUM4\|Q9JHS3\|P63101\|O08795\|P60764\|Q91XV3\|Q8R5C5\|Q3TMX5\|P60766\|P26883\|O08663\|Q99P88\|P20029\|Q8BK64\|Q6PB66\|Q3UM45\|Q8BJU0\|P10639\|P61759\|P61089\|Q9DCC4\|Q9CZD3\|P70296\|Q76MZ3\|P70168\|P62843\|Q9D1M4\|P61202\|Q9CQI6\|Q99JI6\|Q9QZB7\|Q60864\|Q9D1J3\|Q8VDD5\|Q6P5F9\|Q62261\|P43274\|P43276\|Q9R1K9\|Q9D8E6\|P97372\|P56480\|O35381\|P17182\|Q6ZQ38\|Q9D1D4\|Q99KJ8\|P62627\|Q9Z1R2\|P54227\|O55029\|P47753\|Q7TSZ0\|P47758\|Q78PY7\|Q8BMF4\|B7FAU9\|P61982\|P51150\|Q91VK1\|Q6NZJ6\|Q01853\|P08113\|Q9CZY3\|P26645\|P14206\|Q91WK2\|P07901\|P24668\|P14211\|P27661\|Q3V0I7\|Q3THK7\|O70591\|P60335\|P05132\|Q921K2\|P60710\|Q6P5E4\|P61979\|Q9CZN7\|Q61024\|O35114\|P11440\|P23116\|P18760\|Q9QYB1\|Q61171\|Q8QZT1\|Q6NZD2\|P28656\|P28658\|O35129\|P20108\|Q9EPL8\|O88543\|P08228\|Q9ERK4\|Q8BFZ3\|P97351\|P97822\|Q61160\|P09405\|Q3UHX2\|O35685\|P68040\|Q8R5L1\|P46460\|P28474\|P28667\|Q9DBZ5\|P61027\|Q9DB77\|Q9Z2N8\|P62196\|P80314\|P56399\|P80315\|P80313\|P45591\|P12815\|P80318\|P08249\|P80317\|P80316\|Q61937\|P68033\|P30681\|P30416\|Q99JY9\|P11438\|O09106\|P31938\|O88712\|O88569\|Q8BKZ9\|P28352\|Q9WV55\|Q61553\|Q99MD9\|P25444\|Q9D0K2\|P07356\|P42932\|P06837\|P63158\|P63017\|P35564\|Q99L47\|Q61990\|Q62318\|P62137\|P68369\|P48036\|P35979\|P16045\|Q61584\|Q9D3D9\|P62827\|Q9QUI0\|Q8VDM4\|P50247\|Q62084\|P62141\|P06745\|Q99K48\|Q6ZWX6\|Q8VIJ6\|P99028\|P99024\|Q99PT1\|P11983\|P26040\|Q9WU28\|P63038\|P52480\|O35900\|Q9CZ13\|P17742\|Q99KQ4\|Q60716\|O70503\|P61161\|P15532\|Q6NZB0\|Q9Z1F9\|Q68FD5\|P14148\|Q9R0P9\|P62806\|P10711\|P05202\|Q7TPV4\|A2AG68\|Q9DB20\|Q61081\|P48962\|P70670\|Q61699\|P35282\|Q8VDN2 |
| 17076 | 8.63E-40 | 1.24E-37 | purine nucleotide binding | 23.29% | 6.14% | P32921\|Q61024\|Q91WQ3\|P11440\|Q8BHC4\|P61079\|Q64737\|Q5SUR0\|P60122\|Q8K2B3\|P37040\|Q9QYJ0\|Q02053\|Q99M87\|Q9JMH6\|Q8BFZ3\|Q9CZ30\|P09411\|Q8BFR5\|P10126\|P46664\|Q8K1M6\|P46460\|Q9CXY6\|Q8BP47\|P62631\|P61027\|Q9Z2N8\|P61021\|Q91V92\|P62196\|P80314\|O70133\|Q64433\|P80315\|P80313\|P61222\|P80318\|Q03265\|P80317\|P80316\|Q9ER72\|Q05D44\|P46638\|P60764\|Q8K224\|P53994\|P68033\|Q6PK77\|Q8BMJ2\|Q8R5C5\|P60766\|Q68FL6\|Q9DCN2\|P20029\|P30416\|Q99JY9\|Q9Z0N1\|Q8CGC7\|Q7TT37\|P31938\|P61089\|Q8BGQ7\|P58281\|Q9CZD3\|P70296\|Q9WUL7\|P50516\|Q8CGK3\|Q3U2G2\|P62821\|P42932\|Q99JI6\|P61205\|P63017\|P68372\|Q99L47\|Q91YM4\|Q8BTZ7\|P59325\|Q8VDD5\|Q9JKR6\|P62827\|Q9QUI0\|Q9R1K9\|Q3THS6\|P50247\|P56480\|P62814\|P61211\|Q9QXB9\|Q91VR5\|Q7TSZ0\|P99024\|P47758\|Q9D5T0\|P11983\|Q9D0I9\|P62192\|Q9D0R2\|P58252\|P63038\|P52480\|P51150\|Q01853\|P08113\|Q9Z1N5\|P61161\|P16460\|P15532\|P07901\|Q9Z1F9\|Q99LC5\|O54984\|Q3THK7\|Q9WTP6\|Q80UG5\|P35278\|A2AG68\|P05132\|Q9ERD7\|P62334\|Q9D1G1\|Q61699\|P35282\|Q8VDN2 |
| 32553 | 3.76E-38 | 4.52E-36 | ribonucleotide binding | 22.37% | 5.89% | P32921\|Q61024\|Q91WQ3\|P11440\|Q8BHC4\|P61079\|Q64737\|Q5SUR0\|P60122\|Q9QYJ0\|Q02053\|Q99M87\|Q91ZJ5\|Q8BFZ3\|Q9CZ30\|P09411\|Q8BFR5\|P10126\|P46664\|Q8K1M6\|P46460\|Q9CXY6\|Q8BP47\|P62631\|P61027\|Q9Z2N8\|P61021\|Q91V92\|P62196\|P80314\|O70133\|Q64433\|P80315\|P80313\|P61222\|P80318\|Q03265\|P80317\|P80316\|Q9ER72\|Q05D44\|P46638\|P60764\|Q8K224\|P53994\|P68033\|Q6PK77\|Q8BMJ2\|Q8R5C5\|P60766\|Q68FL6\|Q9DCN2\|P20029\|P30416\|Q99JY9\|Q9Z0N1\|Q8CGC7\|Q7TT37\|P31938\|P61089\|Q8BGQ7\|P58281\|Q9CZD3\|P70296\|Q9WUL7\|P50516\|Q8CGK3\|Q3U2G2\|P62821\|P42932\|Q99JI6\|P61205\|P63017\|P68372\|Q91YM4\|Q8BTZ7\|P59325\|Q8VDD5\|Q9JKR6\|P62827\|Q9QUI0\|Q9R1K9\|Q3THS6\|P56480\|P62814\|P61211\|Q9QXB9\|Q91VR5\|Q7TSZ0\|P99024\|P47758\|Q9D5T0\|P11983\|Q9D0I9\|P62192\|Q9D0R2\|P58252\|P63038\|P52480\|P51150\|Q01853\|P08113\|Q9Z1N5\|P61161\|P16460\|P15532\|P07901\|Q9Z1F9\|O54984\|Q3THK7\|Q9WTP6\|Q80UG5\|P35278\|A2AG68\|P05132\|Q9ERD7\|P62334\|Q9D1G1\|Q61699\|P35282\|Q8VDN2 |
| 32555 | 1.73E-37 | 1.79E-35 | purine ribonucleotide binding | 22.18% | 5.88% | P32921\|Q61024\|Q91WQ3\|P11440\|Q8BHC4\|P61079\|Q64737\|Q5SUR0\|P60122\|Q9QYJ0\|Q02053\|Q99M87\|Q8BFZ3\|Q9CZ30\|P09411\|Q8BFR5\|P10126\|P46664\|Q8K1M6\|P46460\|Q9CXY6\|Q8BP47\|P62631\|P61027\|Q9Z2N8\|P61021\|Q91V92\|P62196\|P80314\|O70133\|Q64433\|P80315\|P80313\|P61222\|P80318\|Q03265\|P80317\|P80316\|Q9ER72\|Q05D44\|P46638\|P60764\|Q8K224\|P53994\|P68033\|Q6PK77\|Q8BMJ2\|Q8R5C5\|P60766\|Q68FL6\|Q9DCN2\|P20029\|P30416\|Q99JY9\|Q9Z0N1\|Q8CGC7\|Q7TT37\|P31938\|P61089\|Q8BGQ7\|P58281\|Q9CZD3\|P70296\|Q9WUL7\|P50516\|Q8CGK3\|Q3U2G2\|P62821\|P42932\|Q99JI6\|P61205\|P63017\|P68372\|Q91YM4\|Q8BTZ7\|P59325\|Q8VDD5\|Q9JKR6\|P62827\|Q9QUI0\|Q9R1K9\|Q3THS6\|P56480\|P62814\|P61211\|Q9QXB9\|Q91VR5\|Q7TSZ0\|P99024\|P47758\|Q9D5T0\|P11983\|Q9D0I9\|P62192\|Q9D0R2\|P58252\|P63038\|P52480\|P51150\|Q01853\|P08113\|Q9Z1N5\|P61161\|P16460\|P15532\|P07901\|Q9Z1F9\|O54984\|Q3THK7\|Q9WTP6\|Q80UG5\|P35278\|A2AG68\|P05132\|Q9ERD7\|P62334\|Q9D1G1\|Q61699\|P35282\|Q8VDN2 |
| 3735 | 2.84E-34 | 2.56E-32 | structural constituent of ribosome | 6.47% | 0.36% | P62270\|Q9CQR2\|P35980\|Q9DB15\|Q6ZWV3\|P62754\|P14869\|P63325\|Q6ZWN5\|P14206\|P62281\|P47911\|P14148\|P62264\|P62717\|P62830\|P47955\|P62245\|P63276\|P62702\|P62908\|Q9D8E6\|P62852\|P60867\|P67984\|P62082\|P97351\|Q9CZX8\|P62918\|P99027\|Q8BP67\|Q3TBW2\|P51410\|P25444\|P62843 |
| 5198 | 3.32E-27 | 2.66E-25 | structural molecule activity | 8.87% | 1.21% | P62270\|Q9CQR2\|P35980\|Q9DB15\|Q6ZWV3\|P68372\|P62754\|P14869\|P63325\|Q6ZWN5\|P14206\|P62281\|P50446\|P47911\|Q68FD5\|P14148\|Q62261\|P62717\|P62264\|P62830\|P47955\|P62245\|P63276\|Q9D8E6\|P62702\|P62908\|P62852\|Q99P88\|P60867\|Q9QZE5\|P67984\|P62082\|Q3UV17\|P14733\|P97351\|Q9CZX8\|P62918\|P99027\|O55029\|Q9ERD7\|Q9WV55\|Q6IFZ6\|Q8BP67\|P99024\|Q3TBW2\|P51410\|P25444\|P62843 |
| 8135 | 4.26E-26 | 3.07E-24 | translation factor activity, nucleic acid binding | 5.18% | 0.32% | Q9CZR8\|P58252\|P60229\|Q9DBZ5\|P62631\|Q8BWY3\|Q6NZJ6\|Q9Z1D1\|Q99JX4\|P23116\|Q9WUK2\|Q8R1B4\|Q9QZD9\|Q91WK2\|P59325\|O55135\|Q05D44\|O70251\|P10711\|Q9D8N0\|P57776\|Q9Z0N1\|Q8BJW6\|Q8BFR5\|Q6ZWX6\|P10126\|Q9D1M4\|P63242 |
| 3723 | 1.35E-25 | 8.86E-24 | RNA binding | 10.91% | 2.07% | P62270\|P61979\|O35737\|P63158\|Q91WQ3\|P50580\|Q9WUK2\|Q61990\|P62281\|Q6P5F9\|Q61584\|P62320\|P32067\|P67984\|Q9CW46\|Q99K48\|Q6ZWX6\|P62918\|Q8VIJ6\|Q91VR5\|Q62348\|P09405\|Q8QZY9\|Q9D0I9\|P27048\|Q9CXY6\|P62960\|P63242\|Q9DBZ5\|Q6NZJ6\|Q9Z1D1\|Q99PV0\|Q6ZWN5\|O35900\|O70133\|Q6NVF9\|Q8R081\|Q9Z1N5\|P14211\|Q8K4Z5\|Q9CQF3\|O08795\|P14148\|Q61937\|Q8BG05\|Q8K310\|P62702\|P62908\|Q68FL6\|P60867\|Q6PB66\|Q8K1R3\|Q8CGC7\|Q8BGQ7\|O88569\|P60335\|Q99LX0\|Q3U0V1\|P62843 |
| 1883 | 8.99E-25 | 5.40E-23 | purine nucleoside binding | 17.19% | 5.17% | P42932\|P32921\|Q61024\|P63017\|Q91WQ3\|P11440\|Q99L47\|Q8BHC4\|P61079\|Q91YM4\|Q64737\|Q5SUR0\|Q8VDD5\|P37040\|Q8K2B3\|P60122\|Q9QYJ0\|Q9JKR6\|Q02053\|Q99M87\|Q9JMH6\|Q9R1K9\|Q3THS6\|P50247\|P56480\|P62814\|Q8BFZ3\|Q9CZ30\|P09411\|Q91VR5\|Q7TSZ0\|Q9D5T0\|P11983\|Q9D0I9\|P46460\|P62192\|Q9D0R2\|Q9CXY6\|P63038\|P52480\|Q8BP47\|Q01853\|P08113\|Q9Z2N8\|Q91V92\|O70133\|P80314\|P62196\|P80315\|Q64433\|P80313\|Q03265\|P80318\|P61222\|P16460\|Q9Z1N5\|P80317\|P61161\|P15532\|P07901\|P80316\|Q9ER72\|Q9Z1F9\|Q99LC5\|O54984\|Q8K224\|Q6PK77\|P68033\|Q8BMJ2\|Q8R5C5\|Q68FL6\|Q9DCN2\|P20029\|Q3THK7\|Q9WTP6\|P30416\|Q99JY9\|Q8CGC7\|Q7TT37\|P61089\|P31938\|Q8BGQ7\|A2AG68\|Q9WUL7\|P70296\|Q9CZD3\|P05132\|P50516\|P62334\|Q3U2G2\|Q8CGK3\|Q61699\|Q8VDN2 |
| 1882 | 1.20E-24 | 6.64E-23 | nucleoside binding | 17.19% | 5.19% | P42932\|P32921\|Q61024\|P63017\|Q91WQ3\|P11440\|Q99L47\|Q8BHC4\|P61079\|Q91YM4\|Q64737\|Q5SUR0\|Q8VDD5\|P37040\|Q8K2B3\|P60122\|Q9QYJ0\|Q9JKR6\|Q02053\|Q99M87\|Q9JMH6\|Q9R1K9\|Q3THS6\|P50247\|P56480\|P62814\|Q8BFZ3\|Q9CZ30\|P09411\|Q91VR5\|Q7TSZ0\|Q9D5T0\|P11983\|Q9D0I9\|P46460\|P62192\|Q9D0R2\|Q9CXY6\|P63038\|P52480\|Q8BP47\|Q01853\|P08113\|Q9Z2N8\|Q91V92\|O70133\|P80314\|P62196\|P80315\|Q64433\|P80313\|Q03265\|P80318\|P61222\|P16460\|Q9Z1N5\|P80317\|P61161\|P15532\|P07901\|P80316\|Q9ER72\|Q9Z1F9\|Q99LC5\|O54984\|Q8K224\|Q6PK77\|P68033\|Q8BMJ2\|Q8R5C5\|Q68FL6\|Q9DCN2\|P20029\|Q3THK7\|Q9WTP6\|P30416\|Q99JY9\|Q8CGC7\|Q7TT37\|P61089\|P31938\|Q8BGQ7\|A2AG68\|Q9WUL7\|P70296\|Q9CZD3\|P05132\|P50516\|P62334\|Q3U2G2\|Q8CGK3\|Q61699\|Q8VDN2 |
| 30554 | 1.32E-24 | 6.80E-23 | adenyl nucleotide binding | 17.01% | 5.09% | P42932\|P32921\|Q61024\|P63017\|Q91WQ3\|P11440\|Q99L47\|Q8BHC4\|P61079\|Q91YM4\|Q64737\|Q5SUR0\|Q8VDD5\|P37040\|Q8K2B3\|P60122\|Q9QYJ0\|Q9JKR6\|Q02053\|Q99M87\|Q9JMH6\|Q9R1K9\|Q3THS6\|P50247\|P56480\|P62814\|Q8BFZ3\|Q9CZ30\|P09411\|Q91VR5\|Q7TSZ0\|Q9D5T0\|P11983\|Q9D0I9\|P46460\|P62192\|Q9D0R2\|Q9CXY6\|P63038\|P52480\|Q8BP47\|Q01853\|P08113\|Q9Z2N8\|Q91V92\|O70133\|P80314\|P62196\|P80315\|Q64433\|P80313\|Q03265\|P80318\|P61222\|P16460\|Q9Z1N5\|P80317\|P61161\|P15532\|P07901\|P80316\|Q9ER72\|Q9Z1F9\|Q99LC5\|O54984\|Q8K224\|Q6PK77\|P68033\|Q8BMJ2\|Q8R5C5\|Q68FL6\|Q9DCN2\|P20029\|Q3THK7\|Q9WTP6\|P30416\|Q99JY9\|Q8CGC7\|Q7TT37\|P61089\|P31938\|Q8BGQ7\|A2AG68\|P70296\|Q9CZD3\|P05132\|P50516\|P62334\|Q3U2G2\|Q8CGK3\|Q61699\|Q8VDN2 |
| 51082 | 4.58E-23 | 2.20E-21 | unfolded protein binding | 4.07% | 0.21% | Q9WU28\|P42932\|P63017\|P35564\|P08113\|P61759\|Q99L47\|O70591\|P80314\|P80315\|P80313\|P80318\|P80317\|P07901\|P80316\|Q6P5E4\|P14211\|Q9QYJ0\|Q7TSZ0\|Q99M87\|Q99KV1\|P11983 |
| 32559 | 1.64E-22 | 7.38E-21 | adenyl ribonucleotide binding | 15.90% | 4.84% | P42932\|P32921\|Q61024\|P63017\|Q91WQ3\|P11440\|Q8BHC4\|Q91YM4\|P61079\|Q64737\|Q5SUR0\|Q8VDD5\|P60122\|Q9QYJ0\|Q9JKR6\|Q02053\|Q99M87\|Q9R1K9\|Q3THS6\|P56480\|P62814\|Q8BFZ3\|Q9CZ30\|P09411\|Q91VR5\|Q7TSZ0\|Q9D5T0\|P11983\|Q9D0I9\|P46460\|P62192\|Q9D0R2\|Q9CXY6\|P63038\|P52480\|Q8BP47\|Q01853\|P08113\|Q9Z2N8\|Q91V92\|P80314\|O70133\|P62196\|P80315\|Q64433\|P80313\|Q03265\|P80318\|P61222\|P16460\|Q9Z1N5\|P80317\|P61161\|P15532\|P07901\|P80316\|Q9ER72\|Q9Z1F9\|O54984\|Q8K224\|Q6PK77\|P68033\|Q8BMJ2\|Q8R5C5\|Q68FL6\|Q9DCN2\|P20029\|Q3THK7\|Q9WTP6\|P30416\|Q99JY9\|Q8CGC7\|Q7TT37\|P61089\|P31938\|Q8BGQ7\|A2AG68\|Q9CZD3\|P70296\|P05132\|P50516\|P62334\|Q3U2G2\|Q8CGK3\|Q61699\|Q8VDN2 |
| 5524 | 7.43E-22 | 3.15E-20 | ATP binding | 15.53% | 4.75% | P42932\|P32921\|Q61024\|P63017\|Q91WQ3\|P11440\|Q8BHC4\|Q91YM4\|P61079\|Q64737\|Q5SUR0\|Q8VDD5\|P60122\|Q9QYJ0\|Q9JKR6\|Q02053\|Q99M87\|Q9R1K9\|Q3THS6\|P56480\|P62814\|Q8BFZ3\|Q9CZ30\|P09411\|Q91VR5\|Q7TSZ0\|Q9D5T0\|P11983\|Q9D0I9\|P46460\|P62192\|Q9D0R2\|Q9CXY6\|P63038\|P52480\|Q8BP47\|Q01853\|P08113\|Q9Z2N8\|Q91V92\|P80314\|O70133\|P62196\|P80315\|Q64433\|P80313\|Q03265\|P80318\|P61222\|P16460\|Q9Z1N5\|P80317\|P61161\|P15532\|P07901\|P80316\|Q9ER72\|Q9Z1F9\|O54984\|Q8K224\|P68033\|Q8BMJ2\|Q8R5C5\|Q68FL6\|P20029\|Q3THK7\|Q9WTP6\|P30416\|Q99JY9\|Q8CGC7\|Q7TT37\|P61089\|P31938\|Q8BGQ7\|A2AG68\|Q9CZD3\|P70296\|P05132\|P50516\|P62334\|Q3U2G2\|Q8CGK3\|Q61699\|Q8VDN2 |
| 5525 | 5.62E-18 | 2.25E-16 | GTP binding | 6.65% | 1.10% | P62821\|P58252\|P62631\|P51150\|P61027\|P61205\|Q99JI6\|P68372\|P61021\|Q8BTZ7\|P59325\|P46638\|Q05D44\|P60764\|P53994\|P62827\|Q9QUI0\|P60766\|P30416\|Q80UG5\|P35278\|Q9Z0N1\|P61211\|Q9CZ30\|Q8BFR5\|P58281\|Q9WUL7\|Q9QXB9\|Q9ERD7\|P10126\|Q9D1G1\|P99024\|P47758\|P46664\|Q8K1M6\|P35282 |
| 32561 | 2.78E-17 | 1.00E-15 | guanyl ribonucleotide binding | 6.65% | 1.15% | P62821\|P58252\|P62631\|P51150\|P61027\|P61205\|Q99JI6\|P68372\|P61021\|Q8BTZ7\|P59325\|P46638\|Q05D44\|P60764\|P53994\|P62827\|Q9QUI0\|P60766\|P30416\|Q80UG5\|P35278\|Q9Z0N1\|P61211\|Q9CZ30\|Q8BFR5\|P58281\|Q9WUL7\|Q9QXB9\|Q9ERD7\|P10126\|Q9D1G1\|P99024\|P47758\|P46664\|Q8K1M6\|P35282 |
| 19001 | 2.78E-17 | 1.00E-15 | guanyl nucleotide binding | 6.65% | 1.15% | P62821\|P58252\|P62631\|P51150\|P61027\|P61205\|Q99JI6\|P68372\|P61021\|Q8BTZ7\|P59325\|P46638\|Q05D44\|P60764\|P53994\|P62827\|Q9QUI0\|P60766\|P30416\|Q80UG5\|P35278\|Q9Z0N1\|P61211\|Q9CZ30\|Q8BFR5\|P58281\|Q9WUL7\|Q9QXB9\|Q9ERD7\|P10126\|Q9D1G1\|P99024\|P47758\|P46664\|Q8K1M6\|P35282 |
| 16491 | 1.66E-16 | 5.71E-15 | oxidoreductase activity | 9.24% | 2.34% | P54071\|Q8BMS1\|Q61171\|Q8K2B3\|P37040\|Q99LN9\|Q80TB8\|O70252\|Q9JMH6\|P24547\|P20108\|P12787\|P08228\|P18155\|P68254\|P99028\|Q9D6J6\|P62259\|P51660\|P45376\|P19096\|P28474\|Q9DCD0\|P35700\|Q9D051\|P14152\|Q60716\|O70503\|O08709\|Q8R127\|P08249\|Q9CQB4\|O08807\|P00405\|Q9R0E1\|Q91VA7\|P09103\|P00375\|Q9DCN2\|Q61425\|P10639\|Q9DCC4\|O88712\|Q61753\|Q9D6R2\|Q91VD9\|Q99LX0\|Q9CQA3\|Q62465\|P16858 |
| 3676 | 1.08E-15 | 3.53E-14 | nucleic acid binding | 19.22% | 8.42% | P62270\|O35737\|P61979\|Q91WQ3\|P23116\|P50580\|Q9WUK2\|Q921M3\|Q7TMY8\|Q9QZE7\|Q8BFR5\|Q62348\|P09405\|P10126\|P17918\|P27048\|Q9CXY6\|P62960\|P63242\|Q9DBZ5\|Q8BP47\|P62631\|Q99JX4\|Q99PV0\|O70133\|Q6NVF9\|Q8K4Z5\|Q05D44\|Q9CQF3\|O08795\|Q61937\|Q8BG05\|Q91XV3\|P62702\|P62908\|Q68FL6\|P30681\|Q6PB66\|Q8K1R3\|Q9Z0N1\|O09106\|Q8CGC7\|Q8BGQ7\|O88712\|O88569\|P28352\|Q99LX0\|Q8CGK3\|P62843\|Q9D1M4\|Q9CZR8\|P60229\|P63158\|Q61990\|Q8R1B4\|P62281\|P59325\|Q6P5F9\|P43274\|Q61584\|P43276\|O70251\|Q9R1K9\|P62320\|Q9D8N0\|P32067\|P67984\|Q9CW46\|Q99K48\|Q8BJW6\|P62918\|Q6ZWX6\|Q8VIJ6\|Q91VR5\|P62317\|Q3U9G9\|Q3UID0\|Q8QZY9\|Q78PY7\|Q9D0I9\|P58252\|Q8BWY3\|Q6NZJ6\|Q9Z1D1\|Q6ZWN5\|O35900\|Q9QZD9\|Q91WK2\|Q8R081\|Q9Z1N5\|Q8VHM5\|O55135\|P14211\|P14148\|P27661\|Q8K310\|P10711\|P60867\|P57776\|Q7TPV4\|P60335\|Q921K2\|Q3U0V1\|P70670 |
| 17111 | 2.02E-15 | 6.33E-14 | nucleoside-triphosphatase activity | 8.13% | 1.96% | P58252\|P62631\|Q99JI6\|Q01853\|P63017\|P68372\|P61021\|P62196\|O70133\|Q03265\|P61222\|P62874\|Q9Z1N5\|Q8VDD5\|P60122\|Q05D44\|Q9D3D9\|P62827\|Q9QUI0\|Q9R1K9\|P60766\|P56480\|P62814\|P35278\|Q9Z0N1\|P62627\|Q99KJ8\|A2AG68\|P50518\|Q8BFR5\|Q3THE2\|P58281\|P50516\|Q91VR5\|Q9ERD7\|P62334\|P10126\|P99024\|Q8CGK3\|Q8K1M6\|Q9D5T0\|P46460\|Q8VDN2\|P62192 |
| 16462 | 2.61E-15 | 7.84E-14 | pyrophosphatase activity | 8.32% | 2.06% | P58252\|P62631\|Q99JI6\|Q01853\|P63017\|P68372\|P61021\|P62196\|O70133\|Q03265\|P61222\|P62874\|Q9Z1N5\|Q8VDD5\|P60122\|Q05D44\|Q9D3D9\|P62827\|Q9QUI0\|Q9R1K9\|P60766\|P56480\|P62814\|P35278\|Q9Z0N1\|P62627\|Q99KJ8\|A2AG68\|P50518\|Q8BFR5\|Q3THE2\|P58281\|P50516\|Q91VR5\|Q9ERD7\|P62334\|P10126\|Q9D819\|P99024\|Q8CGK3\|Q8K1M6\|Q9D5T0\|P46460\|Q8VDN2\|P62192 |
| 16818 | 3.13E-15 | 9.04E-14 | hydrolase activity, acting on acid anhydrides, in phosphorus-containing anhydrides | 8.32% | 2.07% | P58252\|P62631\|Q99JI6\|Q01853\|P63017\|P68372\|P61021\|P62196\|O70133\|Q03265\|P61222\|P62874\|Q9Z1N5\|Q8VDD5\|P60122\|Q05D44\|Q9D3D9\|P62827\|Q9QUI0\|Q9R1K9\|P60766\|P56480\|P62814\|P35278\|Q9Z0N1\|P62627\|Q99KJ8\|A2AG68\|P50518\|Q8BFR5\|Q3THE2\|P58281\|P50516\|Q91VR5\|Q9ERD7\|P62334\|P10126\|Q9D819\|P99024\|Q8CGK3\|Q8K1M6\|Q9D5T0\|P46460\|Q8VDN2\|P62192 |
| 16817 | 3.33E-15 | 9.24E-14 | hydrolase activity, acting on acid anhydrides | 8.32% | 2.07% | P58252\|P62631\|Q99JI6\|Q01853\|P63017\|P68372\|P61021\|P62196\|O70133\|Q03265\|P61222\|P62874\|Q9Z1N5\|Q8VDD5\|P60122\|Q05D44\|Q9D3D9\|P62827\|Q9QUI0\|Q9R1K9\|P60766\|P56480\|P62814\|P35278\|Q9Z0N1\|P62627\|Q99KJ8\|A2AG68\|P50518\|Q8BFR5\|Q3THE2\|P58281\|P50516\|Q91VR5\|Q9ERD7\|P62334\|P10126\|Q9D819\|P99024\|Q8CGK3\|Q8K1M6\|Q9D5T0\|P46460\|Q8VDN2\|P62192 |
| 3743 | 1.26E-14 | 3.36E-13 | translation initiation factor activity | 2.96% | 0.21% | P60229\|Q9DBZ5\|Q6NZJ6\|Q9Z1D1\|Q99JX4\|Q9Z0N1\|P23116\|Q9WUK2\|Q9QZD9\|Q8BJW6\|Q8R1B4\|Q91WK2\|Q6ZWX6\|P59325\|O55135\|Q05D44 |
| 48037 | 4.62E-14 | 1.19E-12 | cofactor binding | 4.99% | 0.80% | P54071\|Q9DCD0\|Q9CZN7\|Q61024\|Q8BMS1\|P14152\|Q8QZT1\|Q8K2B3\|P37040\|Q99LC5\|Q9JMH6\|P00375\|P50247\|Q9DCN2\|P05202\|Q61425\|O88712\|Q99K85\|Q61753\|Q921K2\|Q8BKZ9\|Q9D6R2\|P29758\|P40142\|Q9D6J6\|P19096\|Q8BMF4 |
| 16787 | 2.00E-13 | 4.97E-12 | hydrolase activity | 16.45% | 7.21% | P70349\|P61290\|Q99JI6\|P58321\|P63017\|Q9Z2U1\|P97449\|P68372\|P62137\|Q8VDD5\|P60122\|O35593\|Q9D3D9\|P62827\|Q9QUI0\|Q9R1K9\|P18242\|P50247\|P56480\|P62814\|P62141\|P62627\|Q99KJ8\|P18155\|Q9CZ30\|Q9EP69\|O09061\|Q8BFR5\|Q3THE2\|P99026\|Q91VR5\|Q9CWJ9\|Q8BZA9\|P10126\|P99024\|Q78PY7\|Q8K1M6\|Q9D5T0\|P19096\|P46460\|P62192\|P58252\|P62631\|Q9DB77\|Q01853\|O08553\|Q6P1J1\|P61021\|Q9CZ13\|O70133\|P62196\|P56399\|Q03265\|P61222\|P62874\|Q9Z1N5\|Q60710\|Q05D44\|Q9R1P0\|O54984\|Q9R1P1\|Q9R0P9\|Q8C1A5\|P60766\|O08663\|Q8K1R3\|Q6P1B1\|P35278\|O09106\|Q9Z0N1\|P31938\|Q9DBJ1\|Q9DCC4\|A2AG68\|Q9QUR6\|Q61074\|P50518\|Q61469\|P58281\|P50516\|Q9WV54\|Q9ERD7\|P28352\|P62334\|Q91V12\|Q9JM14\|Q9D819\|Q8CGK3\|Q8VDN2 |
| 3746 | 3.50E-12 | 8.42E-11 | translation elongation factor activity | 2.03% | 0.10% | Q9CZR8\|P58252\|P57776\|P62631\|P10126\|O70251\|P10711\|Q9D8N0\|Q9D1M4\|P63242\|Q8BFR5 |
| 50662 | 6.18E-12 | 1.44E-10 | coenzyme binding | 3.88% | 0.57% | Q9DCN2\|P54071\|Q9DCD0\|Q61425\|Q8BMS1\|O88712\|P14152\|Q61753\|Q921K2\|Q8QZT1\|Q9D6R2\|Q8BKZ9\|P40142\|Q9D6J6\|P37040\|Q8K2B3\|Q99LC5\|Q8BMF4\|Q9JMH6\|P00375\|P50247 |
| 3924 | 9.39E-11 | 2.12E-09 | GTPase activity | 3.33% | 0.47% | P58252\|P62631\|Q99JI6\|P35278\|Q9Z0N1\|P68372\|P61021\|Q8BFR5\|P58281\|P62874\|Q9ERD7\|P10126\|Q05D44\|P99024\|Q8K1M6\|P62827\|Q9QUI0\|P60766 |
| 16874 | 1.74E-10 | 3.80E-09 | ligase activity | 5.18% | 1.22% | Q9WTX5\|P32921\|Q8BP47\|Q61024\|P61957\|Q91WQ3\|Q91V92\|P61079\|Q64737\|Q5SUR0\|P16460\|Q9ER72\|Q9Z1F9\|Q7TMY8\|Q02053\|Q9R0P9\|Q8BMJ2\|Q9R1T2\|Q68FL6\|Q3THK7\|P46935\|Q8CGC7\|P61089\|Q8BGQ7\|Q9CZD3\|P46664\|Q9D0I9\|Q9D0R2 |
| 16616 | 2.16E-10 | 4.58E-09 | oxidoreductase activity, acting on the CH-OH group of donors, NAD or NADP as acceptor | 2.96% | 0.38% | P54071\|Q9DCD0\|Q61425\|Q8BMS1\|O88712\|P14152\|O70503\|Q61753\|P08249\|Q9D6R2\|P51660\|P45376\|P19096\|Q91VA7\|P24547\|P28474 |
| 16875 | 2.96E-10 | 5.77E-09 | ligase activity, forming carbon-oxygen bonds | 2.03% | 0.15% | Q9CZD3\|P32921\|Q8BP47\|Q9ER72\|Q91WQ3\|Q8CGC7\|Q8BGQ7\|Q9D0I9\|Q8BMJ2\|Q9D0R2\|Q68FL6 |
| 16876 | 2.96E-10 | 5.77E-09 | ligase activity, forming aminoacyl-tRNA and related compounds | 2.03% | 0.15% | Q9CZD3\|P32921\|Q8BP47\|Q9ER72\|Q91WQ3\|Q8CGC7\|Q8BGQ7\|Q9D0I9\|Q8BMJ2\|Q9D0R2\|Q68FL6 |
| 4812 | 2.96E-10 | 5.77E-09 | aminoacyl-tRNA ligase activity | 2.03% | 0.15% | Q9CZD3\|P32921\|Q8BP47\|Q9ER72\|Q91WQ3\|Q8CGC7\|Q8BGQ7\|Q9D0I9\|Q8BMJ2\|Q9D0R2\|Q68FL6 |
| 32403 | 6.25E-10 | 1.19E-08 | protein complex binding | 4.07% | 0.81% | P63038\|Q9DB77\|Q99P72\|O09106\|Q9D1D4\|Q99L47\|Q9CZ13\|Q9QYB1\|Q9DB20\|P40240\|Q91WK2\|P05132\|P34884\|P63101\|P99028\|P28352\|P62259\|P99024\|Q62261\|Q9D3D9\|P25444\|Q9R1K9 |
| 16614 | 8.20E-10 | 1.52E-08 | oxidoreductase activity, acting on CH-OH group of donors | 2.96% | 0.41% | P54071\|Q9DCD0\|Q61425\|Q8BMS1\|O88712\|P14152\|O70503\|Q61753\|P08249\|Q9D6R2\|P51660\|P45376\|P19096\|Q91VA7\|P24547\|P28474 |
| 51287 | 1.07E-09 | 1.93E-08 | NAD or NADH binding | 2.03% | 0.17% | Q61753\|Q9DCN2\|P54071\|Q921K2\|Q9D6R2\|Q9D6J6\|Q61425\|Q8BMS1\|O88712\|P14152\|P50247 |
| 15078 | 1.10E-09 | 1.93E-08 | hydrogen ion transmembrane transporter activity | 2.40% | 0.26% | Q03265\|P56480\|Q9CQQ7\|P99028\|P50516\|Q06185\|Q9CQB4\|P00405\|P62814\|P12787\|Q9D3D9\|P50518\|Q9DB20 |
| 43022 | 4.35E-09 | 7.46E-08 | ribosome binding | 1.29% | 0.05% | P20029\|Q9DBG6\|Q9DBZ5\|O55135\|P61620\|Q8R1B4\|P63242 |
| 31072 | 6.43E-09 | 1.08E-07 | heat shock protein binding | 2.22% | 0.24% | Q61081\|Q60864\|Q6NZB0\|Q99MD9\|P30416\|Q68FD5\|Q9QYJ0\|Q07813\|P11440\|Q99KV1\|Q99M87\|Q99L47 |
| 46933 | 8.20E-09 | 1.34E-07 | hydrogen ion transporting ATP synthase activity, rotational mechanism | 1.11% | 0.03% | Q03265\|P56480\|P50516\|P62814\|Q9D3D9\|Q9DB20 |
| 43021 | 9.05E-09 | 1.45E-07 | ribonucleoprotein binding | 1.66% | 0.12% | P20029\|Q9DBG6\|Q9DBZ5\|O55135\|Q61937\|P61620\|Q8R1B4\|P62137\|P63242 |
| 43531 | 1.27E-08 | 1.99E-07 | ADP binding | 1.29% | 0.06% | Q9DCN2\|Q03265\|P52480\|Q8VDD5\|P63017\|Q8VDN2\|P09411 |
| 42802 | 2.84E-08 | 4.35E-07 | identical protein binding | 4.62% | 1.25% | Q9D0K2\|Q9CZN7\|Q61024\|Q01853\|Q99L47\|P35700\|Q99KQ4\|P80313\|P12815\|P16045\|Q8QZT1\|P07901\|P14148\|P28658\|P50247\|P05202\|P20108\|Q8BJU0\|P17182\|Q9DCC4\|P62627\|O88712\|P10518\|Q07813\|P28474 |
| 8565 | 3.18E-08 | 4.77E-07 | protein transporter activity | 2.03% | 0.22% | Q9EPL8\|Q6P5F9\|Q6NZD2\|P70168\|O35343\|P52293\|Q9ERK4\|O35345\|Q8BKC5\|P61620\|Q60960 |
| 51920 | 4.57E-08 | 6.73E-07 | peroxiredoxin activity | 0.92% | 0.02% | Q61171\|P20108\|O08807\|Q99LX0\|P35700 |
| 46961 | 6.38E-08 | 9.21E-07 | proton-transporting ATPase activity, rotational mechanism | 1.11% | 0.04% | Q03265\|P56480\|P50516\|P62814\|Q9D3D9\|P50518 |
| 19843 | 1.03E-07 | 1.45E-06 | rRNA binding | 1.29% | 0.08% | P62270\|P62918\|P62281\|P63158\|Q61937\|Q6ZWN5\|P62702 |
| 15077 | 2.86E-07 | 3.96E-06 | monovalent inorganic cation transmembrane transporter activity | 2.59% | 0.47% | Q03265\|P56480\|Q9CQQ7\|P99028\|P50516\|Q06185\|Q9CQB4\|P00405\|P62814\|P12787\|Q9D3D9\|P50518\|Q8VDN2\|Q9DB20 |
| 16887 | 3.43E-07 | 4.67E-06 | ATPase activity | 3.33% | 0.79% | P56480\|Q01853\|P62814\|P63017\|A2AG68\|O70133\|P50518\|P61222\|Q03265\|P50516\|Q9Z1N5\|Q91VR5\|Q8VDD5\|Q8CGK3\|Q9D3D9\|Q9R1K9\|P46460\|Q8VDN2 |
| 42623 | 6.05E-07 | 8.08E-06 | ATPase activity, coupled | 2.96% | 0.65% | P56480\|P62814\|P63017\|A2AG68\|O70133\|P50518\|Q03265\|P50516\|Q9Z1N5\|Q91VR5\|Q8VDD5\|Q8CGK3\|Q9D3D9\|Q9R1K9\|P46460\|Q8VDN2 |
| 19899 | 1.23E-06 | 1.61E-05 | enzyme binding | 4.25% | 1.33% | Q3V0I7\|P07356\|P63038\|P05202\|Q3THK7\|P08113\|P31938\|O35900\|P26645\|P70296\|P05132\|P62962\|O55029\|Q8QZT1\|P60710\|P37040\|P62259\|Q7TSZ0\|P47758\|Q61937\|P68040\|P60766\|B7FAU9 |
| 31406 | 1.37E-06 | 1.76E-05 | carboxylic acid binding | 2.40% | 0.46% | Q9DCD0\|P05202\|Q9CZN7\|Q8BKZ9\|P07724\|Q8BMS1\|Q9R0E1\|Q8BMF4\|P19096\|P47856\|Q8BGQ7\|Q9D0I9\|Q60716 |
| 22890 | 1.65E-06 | 2.09E-05 | inorganic cation transmembrane transporter activity | 2.77% | 0.62% | Q03265\|P56480\|Q9CQQ7\|P99028\|P50516\|Q06185\|Q9CQB4\|P00405\|P62814\|P12787\|Q9D3D9\|A2AG68\|P50518\|Q8VDN2\|Q9DB20 |
| 19829 | 1.78E-06 | 2.21E-05 | cation-transporting ATPase activity | 1.11% | 0.07% | Q03265\|P56480\|P50516\|P62814\|Q9D3D9\|P50518 |
| 16853 | 3.86E-06 | 4.72E-05 | isomerase activity | 2.22% | 0.43% | Q9CR16\|P27773\|P26883\|P34884\|Q9D0F9\|P30416\|Q922R8\|P51660\|P17751\|P06745\|Q9DBJ1\|P09103 |
| 16209 | 7.04E-06 | 8.46E-05 | antioxidant activity | 1.29% | 0.13% | O08709\|Q61171\|P20108\|O08807\|P08228\|P35700\|Q9JMH6 |
| 16862 | 7.97E-06 | 9.43E-05 | intramolecular oxidoreductase activity, interconverting keto- and enol-groups | 0.74% | 0.03% | P27773\|P34884\|Q922R8\|P09103 |
| 51087 | 2.10E-05 | 2.44E-04 | chaperone binding | 0.92% | 0.07% | Q60864\|Q8BK64\|Q07813\|P08228\|Q99L47 |
| 42625 | 2.23E-05 | 2.55E-04 | ATPase activity, coupled to transmembrane movement of ions | 1.48% | 0.22% | Q03265\|P56480\|P50516\|P62814\|Q9D3D9\|A2AG68\|P50518\|Q8VDN2 |
| 16829 | 2.53E-05 | 2.85E-04 | lyase activity | 2.22% | 0.52% | Q9CPU0\|Q99KI0\|Q9CZN7\|P28352\|Q91YI0\|P51660\|Q8BMS1\|P17182\|Q91V92\|P19096\|P13439\|P10518 |
| 8092 | 3.01E-05 | 3.34E-04 | cytoskeletal protein binding | 3.88% | 1.40% | P07356\|Q9CQI6\|P30416\|Q99JY9\|P18760\|Q9JM76\|P26645\|Q9WUM4\|P45591\|P54227\|P62962\|P47753\|P61161\|P15532\|Q8VDD5\|Q61553\|Q68FD5\|P68510\|Q62261\|B7FAU9\|P26040 |
| 22892 | 3.44E-05 | 3.76E-04 | substrate-specific transporter activity | 6.10% | 2.82% | Q06185\|Q60931\|Q60932\|Q8BKC5\|Q9QYB1\|P53811\|Q03265\|P24668\|Q9CQB4\|P00405\|Q6P5F9\|Q8BG05\|Q6NZD2\|O35343\|Q9D3D9\|P52293\|O35345\|Q60960\|P56480\|Q9EPL8\|P62814\|P12787\|Q9ERK4\|A2AG68\|P61620\|P50518\|Q9DB20\|P50516\|P99028\|Q9CQQ7\|P70168\|P19096\|Q8VDN2 |
| 4298 | 3.56E-05 | 3.78E-04 | threonine-type endopeptidase activity | 0.92% | 0.07% | P99026\|Q9R1P0\|Q9Z2U1\|Q9R1P1\|O09061 |
| 70003 | 3.56E-05 | 3.78E-04 | threonine-type peptidase activity | 0.92% | 0.07% | P99026\|Q9R1P0\|Q9Z2U1\|Q9R1P1\|O09061 |
| 16860 | 4.26E-05 | 4.45E-04 | intramolecular oxidoreductase activity | 1.11% | 0.12% | P27773\|P34884\|Q922R8\|P17751\|P06745\|P09103 |
| 16835 | 4.97E-05 | 5.12E-04 | carbon-oxygen lyase activity | 1.29% | 0.18% | Q99KI0\|P28352\|P51660\|Q8BMS1\|P17182\|P19096\|P10518 |
| 4448 | 6.32E-05 | 6.39E-04 | isocitrate dehydrogenase activity | 0.55% | 0.02% | P54071\|Q9D6R2\|Q91VA7 |
| 5215 | 6.38E-05 | 6.39E-04 | transporter activity | 6.84% | 3.43% | Q06185\|Q60931\|Q60932\|Q8BKC5\|Q9QYB1\|Q8BH59\|P53811\|Q03265\|P24668\|Q9CQB4\|P00405\|Q6P5F9\|Q8BG05\|Q6NZD2\|O35343\|Q9D3D9\|P52293\|O35345\|Q8VEM8\|Q8BHH1\|Q60960\|P56480\|Q9EPL8\|P62814\|P12787\|Q9ERK4\|A2AG68\|P61620\|P50518\|Q9DB20\|P48962\|P50516\|P99028\|Q9CQQ7\|P70168\|P19096\|Q8VDN2 |
| 16740 | 6.72E-05 | 6.64E-04 | transferase activity | 9.80% | 5.63% | Q9D0K2\|Q9WTX5\|Q9CZN7\|Q8BMS1\|P11440\|Q8BHC4\|P47856\|Q91YM4\|Q62318\|Q64737\|Q8BTZ7\|Q5SUR0\|Q8QZT1\|Q3THS6\|P19157\|Q91ZJ5\|Q99K85\|P09411\|Q9CWJ9\|P29758\|P19096\|Q8BMF4\|Q9CZU6\|Q9CXY6\|Q921H8\|P52480\|Q8R574\|Q91V92\|Q99KQ4\|P13439\|Q9DBG6\|P15532\|O08795\|Q8K224\|Q6PK77\|Q920E5\|Q3V0I7\|P05202\|Q9DCG9\|Q9WTP6\|Q8K1R3\|Q9CQ65\|Q7TPV4\|P31938\|Q7TT37\|Q93092\|P05132\|Q921K2\|Q8BKZ9\|Q6P5E4\|P40142\|Q8CIG8\|Q64674 |
| 42803 | 7.18E-05 | 7.00E-04 | protein homodimerization activity | 2.77% | 0.85% | Q9D0K2\|P05202\|Q61024\|Q8BJU0\|P17182\|P35700\|O88712\|Q99KQ4\|P12815\|P16045\|P07901\|Q8QZT1\|Q07813\|P14148\|P28474 |
| 15405 | 7.39E-05 | 7.10E-04 | P-P-bond-hydrolysis-driven transmembrane transporter activity | 1.66% | 0.33% | Q03265\|P56480\|P50516\|P62814\|Q9D3D9\|A2AG68\|P61620\|P50518\|Q8VDN2 |
| 15399 | 8.02E-05 | 7.56E-04 | primary active transmembrane transporter activity | 1.66% | 0.33% | Q03265\|P56480\|P50516\|P62814\|Q9D3D9\|A2AG68\|P61620\|P50518\|Q8VDN2 |
| 3729 | 8.07E-05 | 7.56E-04 | mRNA binding | 1.29% | 0.19% | Q62348\|P14211\|P14148\|Q8BG05\|Q3U0V1\|Q61584\|P62960 |
| 19901 | 8.28E-05 | 7.65E-04 | protein kinase binding | 2.03% | 0.49% | Q3V0I7\|P70296\|P05132\|O55029\|P60710\|P68040\|P31938\|O35900\|P26645\|B7FAU9\|P60766 |
| 16879 | 9.15E-05 | 8.35E-04 | ligase activity, forming carbon-nitrogen bonds | 2.22% | 0.59% | Q5SUR0\|P16460\|Q9WTX5\|Q3THK7\|Q61024\|Q7TMY8\|P61957\|P46664\|P46935\|P61089\|P61079\|Q64737 |
| 16836 | 9.30E-05 | 8.38E-04 | hydro-lyase activity | 1.11% | 0.14% | Q99KI0\|P51660\|Q8BMS1\|P17182\|P19096\|P10518 |
| 16646 | 1.04E-04 | 9.28E-04 | oxidoreductase activity, acting on the CH-NH group of donors, NAD or NADP as acceptor | 0.74% | 0.05% | Q8R127\|P18155\|Q9DCC4\|P00375 |
| 30544 | 1.25E-04 | 1.10E-03 | Hsp70 protein binding | 0.55% | 0.02% | Q60864\|P11440\|Q99L47 |
| 4601 | 1.84E-04 | 1.58E-03 | peroxidase activity | 0.92% | 0.10% | O08709\|Q61171\|P20108\|O08807\|P35700 |
| 16684 | 1.84E-04 | 1.58E-03 | oxidoreductase activity, acting on peroxide as acceptor | 0.92% | 0.10% | O08709\|Q61171\|P20108\|O08807\|P35700 |
| 16705 | 1.96E-04 | 1.66E-03 | oxidoreductase activity, acting on paired donors, with incorporation or reduction of molecular oxygen | 1.85% | 0.46% | Q61171\|P20108\|O08807\|Q99LN9\|Q99LX0\|Q9R0E1\|P35700\|O70252\|P09103\|Q60716 |
| 19900 | 2.12E-04 | 1.74E-03 | kinase binding | 2.03% | 0.55% | Q3V0I7\|P70296\|P05132\|O55029\|P60710\|P68040\|P31938\|O35900\|P26645\|B7FAU9\|P60766 |
| 16864 | 2.15E-04 | 1.74E-03 | intramolecular oxidoreductase activity, transposing S-S bonds | 0.55% | 0.02% | P27773\|Q922R8\|P09103 |
| 3756 | 2.15E-04 | 1.74E-03 | protein disulfide isomerase activity | 0.55% | 0.02% | P27773\|Q922R8\|P09103 |
| 3857 | 2.15E-04 | 1.74E-03 | 3-hydroxyacyl-CoA dehydrogenase activity | 0.55% | 0.02% | Q61425\|P51660\|Q8BMS1 |
| 16820 | 2.27E-04 | 1.80E-03 | hydrolase activity, acting on acid anhydrides, catalyzing transmembrane movement of substances | 1.48% | 0.30% | Q03265\|P56480\|P50516\|P62814\|Q9D3D9\|A2AG68\|P50518\|Q8VDN2 |
| 42626 | 2.27E-04 | 1.80E-03 | ATPase activity, coupled to transmembrane movement of substances | 1.48% | 0.30% | Q03265\|P56480\|P50516\|P62814\|Q9D3D9\|A2AG68\|P50518\|Q8VDN2 |
| 3779 | 2.34E-04 | 1.83E-03 | actin binding | 2.77% | 0.95% | P45591\|P62962\|P61161\|P47753\|Q9CQI6\|Q8VDD5\|Q61553\|Q99JY9\|P68510\|Q62261\|P18760\|Q9JM76\|P26645\|B7FAU9\|Q9WUM4 |
| 43492 | 2.46E-04 | 1.91E-03 | ATPase activity, coupled to movement of substances | 1.48% | 0.30% | Q03265\|P56480\|P50516\|P62814\|Q9D3D9\|A2AG68\|P50518\|Q8VDN2 |
| 3697 | 2.55E-04 | 1.96E-03 | single-stranded DNA binding | 0.92% | 0.11% | P61979\|Q6PB66\|P63158\|Q8CGK3\|P62960 |
| 4372 | 3.40E-04 | 2.49E-03 | glycine hydroxymethyltransferase activity | 0.55% | 0.03% | Q9CWJ9\|Q9CZN7\|Q64737 |
| 8641 | 3.40E-04 | 2.49E-03 | small protein activating enzyme activity | 0.55% | 0.03% | Q9Z1F9\|Q02053\|Q9R1T2 |
| 51538 | 3.49E-04 | 2.49E-03 | 3 iron, 4 sulfur cluster binding | 0.37% | 0.01% | Q99KI0\|Q9CQA3 |
| 16635 | 3.49E-04 | 2.49E-03 | oxidoreductase activity, acting on the CH-CH group of donors, quinone or related compound as acceptor | 0.37% | 0.01% | Q8K2B3\|Q9CQA3 |
| 16418 | 3.49E-04 | 2.49E-03 | S-acetyltransferase activity | 0.37% | 0.01% | Q8BMF4\|P19096 |
| 8177 | 3.49E-04 | 2.49E-03 | succinate dehydrogenase (ubiquinone) activity | 0.37% | 0.01% | Q8K2B3\|Q9CQA3 |
| 30060 | 3.49E-04 | 2.49E-03 | L-malate dehydrogenase activity | 0.37% | 0.01% | P08249\|P14152 |
| 48029 | 4.08E-04 | 2.89E-03 | monosaccharide binding | 1.11% | 0.18% | Q93092\|Q91ZJ5\|P16045\|P24668\|P40142\|P06745 |
| 16763 | 4.58E-04 | 3.20E-03 | transferase activity, transferring pentosyl groups | 0.92% | 0.12% | Q921K2\|Q9CQ65\|Q6PK77\|Q99KQ4\|P13439 |
| 49 | 4.62E-04 | 3.20E-03 | tRNA binding | 0.74% | 0.07% | Q91WQ3\|Q8BGQ7\|Q9D0I9\|Q68FL6 |
| 16884 | 5.02E-04 | 3.45E-03 | carbon-nitrogen ligase activity, with glutamine as amido-N-donor | 0.55% | 0.03% | Q5SUR0\|Q3THK7\|Q61024 |
| 8289 | 5.25E-04 | 3.57E-03 | lipid binding | 2.96% | 1.14% | P07356\|P05202\|P30681\|P63158\|Q01853\|Q8BMS1\|Q9CZ44\|Q9DB20\|P53811\|P70296\|P48036\|P62962\|P07724\|P51660\|Q61937\|Q6NZD2 |
| 9055 | 6.69E-04 | 4.48E-03 | electron carrier activity | 1.48% | 0.35% | P61222\|Q9DCW4\|P00405\|P62897\|Q8K2B3\|Q91VD9\|Q99LC5\|Q9CQA3 |
| 16597 | 6.73E-04 | 4.48E-03 | amino acid binding | 1.11% | 0.20% | P05202\|Q9CZN7\|P19096\|P47856\|Q8BGQ7\|Q9D0I9 |
| 8483 | 6.77E-04 | 4.48E-03 | transaminase activity | 0.74% | 0.08% | P05202\|P29758\|P47856\|Q99K85 |
| 5507 | 7.65E-04 | 5.01E-03 | copper ion binding | 0.92% | 0.13% | O08997\|P00405\|P08228\|A2AG68\|P50247 |
| 16645 | 8.07E-04 | 5.24E-03 | oxidoreductase activity, acting on the CH-NH group of donors | 0.74% | 0.08% | Q8R127\|P18155\|Q9DCC4\|P00375 |
| 5080 | 9.54E-04 | 6.14E-03 | protein kinase C binding | 0.74% | 0.08% | O55029\|P68040\|P26645\|B7FAU9 |
| 4449 | 1.03E-03 | 6.43E-03 | isocitrate dehydrogenase (NAD+) activity | 0.37% | 0.01% | Q9D6R2\|Q91VA7 |
| 104 | 1.03E-03 | 6.43E-03 | succinate dehydrogenase activity | 0.37% | 0.01% | Q8K2B3\|Q9CQA3 |
| 3985 | 1.03E-03 | 6.43E-03 | acetyl-CoA C-acetyltransferase activity | 0.37% | 0.01% | Q921H8\|Q8QZT1 |
| 51787 | 1.03E-03 | 6.43E-03 | misfolded protein binding | 0.37% | 0.01% | P63038\|P20029 |
| 46983 | 1.24E-03 | 7.62E-03 | protein dimerization activity | 3.33% | 1.47% | Q9D0K2\|P63038\|P05202\|P63158\|Q61024\|Q8BJU0\|P17182\|P35700\|O88712\|Q99KQ4\|P12815\|P16045\|P07901\|Q8QZT1\|Q9WV55\|Q07813\|P14148\|P28474 |
| 287 | 1.55E-03 | 9.44E-03 | magnesium ion binding | 1.48% | 0.40% | P54071\|P52480\|Q9D0F9\|Q8R574\|Q9D6R2\|P40142\|Q9D819\|P46664 |
| 16408 | 1.62E-03 | 9.80E-03 | C-acyltransferase activity | 0.55% | 0.04% | Q921H8\|Q8QZT1\|Q8BMS1 |
| 19842 | 1.92E-03 | 1.14E-02 | vitamin binding | 1.48% | 0.41% | P05202\|Q9CZN7\|P29758\|P40142\|Q9R0E1\|P19096\|Q99K85\|Q60716 |
| 16651 | 1.98E-03 | 1.14E-02 | oxidoreductase activity, acting on NADH or NADPH | 0.92% | 0.17% | Q9DCN2\|Q9D6J6\|P37040\|Q91VD9\|Q9JMH6 |
| 50661 | 1.98E-03 | 1.14E-02 | NADP or NADPH binding | 0.74% | 0.10% | Q9DCD0\|P37040\|Q9JMH6\|P00375 |
| 31545 | 2.04E-03 | 1.14E-02 | peptidyl-proline 4-dioxygenase activity | 0.37% | 0.01% | P09103\|Q60716 |
| 16453 | 2.04E-03 | 1.14E-02 | C-acetyltransferase activity | 0.37% | 0.01% | Q921H8\|Q8QZT1 |
| 5527 | 2.04E-03 | 1.14E-02 | macrolide binding | 0.37% | 0.01% | Q9CR16\|P26883 |
| 5528 | 2.04E-03 | 1.14E-02 | FK506 binding | 0.37% | 0.01% | Q9CR16\|P26883 |
| 16744 | 2.04E-03 | 1.14E-02 | transferase activity, transferring aldehyde or ketonic groups | 0.37% | 0.01% | Q93092\|P40142 |
| 17025 | 2.04E-03 | 1.14E-02 | TATA-binding protein binding | 0.37% | 0.01% | Q6ZQ38\|P70670 |
| 4656 | 2.04E-03 | 1.14E-02 | procollagen-proline 4-dioxygenase activity | 0.37% | 0.01% | P09103\|Q60716 |
| 16769 | 2.25E-03 | 1.25E-02 | transferase activity, transferring nitrogenous groups | 0.74% | 0.10% | P05202\|P29758\|P47856\|Q99K85 |
| 51536 | 2.38E-03 | 1.30E-02 | iron-sulfur cluster binding | 0.92% | 0.17% | Q99KI0\|P61222\|Q9D6J6\|Q91VD9\|Q9CQA3 |
| 51540 | 2.38E-03 | 1.30E-02 | metal cluster binding | 0.92% | 0.17% | Q99KI0\|P61222\|Q9D6J6\|Q91VD9\|Q9CQA3 |
| 16417 | 3.36E-03 | 1.76E-02 | S-acyltransferase activity | 0.37% | 0.02% | Q8BMF4\|P19096 |
| 46912 | 3.36E-03 | 1.76E-02 | transferase activity, transferring acyl groups, acyl groups converted into alkyl on transfer | 0.37% | 0.02% | Q91V92\|Q9CZU6 |
| 4176 | 3.36E-03 | 1.76E-02 | ATP-dependent peptidase activity | 0.37% | 0.02% | Q8CGK3\|P46460 |
| 5092 | 3.36E-03 | 1.76E-02 | GDP-dissociation inhibitor activity | 0.37% | 0.02% | P50396\|Q99PT1 |
| 19238 | 3.36E-03 | 1.76E-02 | cyclohydrolase activity | 0.37% | 0.02% | Q9CWJ9\|P18155 |
| 51879 | 3.36E-03 | 1.76E-02 | Hsp90 protein binding | 0.37% | 0.02% | Q61081\|Q99MD9 |
| 46872 | 4.07E-03 | 2.11E-02 | metal ion binding | 14.60% | 10.87% | Q99KI0\|P07356\|P54071\|P35564\|P97449\|Q64737\|Q8BH59\|Q62318\|P62137\|P48036\|Q8QZT1\|Q9D0F9\|P62897\|Q99LN9\|Q80TB8\|O35593\|Q9QYJ0\|Q99M87\|O70252\|Q9R1K9\|P24547\|Q3THS6\|P50247\|Q91ZJ5\|P56480\|P12787\|P62141\|P08228\|P61211\|Q3THE2\|P10518\|Q9D6J6\|P07724\|Q9DCT8\|P46664\|P19096\|P46460\|P28474\|P52480\|Q8R574\|Q9DB77\|P35700\|Q91V92\|Q9CZ13\|P56399\|Q60716\|Q9CQX2\|O08997\|P12815\|P15532\|P14211\|Q9ER72\|P00405\|Q9D024\|O54984\|P62204\|Q8K310\|Q9R0E1\|Q920E5\|Q8C1A5\|P10711\|Q9CPU0\|O08663\|Q6P1B1\|Q8BGQ7\|A2AG68\|Q61074\|Q9WUL7\|Q921K2\|Q9D6R2\|P28352\|P40142\|Q9JM14\|Q9D819\|Q91VD9\|Q9CQA3\|Q62465\|P62075\|Q8VDN2 |
| 8233 | 4.11E-03 | 2.12E-02 | peptidase activity | 3.88% | 2.04% | O08663\|P61290\|Q9DB77\|P58321\|Q6P1B1\|Q9Z2U1\|P97449\|Q9CZ13\|Q9QUR6\|P62196\|P56399\|O09061\|P99026\|O35593\|Q9R1P0\|Q8CGK3\|Q9R1P1\|Q9R0P9\|Q8C1A5\|P46460\|P18242 |
| 16810 | 4.23E-03 | 2.16E-02 | hydrolase activity, acting on carbon-nitrogen (but not peptide) bonds | 1.29% | 0.37% | Q9CWJ9\|Q9WV54\|O08553\|O09106\|Q6P1J1\|P18155\|Q9DCC4 |
| 19904 | 4.68E-03 | 2.38E-02 | protein domain specific binding | 2.40% | 1.04% | Q3V0I7\|P68369\|P68254\|P61982\|P63101\|Q9D1J3\|P30681\|P62259\|P68510\|Q07813\|Q99L47\|Q9QUI0\|Q8VDN2 |
| 16681 | 4.98E-03 | 2.44E-02 | oxidoreductase activity, acting on diphenols and related substances as donors, cytochrome as acceptor | 0.37% | 0.02% | P99028\|Q9CQB4 |
| 16742 | 4.98E-03 | 2.44E-02 | hydroxymethyl-, formyl- and related transferase activity | 0.37% | 0.02% | Q9CWJ9\|Q64737 |
| 8121 | 4.98E-03 | 2.44E-02 | ubiquinol-cytochrome-c reductase activity | 0.37% | 0.02% | P99028\|Q9CQB4 |
| 30971 | 4.98E-03 | 2.44E-02 | receptor tyrosine kinase binding | 0.37% | 0.02% | P48036\|P61982 |
| 30955 | 4.98E-03 | 2.44E-02 | potassium ion binding | 0.37% | 0.02% | P52480\|Q8VDN2 |
| 8308 | 5.04E-03 | 2.46E-02 | voltage-gated anion channel activity | 0.55% | 0.07% | Q60931\|Q60932\|Q9QYB1 |
| 16407 | 5.26E-03 | 2.55E-02 | acetyltransferase activity | 0.92% | 0.21% | Q921H8\|Q8QZT1\|Q8K224\|Q8BMF4\|P19096 |
| 43169 | 5.37E-03 | 2.58E-02 | cation binding | 14.60% | 10.99% | Q99KI0\|P07356\|P54071\|P35564\|P97449\|Q64737\|Q8BH59\|Q62318\|P62137\|P48036\|Q8QZT1\|Q9D0F9\|P62897\|Q99LN9\|Q80TB8\|O35593\|Q9QYJ0\|Q99M87\|O70252\|Q9R1K9\|P24547\|Q3THS6\|P50247\|Q91ZJ5\|P56480\|P12787\|P62141\|P08228\|P61211\|Q3THE2\|P10518\|Q9D6J6\|P07724\|Q9DCT8\|P46664\|P19096\|P46460\|P28474\|P52480\|Q8R574\|Q9DB77\|P35700\|Q91V92\|Q9CZ13\|P56399\|Q60716\|Q9CQX2\|O08997\|P12815\|P15532\|P14211\|Q9ER72\|P00405\|Q9D024\|O54984\|P62204\|Q8K310\|Q9R0E1\|Q920E5\|Q8C1A5\|P10711\|Q9CPU0\|O08663\|Q6P1B1\|Q8BGQ7\|A2AG68\|Q61074\|Q9WUL7\|Q921K2\|Q9D6R2\|P28352\|P40142\|Q9JM14\|Q9D819\|Q91VD9\|Q9CQA3\|Q62465\|P62075\|Q8VDN2 |
| 43167 | 5.76E-03 | 2.75E-02 | ion binding | 14.60% | 11.02% | Q99KI0\|P07356\|P54071\|P35564\|P97449\|Q64737\|Q8BH59\|Q62318\|P62137\|P48036\|Q8QZT1\|Q9D0F9\|P62897\|Q99LN9\|Q80TB8\|O35593\|Q9QYJ0\|Q99M87\|O70252\|Q9R1K9\|P24547\|Q3THS6\|P50247\|Q91ZJ5\|P56480\|P12787\|P62141\|P08228\|P61211\|Q3THE2\|P10518\|Q9D6J6\|P07724\|Q9DCT8\|P46664\|P19096\|P46460\|P28474\|P52480\|Q8R574\|Q9DB77\|P35700\|Q91V92\|Q9CZ13\|P56399\|Q60716\|Q9CQX2\|O08997\|P12815\|P15532\|P14211\|Q9ER72\|P00405\|Q9D024\|O54984\|P62204\|Q8K310\|Q9R0E1\|Q920E5\|Q8C1A5\|P10711\|Q9CPU0\|O08663\|Q6P1B1\|Q8BGQ7\|A2AG68\|Q61074\|Q9WUL7\|Q921K2\|Q9D6R2\|P28352\|P40142\|Q9JM14\|Q9D819\|Q91VD9\|Q9CQA3\|Q62465\|P62075\|Q8VDN2 |
| 51537 | 5.85E-03 | 2.76E-02 | 2 iron, 2 sulfur cluster binding | 0.55% | 0.07% | Q9D6J6\|Q91VD9\|Q9CQA3 |
| 42393 | 5.85E-03 | 2.76E-02 | histone binding | 0.55% | 0.07% | P30681\|Q99MD9\|Q9EPL8 |
| 5529 | 6.03E-03 | 2.82E-02 | sugar binding | 1.85% | 0.72% | Q93092\|Q91ZJ5\|P16045\|Q9DCD0\|P24668\|P14211\|P40142\|P35564\|P06745\|P47856 |
| 16615 | 6.89E-03 | 3.10E-02 | malate dehydrogenase activity | 0.37% | 0.02% | P08249\|P14152 |
| 31543 | 6.89E-03 | 3.10E-02 | peptidyl-proline dioxygenase activity | 0.37% | 0.02% | P09103\|Q60716 |
| 19798 | 6.89E-03 | 3.10E-02 | procollagen-proline dioxygenase activity | 0.37% | 0.02% | P09103\|Q60716 |
| 16653 | 6.89E-03 | 3.10E-02 | oxidoreductase activity, acting on NADH or NADPH, heme protein as acceptor | 0.37% | 0.02% | Q9DCN2\|P37040 |
| 16679 | 6.89E-03 | 3.10E-02 | oxidoreductase activity, acting on diphenols and related substances as donors | 0.37% | 0.02% | P99028\|Q9CQB4 |
| 31405 | 6.89E-03 | 3.10E-02 | lipoic acid binding | 0.37% | 0.02% | Q8BKZ9\|Q8BMF4 |
| 8022 | 7.38E-03 | 3.31E-02 | protein C-terminus binding | 0.92% | 0.22% | Q60864\|Q9D1J3\|O35381\|P46935\|P46460 |
| 51539 | 8.72E-03 | 3.86E-02 | 4 iron, 4 sulfur cluster binding | 0.55% | 0.08% | Q99KI0\|Q91VD9\|Q9CQA3 |
| 16706 | 8.72E-03 | 3.86E-02 | oxidoreductase activity, acting on paired donors, with incorporation or reduction of molecular oxygen, 2-oxoglutarate as one donor, and incorporation of one atom each of oxygen into both donors | 0.55% | 0.08% | Q9R0E1\|P09103\|Q60716 |
| 16746 | 8.89E-03 | 3.89E-02 | transferase activity, transferring acyl groups | 1.66% | 0.65% | Q921H8\|Q8QZT1\|Q8BKZ9\|Q8BMS1\|Q8K224\|Q8BMF4\|Q91V92\|P19096\|Q9CZU6 |
| 50660 | 8.90E-03 | 3.89E-02 | FAD binding | 0.92% | 0.24% | Q9DCN2\|P37040\|Q8K2B3\|Q99LC5\|Q9JMH6 |
| 70011 | 9.65E-03 | 4.19E-02 | peptidase activity, acting on L-amino acid peptides | 3.51% | 1.93% | O08663\|Q9DB77\|P58321\|Q6P1B1\|Q9Z2U1\|P97449\|Q9CZ13\|Q9QUR6\|P56399\|O09061\|P99026\|O35593\|Q9R1P0\|Q8CGK3\|Q9R1P1\|Q9R0P9\|Q8C1A5\|P46460\|P18242 |
| 5543 | 1.00E-02 | 4.34E-02 | phospholipid binding | 1.29% | 0.44% | P48036\|P53811\|P07356\|P62962\|P05202\|Q61937\|Q6NZD2 |
| 16790 | 1.13E-02 | 4.85E-02 | thiolester hydrolase activity | 1.11% | 0.35% | Q91V12\|P58321\|O35593\|P19096\|Q9R0P9\|P56399 |
| 5546 | 1.15E-02 | 4.88E-02 | phosphatidylinositol-4,5-bisphosphate binding | 0.37% | 0.03% | P07356\|P62962 |
| 16861 | 1.15E-02 | 4.88E-02 | intramolecular oxidoreductase activity, interconverting aldoses and ketoses | 0.37% | 0.03% | P17751\|P06745 |
